# Necroptotic kinases are involved in the reduction of depression-induced astrocytes and fluoxetine’s inhibitory effects on necroptotic kinases

**DOI:** 10.3389/fphar.2022.1060954

**Published:** 2023-01-04

**Authors:** Salman Zeb, Huan Ye, Yuan Liu, Hua-Ping Du, Yi Guo, Yong-Ming Zhu, Yong Ni, Hui-Ling Zhang, Yuan Xu

**Affiliations:** ^1^ Jiangsu Key Laboratory of Neuropsychiatric Diseases and College of Pharmaceutical Sciences, Soochow University, Suzhou, China; ^2^ Laboratory of Cerebrovascular Pharmacology, College of Pharmaceutical Science, Soochow University, Suzhou, China; ^3^ Jiangsu Key Laboratory of Preventive and Translational Medicine for Geriatric Diseases, School of Public Health, Soochow University, Suzhou, China; ^4^ Department of Neurology, Suzhou Ninth Hospital Affiliated to Soochow University, Suzhou, China; ^5^ Pain Department, The Second Affiliated Hospital of Soochow University, Suzhou, China

**Keywords:** depression, astrocytes, necroptosis, inflammatory cytokines, fluoxetine

## Abstract

The role of astrocytes in major depressive disorder has received great attention. Increasing evidence indicates that decreased astrocyte numbers in the hippocampus may be associated with depression, but the role of necroptosis in depression is unknown. Here, in a chronic unpredictable mild stress (CUMS) mouse model and a corticosterone (Cort)-induced human astrocyte injury model *in vitro*, we found that mice treated with chronic unpredictable mild stress for 3–5 weeks presented depressive-like behaviors and reduced body weight gain, accompanied by a reduction in astrocytes and a decrease in astrocytic brain-derived neurotropic factors (BDNF), by activation of necroptotic kinases, including RIPK1 (receptor-interacting protein kinase 1)/p-RIPK1, RIPK3 (receptor-interacting protein kinase 3)/p-RIPK3 and MLKL (mixed lineage kinase domain-like protein)/p-MLKL, and by upregulation of inflammatory cytokines in astrocytes of the mouse hippocampus. In contrast, necroptotic kinase inhibitors suppressed Cort-induced necroptotic kinase activation, reduced astrocytes, astrocytic necroptosis and dysfunction, and decreased Cort-mediated inflammatory cytokines in astrocytes. Treatment with fluoxetine (FLX) for 5 weeks improved chronic unpredictable mild stress-induced mouse depressive-like behaviors; simultaneously, fluoxetine inhibited depression-induced necroptotic kinase activation, reversed the reduction in astrocytes and astrocytic necroptosis and dysfunction, decreased inflammatory cytokines and upregulated brain-derived neurotropic factors and 5-HT1A levels. Furthermore, fluoxetine had no direct inhibitory effect on receptor-interacting protein kinase 1 phosphorylation. The combined administration of fluoxetine and necroptotic kinase inhibitors further reduced corticosterone-induced astrocyte injury. In conclusion, the reduction in astrocytes caused by depressive-like models *in vivo* and *in vitro* may be associated with the activation of necroptotic kinases and astrocytic necroptosis, and fluoxetine exerts an antidepressive effect by indirectly inhibiting receptor-interacting protein kinase 1-mediated astrocytic necroptosis.

## Introduction

Depression is a chronic, recurrent and debilitating mental illness that is characterized by core symptoms such as depressed mood, loss of interest or pleasure, and patients suffering from major depressive disorder (MDD) have the propensity to suicide (Leonard, 2001; Santarelli et al., 2003). According to the World Health Organization, depression will be the second most likely leading cause of disability, mortality, and death in 2030 (Shu et al., 2019). Although the neurobiology of MDD has been intensely studied for several decades, the exact mechanism for the neuropathology of depression is still not fully understood (Trivedi et al., 2006; Krishnan and Nestler, 2010). The monoamine hypothesis is suggested to be a precise mechanism and plays a key role in the etiology of depression. Based on the monoamine hypothesis, the patient suffering from MDD has imbalances in the level of brain monoamine neurotransmitters such as serotonin, dopamine, and noradrenaline. Other possible etiological factors include alterations in glutamate receptors, neurogenesis, glial cells, BDNF, the neuroendocrine system, immune responses, stressful events, genetic factors, epigenetic modification, inflammatory mediators and the hypothalamic–pituitary–adrenocortical axis. However, a recent report indicated that only one-third of individuals with depression experience a complete therapeutic improvement using currently available antidepressant drugs designed based on the monoamine hypothesis, and a therapeutic effect appears only after several weeks of treatment (Nestler et al., 2002; Ye et al., 2011). Thus, the discovery of novel effective antidepressant medications is urgently needed.

Astrocytes are crucial to the neuronal microenvironment by regulating glucose metabolism, neurotransmitter uptake (particularly glutamate), synaptic development and maturation and the blood brain barrier, whose pathological damage is involved in many neurological diseases (Streit, 1995). Astrocytes synthesize and release many neurotrophic factors that play vital roles in neuronal survival, such as BDNF, glial-derived neurotrophic factor (GDNF), nerve growth factor (NGF), and neurotrophins 3,4 and 5 (Friedman et al., 1998; Althaus and Richter-Landsberg, 2000). These neurotrophic factors regulate neuronal growth, maintenance, and plasticity, and their decreased availability can lead to increased cellular vulnerability or even cell death. In mammals, astrocytes are able to take up serotonin by a sodium-dependent, high affinity system (Kimelberg and Katz, 1985), and they express several different 5-HT receptor subtypes, including 5-HT1A and 5-HT2A (Azmitia et al., 1996; Azmitia, 2001). Astrocytes also widely express stress hormone receptors, including mineralocorticoid and glucocorticoid receptors (MR and GR). Previous studies suggested that GR activation is involved in the inhibition of astrocyte proliferation (Yu et al., 2004; Unemura et al., 2012). Glial fibrillary acidic protein (GFAP) is the principal component of cytoskeletal intermediate filaments that is strongly expressed by mature and reactive astrocyte cells in the CNS (Jacque et al., 1978; Middeldorp and Hol, 2011).

A consistent observation of cell pathology in MDD has been reductions in glial cells, particularly in astrocytes (Rajkowska and Miguel-Hidalgo, 2007). Histopathological studies of *postmortem* brain tissue report reductions in the packing density or number of astrocytes in the dorsolateral prefrontal cortex, orbitofrontal cortex, subcortical cortex, anterior cingulate cortex and amygdala. The expression of GFAP, a marker protein of astrocytes, is also decreased in the frontal limbic cortex, hippocampal gray matter and thalamus. These studies suggest that the reduction in astrocytes in the cortical limbic circuit region of depression patients may be closely related to their depressive behavior. Functional abnormalities in astrocytes can result in the reduction of glutamate recycling and uptake and cause glutamate accumulation, which in turn leads to a reduction in BDNF and a decrease in hippocampal neurogenesis (Liu et al., 2017). Several studies have shown that the current clinical use of antidepressants such as FLX, riluzole and citalopram can also inhibit chronic mild stimulation-induced astrocyte reduction. Therefore, astrocytes have been suggested as a potential target for therapeutic interventions in MDD.

In recent years, the discovery of necroptosis has elicited significant interest in studying the implications of necroptosis in human diseases, including ischemic stroke, neurodegenerative disorders and rheumatoid arthritis (McQuade et al., 2013). When cells are deficient in activating the apical apoptotic inducer caspase-8, TNF-α binding to TNFR1 results in the activation of RIPK1. Activated RIPK1 undergoes autophosphorylation on multiple residues. RIPK3 is phosphorylated on Ser227 in necrosomes, and RIPK3 homo-oligomers eventually phosphorylate MLKL on the Thr357/Ser358 residues. Then, MLKL translocates from the cytoplasm to the cell membrane to disrupt cell membrane integrity, inducing necroptosis (Tian et al., 2019; Liu et al., 2020). The activation of RIPK1/RIPK3/MLKL causes necroptosis and promotes inflammation. It was recently reported that aluminum trichloride can cause hippocampal neural cell necroptosis and subsequent depression-like behavior in rats *via* the IL-1β/JNK signaling pathway (Tian et al., 2019). However, this study did not distinguish necroptosis occurring in hippocampal neurons or in astrocytes of depression-like behavior rats. To date, whether RIPK1/RIPK3/MLKL-induced necroptosis is involved in the reduction of astrocytes in MDD is still unknown. Therefore, we hypothesized that RIPK1/RIPK3/MLKL-induced necroptosis may be involved in the reduction of astrocytes in MDD.

Fluoxetine is the first serotonin-selective reuptake inhibitor (SSRI) used in the clinical treatment of major depressive disorder. Increasing evidence reveals that immune dysregulation and inflammation may play a vital role in the pathophysiology of depressive disorders. Previous studies reported that apart from the effects of fluoxetine on the monoaminergic neurotransmission system, it also exerts neuroprotective and anti-inflammatory effects (Nichols et al., 1990; Yu et al., 2004; Unemura et al., 2012; Chatterjee and Sikdar, 2013). Recent literature has reported that the antidepressant effect of fluoxetine is related to the reduction of astrocyte damage by activating autophagy. However, whether fluoxetine can inhibit RIPK1/RIPK3/MLKL-induced necroptosis of astrocytes in MDD is still unclear. We raised another hypothesis that fluoxetine may inhibit RIPK1/RIPK3/MLKL-induced necroptosis of astrocytes in MDD to protect astrocyte injury and to reduce proinflammatory cytokines (such as IL-6 and TNF-α) induced by depression disorder.

In this study, we established a CUMS model in mice and Cort-induced injury of human astrocytes to prove the above two scientific hypotheses.

## Methods and materials

### Animals

Male C57BL/6J mice (22 ± 3 g) were purchased from SLAC Laboratory Animals Co. Ltd. (Shanghai, China). All animal protocols were approved by the Animal Care and Use Committee of Soochow University (Use license: SYXK-2016-0050; Production license: SYXK-2017-0006). All mice were housed in standard laboratory conditions with a 12 h/12 h light/dark cycle and free access to food and water.

### Animal model and drug administration

Chronic unpredictable mild stress (CUMS), the most commonly used depression model, was established as previously described with minor modifications (Liu et al., 2017). Briefly, the mice were subjected to nine different stressors for 5 weeks. The stressors included food deprivation (20 h), water deprivation (18 h), wet cage (21 h), cage tilting (45°, 17 h), tail suspension (6 min), forced swimming (25°C, 5 min), continuous light (36 h), behavior restriction (2 h) and horizontal shaking (40 min). The mice were randomly exposed to one or two of these nine stressors each day, but the same stressors were not used for consecutive days (Supplementary Table S1). The mice were randomly divided into several groups, including the control group, the model group and the fluoxetine (FLX, MedChemExpress, HY-B0102A) group. Furthermore, to observe the antidepressant effect of FLX, the mice were intraperitoneally injected with FLX (10 mg/kg) daily. Body weight and behavioral tests were measured at 1, 3, and 5 weeks after CUMS treatment.

### Behavioral evaluation

Open field test (OFT). The OFT was designed to evaluate the spontaneous locomotor activity of animals. The open field apparatus consisted of a dark varnished wooden box (100 cm square chamber, 40 cm high walls). The mice were kept in the test room for 30 min to habituate to the new environment. Then, the mouse was singly placed in the center of the open field apparatus. Total distance traveled (mm) from 20 to 25 min and time(s) spent in the center area were counted using an automated behavioral tracking system (JLBehv-OFG-4, Shanghai Jiliang Software Technology). (Pesarico et al., 2016).

Forced swimming test (FST). Mice were subjected to the FST as described previously with minor modifications (Tao et al., 2016), and the experimenters were blinded to the treatment conditions. Briefly, the mice were individually placed in a glass cylinder (height 80 cm, diameter 30 cm) filled with 24°C–26°C, 50 cm deep water for 6 min. The total FST process took 6 min, and motionless time of the last 4 min was recorded; mice were considered immobile only when motionless or floating with only small necessary movements to keep the head above water.

Sucrose preference test (SPT). This test was performed as described previously with minor modifications (Wang et al., 2016). Before the sucrose preference test, mice were deprived of water and food for 24 h. Then, the mice were individually placed in the cage and given free excess to two bottles containing 1% (w/v) sucrose solution or water for 2 h. The consumed volumes of sucrose solution and water were measured after 2 h. The decreased preference for sucrose was calculated according to the following formula: sucrose preference (%) = (sucrose solution consumption)/(water consumption + sucrose solution consumption) ×100%.

Tail suspension test (TST). Mice were subjected to the TST as described previously (Wang et al., 2016), and the experimenters were blinded to the treatment conditions. TST represents a common symptom of depression (Ren et al., 2016). Briefly, the mice were individually suspended by their tails with adhesive tape (approximately 50 cm above the floor) in a sound-isolated room for 6 min. The total TST process took 6 min, and motionless time of the last 4 min was recorded; mice were considered immobile only when hanging passively and motionless.

### Cell culture, cell death injury model and cell death assay

#### Cell culture

Human astrocytes (Applied Biological Materials (ABM) Inc.) were cultured in Dulbecco’s modified Eagle’s medium (Gibco, 11995040) containing 10% fetal bovine serum (Gibco, 10099) and 1% penicillin/streptomycin (Beyotime, #C0222) at 37°C in a humidified atmosphere with 5% CO_2_/95% N_2_.

#### Corticosterone (Cort)-induced human astrocyte injury model *in vitro* and drug administration

Human astrocytes (HAs) were treated with 200 μM Cort for 1 h to induce cell injury*.* To observe the effects of necroptotic kinase inhibitors on the HA injury model, the cells were incubated with medium containing 100 μM Nec-1 (Selleck, S8037), 10 μM GSK872 (Millipore, 530389) or 1 μM NSA (Bio-Techne, 5025/10). HA was treated with 1 μM FLX during Cort treatment to estimate the protective function of FLX in the HA injury model.

#### Cell viability assay

Cell Counting Kit-8 (CCK-8, Dojindo, CK04) was used to evaluate the effect of different drugs on the viability of HA. HA was cultured in 96-well plates at a density of 5 × 10^3^ cells per well. After 24 h, cells were treated with different drugs for 1 h. Subsequently, HA was incubated with 10 μL CCK-8 solution for 2 h. The absorbance was measured at 450 nm using an Infinite^®^ M1000 PRO multimode microplate reader (Tecan Trading AG, Switzerland).

#### Propidium iodide staining

Propidium iodide (PI, Sigma–Aldrich, P4170) was used to analyze cell death. HA was cultured in 24-well plates at a density of 1 × 10^5^ cells per well. After 24 h, cells were treated with different drugs for 1 h. Subsequently, HA was incubated with PI (5 μg/ml) and Hoechst 33258 (1 μg/ml) for 20 min. FLX images were detected by inverted fluorescence microscopy (Olympus, CKX31).

### *In vitro* RIPK1 kinase activity assay

RIPK1 kinase activity was detected using the RIPK1 Kinase Enzyme System (Promega, VA7591). The RIPK1 kinase enzyme system included RIPK1 kinase (15 ng) and kinase assay buffer (200 mM Tris pH 7.5, 100 mM MgCl_2_, 50 mM NaCl, 0.5 mg/ml BSA, 0.02% CHAPS and 1 mM DTT). FLX or Nec-1 (10 μM) was coincubated with RIPK1 and kinase assay buffer. Reactions were supplemented with 50 μM ATP. The primary kinase reaction was carried out for 4 h at room temperature. Reactions were performed in 5 μL (5% final concentration of DMSO) and stopped by the addition of 5 μL of ADP-Glo reagent for 40 min at room temperature. A luminescent signal was generated by the addition of 10 μL of kinase detection reagent for 40 min at room temperature.

### Immunohistochemistry

Immunohistochemistry was performed as previously described (Qin et al., 2019). At 1 week, 3 or 5 weeks after CUMS treatment, the brains were harvested and fixed with 4% paraformaldehyde for 24 h, dehydrated by increasing concentrations of sucrose, embedded in OCT (Sakura, United States), and cut into 16-μm thick sections in the coronal plane. Subsequently, the brain slices were fixed with 4% paraformaldehyde for 10 min, permeabilized with 0.3% Triton X-100 for 30 min, and blocked with 1% BSA for 1 h at room temperature. The slices were incubated overnight with specific primary antibodies at 4°C and incubated with secondary antibodies (Supplementary Table S2) for 1 h at room temperature. Then, Hoechst 33258 (1:10000, Sigma–Aldrich, 23491-45-4) was used to incubate the sections for 15 min to stain the nuclei. Fluorescence images were obtained by confocal laser scanning microscopy (LSM 710, Carl Zeiss, Germany).

### Western blotting analysis

Western blotting analysis was performed as previously described (Xin et al., 2016). Cells and tissue were lysed with RIPA buffer, and the protein concentrations were quantitated by the BCA assay kit (Thermo Fisher Scientific, UB276926). Samples were electrophoresed on SDS–PAGE gels and transferred to PVDF membranes. The membranes were blocked with 5% skim milk and then incubated with specific primary antibodies (Supplementary Table S3) overnight at 4°C, followed by incubation with the corresponding secondary antibody (Supplementary Table S3) for 1 h at room temperature. The blots were detected by Odyssey scanner (LI-COR). β-actin was considered as the loading control.

### ELISA analysis

ELISA analysis was performed as previously described (Yan et al., 2021). The inflammatory cytokines TNF-α, IL-6 and IL-1β in brain tissue or cell culture medium were detected with ELISA kits (Elabscience Biotechnology, E-EL-M0037c for mouse IL-1β, E-EL-H0149c for human IL-1β, E-EL-M0049c for mouse TNF-α, E-EL-H0109c for human TNF-α, E-EL-M0044c for mouse IL-6, E-EL-H0102c for human IL-6). All procedures were performed according to the manufacturer’s instructions.

### Statistical analysis

ImageJ software was used to analyze image data. Statistical analysis for multiple comparisons was performed by one-way analysis of variance (ANOVA) followed by a *post hoc* Tukey’s test, and the difference between two groups was evaluated by unpaired Student’s *t* test. Multiple *t* tests with the *Bonferroni-Dunn method* and two-way ANOVA followed by a *post hoc* Tukey’s test were used for statistical analysis of behavior. All data were expressed as the mean ± SD. *p* < 0.05 was considered statistically significant.

## Results

### CUMS treatment induces depressive-like behaviors and reduced body weight gain, accompanied by a reduction in astrocytes, a decrease in BDNF, the activation of necroptotic kinases, and the upregulation of inflammatory cytokines in the mouse hippocampus in a time-dependent manner

**FIGURE 1:**
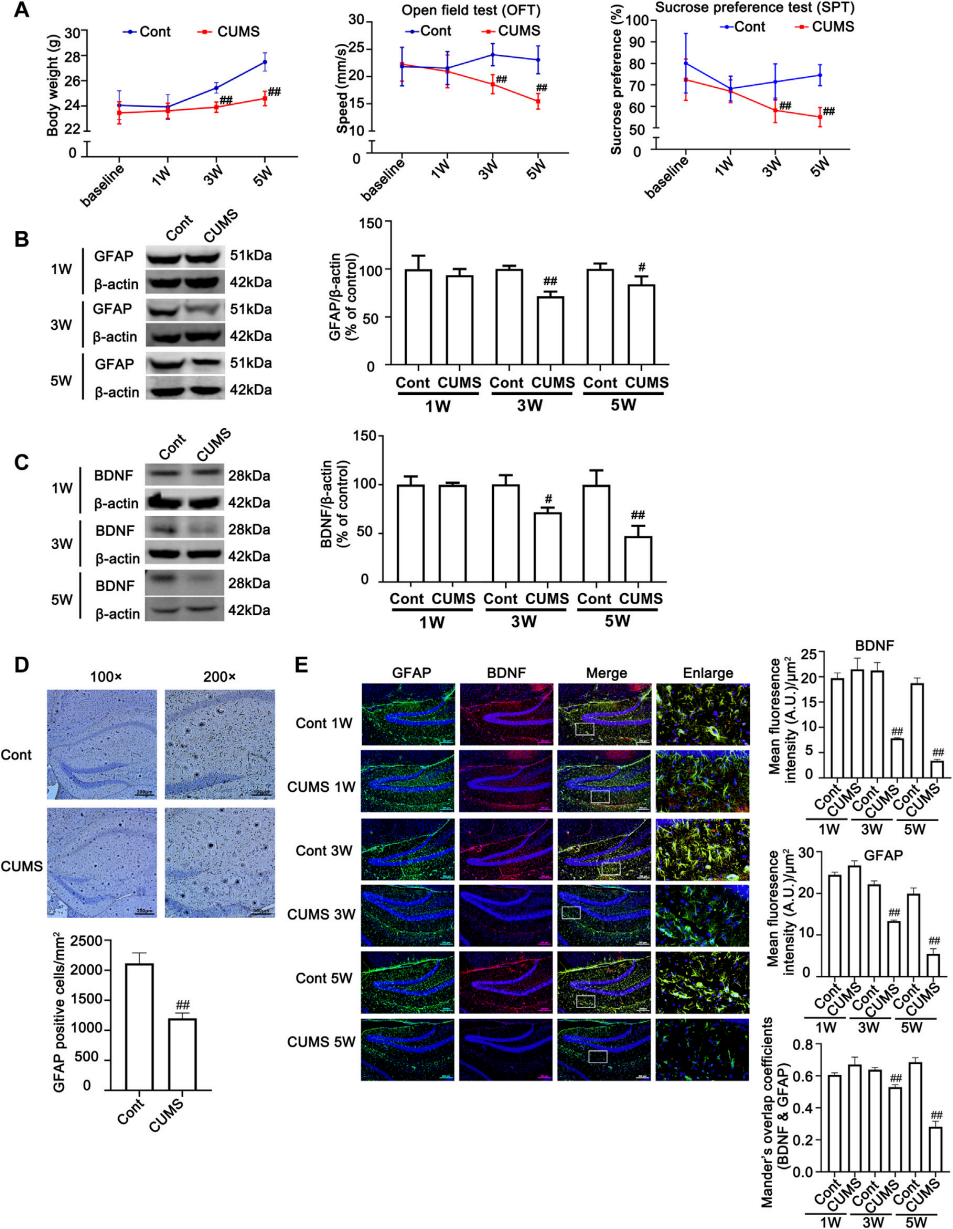
Chronic unpredictable mild stress (CUMS) treatment induces depressive-like behaviors and reduces body weight gain, accompanied by a reduction in astrocytes and a decrease in BDNF in the mouse hippocampus. Mice were treated with CUMS for 1, 3 and 5 weeks **(A)** Line chart of body weight, open field test and sucrose preference test in mice. The results are expressed as the mean ± *SD* (n = 8 per group). ##*p* < 0.01, vs. Cont. group. Statistical comparisons were carried out with *multiple t tests* with *the Bonferroni-Dunn method*. **(B and C)** Changes in GFAP and BDNF expression over different time courses after CUMS treatment, as determined by Western blot analysis. β-actin protein was used as a loading control. ImageJ software was used for immunoblot quantification, and statistics were performed by *Student’s t test*. Mean ± *SD*, *n* = 3 per group, #*p* < 0.05, ##*p* < 0.01 *vs.* Cont. group. **(D)** Representative images of immunochemistry in the hippocampus of Cont- or CUMS-treated mice. The astrocytes were stained with GFAP antibody (Brown). Statistical analysis was performed by *Student’s t test*. Mean ± *SD*, *n* = 6 per group, ##*p* < 0.01 vs. Cont. group. **(E)** Representative images of double immunofluorescence staining showing the expression of BDNF and GFAP in the hippocampus of Cont- or CUMS-treated mice at different time points. BDNF: red; GFAP: green; Hoechst: blue. Scale bars indicate 100 μm or 200 μm. Mander’s overlap coefficient represents the true degree of colocalization, its values range from 0 to 1.0, and zero means that there are no overlapping pixels. ImageJ was used to calculate the colocalization coefficients. Statistical analysis was performed by one-way *ANOVA* with *Tukey’s test* and expressed as the mean ± *SD*, *n* = 3 per group, ##*p* < 0.01 *vs.* Cont. group.

To verify the successful induction of the CUMS model in mice, body weight was determined, and behavioral tests, such as sucrose preference (SPT) and open field test (OFT), were performed before stress and every 2 weeks after CUMS treatment. As shown in Figure 1A, no significant difference was found in the body weight gain, spontaneous locomotor activity or preference for sugar water between the Cont group and CUMS group after 1 week of stress treatment, but the body weight gain dramatically declined, spontaneous locomotor activity markedly decreased and the preference for sugar water significantly decreased over time from 3 to 5 weeks in mice.

Previous studies have shown that hippocampal astrocytes play an extremely important role in depression, so we first detected the changes in hippocampal astrocytes in CUMS mice. Both Western blotting and immunohistochemistry results showed that the levels of GFAP in the hippocampus of mice showed no obvious changes after mice were treated with CUMS for 1 week, but it was significantly decreased after mice were treated with CUMS for 3 and 5 weeks (Figures 1B, E). In addition, the number of astrocytes in the hippocampus was significantly decreased in the mouse hippocampus after 5 weeks of treatment with CUMS (Figure 1D). Functional abnormalities in astrocytes can result in the reduction of glutamate recycling and uptake and cause glutamate accumulation, which in turn leads to a reduction in BDNF signaling and a decrease in hippocampal neurogenesis (Liu et al., 2017). The changes in the levels of BDNF are considered biomarkers for depression and play a vital role in the development of depression, and its restoration may underlie the therapeutic efficacy of antidepressant treatment. Bundles of previous clinical and animal studies have revealed that chronic stress induces a reduction in the level of depression biomarkers such as BDNF (Jacobsen et al., 2012). Therefore, we further detected the effect of CUMS on BDNF levels at different time points in mouse hippocampal tissue. The results revealed that the time course changes of BDNF levels and BDNF levels in GFAP positive cell in mouse hippocampus were similar to that of GFAP, presenting no significant changes of them after 1 week treatment and they were decreased after 3 weeks treatment, and were further decreased after 5 weeks treatment (Figures 1C, E). These results indicate that CUMS mediates the reduction in astrocytes and their dysfunction after mice are treated with CUMS for 3–5 weeks.

**FIGURE 2:**
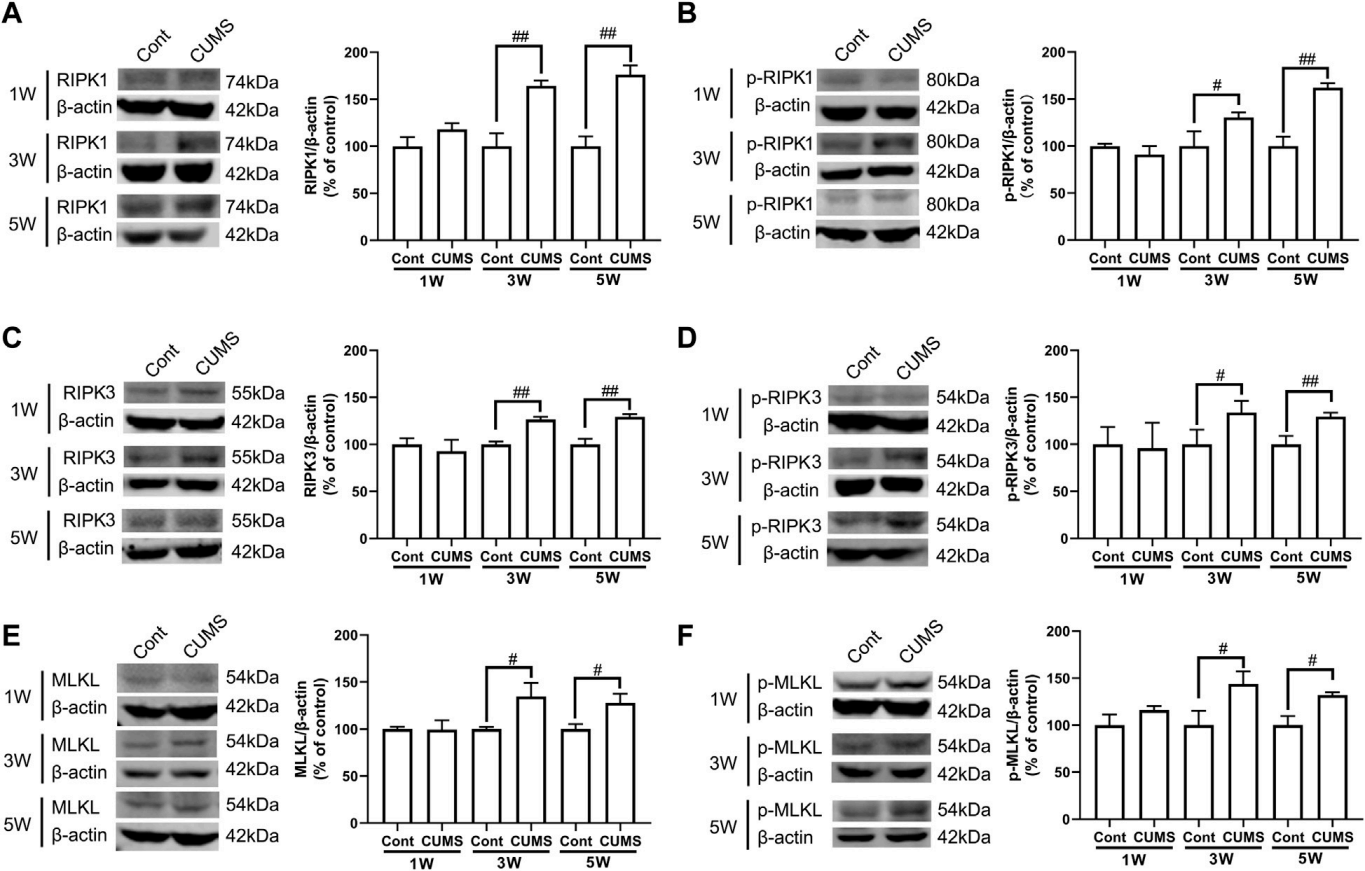
CUMS treatment induces the activation of necroptotic kinases in the mouse hippocampus in a time-dependent manner. Mice were treated with CUMS for 1, 3 and 5 weeks. The expression of RIPK1 **(A)**/p-RIPK1 **(B)**, RIPK3 **(C)**/p-RIPK3 **(D)**, and MLKL **(E)**/p-MLKL **(F)** in the hippocampus of Cont- or CUMS-treated mice over different time courses, as determined by Western blotting analysis. β-actin protein was used as a loading control. ImageJ software was used for immunoblotting quantification, and statistics were performed by *Student’s t test*. Mean ± *SD*, *n* = 3 per group, #*p* < 0.05, ##*p* < 0.01 *vs.* Cont. group.

**FIGURE 3:**
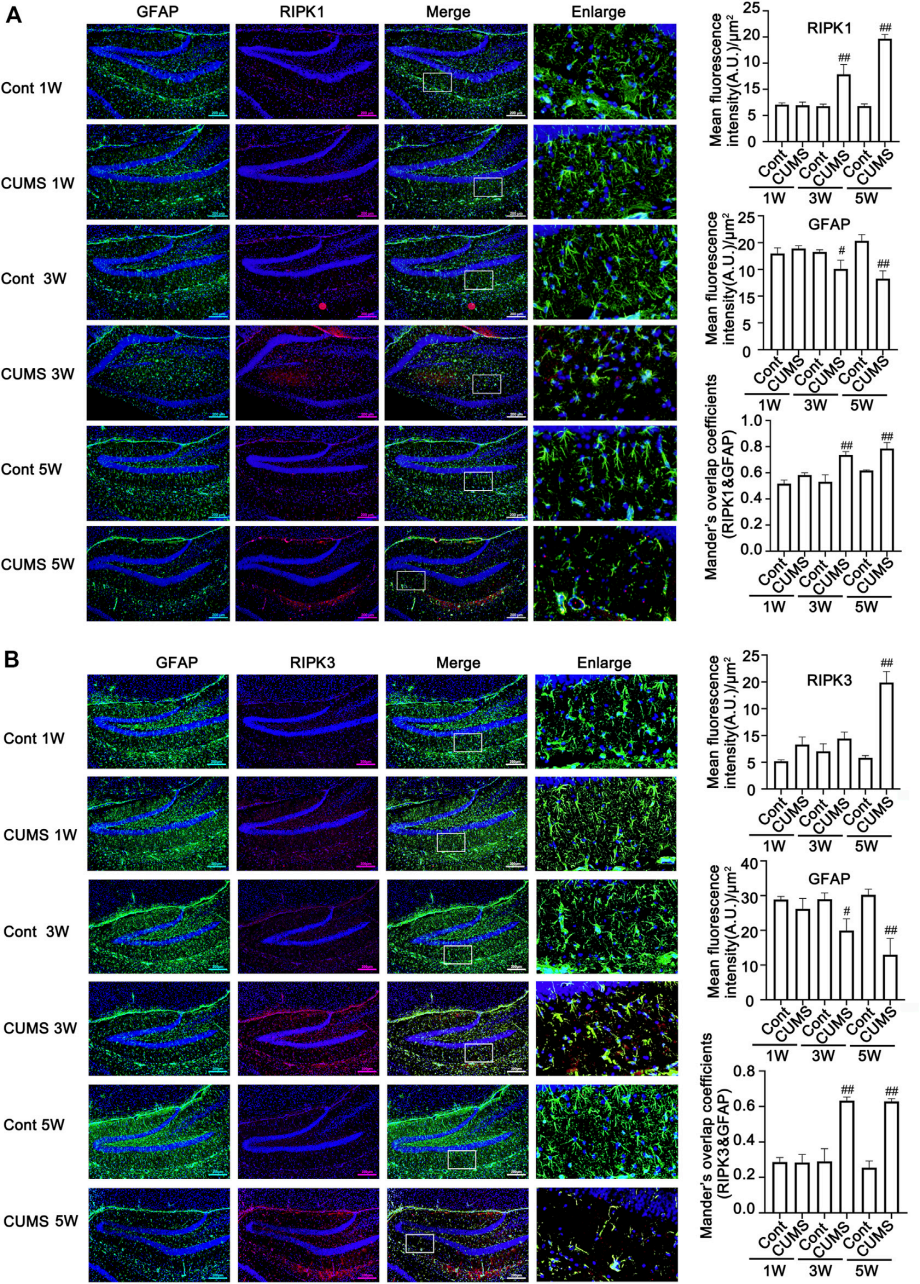
The expression of necroptotic kinases is increased in the hippocampal astrocytes of CUMS mice in a time-dependent manner. Representative images of double immunofluorescence staining showing the expression of RIPK1 **(A)** or RIPK3 **(B)** and GFAP in the hippocampus of Cont- or CUMS-treated mice at different time points. RIPK1 or RIPK3: red; GFAP: green; Hoechst: blue. Scale bars indicate 200 μm. Mander’s overlap coefficient represents the true degree of colocalization, its values range from 0 to 1.0, and zero means that there are no overlapping pixels. ImageJ was used to calculate the colocalization coefficients. Statistical results were performed by one-way *ANOVA* with *Tukey’s test* and expressed as the mean ± *SD*, *n* = 3 per group, #*p* < 0.05, ##*p* < 0.01 *vs.* Cont. group.

Next, we evaluated whether CUMS induces the activation of necroptotic kinases. The western blotting results showed that the necroptosis kinases RIPK1/p-RIPK1 (Figures 2A, B), RIPK3/p-RIPK3 (Figures 2C, D), and MLKL/p-MLKL (Figures 2E, F) were unchanged after 1 week of CUMS treatment, but the levels of these necroptotic kinases were increased and activated over time from 3 to 5 weeks in the hippocampus of CUMS mice. Similarly, immunohistochemistry results showed that in the hippocampal GFAP-positive astrocytes of CUMS mice, the expression of RIPK1 and RIPK3 was unchanged after 1 week of treatment and was increased over time from 3 to 5 weeks of treatment (Figure 3). These results indicate that CUMS induces RIPK1, RIPK3 and MLKL phosphorylation and activation in hippocampal astrocytes in a time-dependent manner.

Previous studies have shown that the activation of RIPK1/RIPK3/MLKL causes necroptosis and promotes inflammation. After mice were treated with CUMS for 1, 3 or 5 weeks, the concentrations of TNF-α, IL-1β and IL-6 in hippocampal tissue were measured by ELISA. The ELISA results showed that CUMS treatment for 1 week did not induce changes in the levels of TNF-α, IL-1β and IL-6 but mediated increases in the levels of TNF-α, IL-1β and IL-6 over time from 3 to 5 weeks in the mouse hippocampus (Supplementary Figure S1), indicating that CUMS treatment induces an increase in the levels of inflammatory cytokines in the mouse hippocampus in a time-dependent manner.

Necroptotic kinase inhibitors suppress corticosterone-induced necroptotic kinase activation, reduction in astrocytes, astrocytic necroptosis and dysfunction, and decrease in inflammatory cytokines.

Cort is a synthetic corticosteroid, and it has been reported that Cort can cause pathological damage to hippocampal neurons to induce depression-like behaviors in animals. The levels of Cort are elevated in blood of depression-like animals induced by CUMS (Sapolsky, 2000). Therefore, Cort is the most commonly used model to induce neuronal injury as an *in vitro* one for depression studies (Zheng et al., 2011). In this study, we used Cort to induce human astrocyte (HA) injury to mimic an *in vivo* model of depression. Nec-1, GSK872, or NSA is a specific inhibitor of RIPK1, RIPK3 or MLKL, which binds to the kinase domains of RIPK1, RIPK3 or MLKL with high affinity, respectively (Degterev et al., 2005; Gupta et al., 2018). We next used Nec-1, GSK872 and NSA as necroptotic kinase inhibitors to identify the role of necroptosis in Cort-induced HA injury.

**FIGURE 4:**
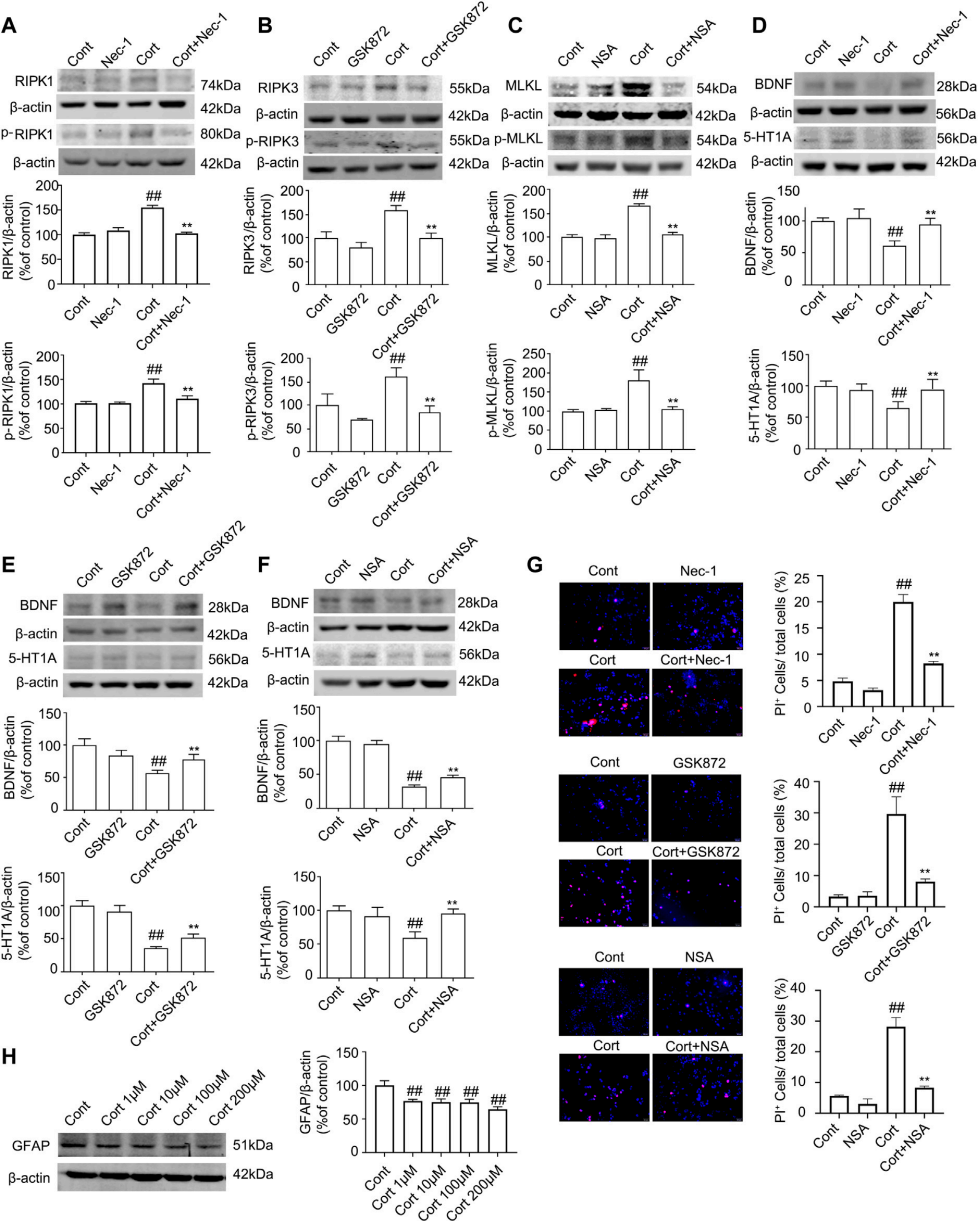
Necroptotic kinase inhibitors suppress corticosterone (Cort)-induced necroptotic kinase activation and protect Cort-treated astrocytic necroptosis in human astrocytes (HAs). HA was exposed to 200 μM Cort for 1 h to induce cell injury. HA was treated with 100 μM Nec-1, 10 μM GSK872 or 1 μM NSA during Cort treatment. **(A,B and C)** Representative Western blot images showing necroptotic kinase expression and activation in Cont- or Cort-treated HA. β-actin was used as a loading control. Data are mean ± *SD*, n = 3 per group, ##*p* < 0.01 *vs.* Cont. group, **p < 0.01 *vs.* Cort. group. Statistical analysis was carried out with one-way *ANOVA* followed by a *post hoc Tukey test*. **(D,E and F)** Representative Western blot images showing BNDF and 5-HT1A expression in Cont- or Cort-treated HA. β-actin was used as a loading control. Data are mean ± *SD*, *n* = 3 per group, ##*p* < 0.01 *vs.* Cont. group, **p < 0.01 *vs.* Cort. group. Statistical analysis was carried out with one-way *ANOVA* followed by a *post hoc Tukey test*. **(G)** Necrotic cells were assessed with propidium iodide (PI) and Hoechst staining (PI: red; Hoechst: blue). The Date are Mean ± *SD*, *n* = 3. ##*p* < 0.01 vs. Cont. group, **p < 0.01 *vs.* Cort. group. Statistical analysis was carried out with one-way *ANOVA* followed by a *post hoc Tukey test.* **(H)** Representative Western blot images showing GFAP expression in Cont- or Cort-treated HA. HA was treated with different concentrations of Cort (1, 10, 100, 200 μM) for 1 h to induce cell injury, and Western blotting was used to detect GFAP levels. β-actin was used as a loading control. Data are mean ± *SD*, *n* = 3 per group, ##*p* < 0.01 *vs.* Cont. group. Statistical analysis was carried out with one-way *ANOVA* followed by a *post hoc Tukey test.*

The Western blotting results showed that Cort at 1, 10, 100 and 200 μM significantly reduced the level of GFAP in HA after treatment with Cort for 1 h, and this reduction in GFAP was increased as the Cort concentration was increased (Figure 4H). Therefore, in the following experiments, HA cells were exposed to a high concentration of Cort (200 μM) to induce cell injury and cotreated with necroptotic kinase inhibitors (Nec-1, GSK872, and NSA). The Western blotting results showed that the protein levels of RIPK1/p-RIPK1, RIPK3/p-RIPK3 and MLKL/p-MLKL were increased in Cort-treated HA, indicating that Cort induces phosphorylated activation of necroptotic kinases (Figures 4A–C). In addition, 100 μM Nec-1, 10 μM GSK872 or 1 μM NSA significantly prevented the phosphorylated activation of RIPK1, RIPK3, and MLKL. Furthermore, the expression of BDNF and 5-HT1A was decreased in HA after 1 h of Cort treatment, and cotreatment with necroptotic kinase inhibitors Nec-1, GSK872 or NSA significantly upregulated BDNF and 5-HT1A expression levels (Figures 4D–F).

PI staining was used to detect Cort-induced HA necrosis. The proportion of PI-positive cells (necrotic cells) was significantly increased in the Cort-treated group compared with the control group. In contrast, Nec-1 (100 μM), GSK872 (10 μM) and NSA (1 μM) treatment successfully decreased the proportion of PI-positive cells (Figure 4G), indicating that Nec-1, GSK872 and NSA could inhibit Cort-induced astrocyte necroptosis by suppressing the phosphorylated activation of RIPK1, RIPK3, and MLKL.

To further identify the protective effects of necroptotic kinase inhibitors against Cort-induced HA cell injury, we next detected the release of inflammatory cytokines from HA using ELISA. The results showed that the levels of inflammatory cytokines, including TNF-α, IL-6 and IL-1β, were significantly increased in the Cort-treated HA cells compared to the control group (Supplementary Figure S2). In contrast, necroptotic kinase inhibitor treatment inhibited the level of inflammatory cytokines in Cort-induced HA. These data suggest that necroptotic kinase inhibitor treatment suppresses the Cort-induced release of inflammatory cytokines from HA.

### Fluoxetine improves CUMS-mediated depressive-like behaviors in mice and blocks the reduction of astrocytes and astrocyte necroptosis and dysfunction, as well as a decrease in inflammatory cytokines

FLX is a clinically approved drug used to treat major depressive disorder. It has been reported that fluoxetine has neuroprotective and anti-inflammatory effects in addition to acting on the monoaminergic neurotransmission system. We next evaluated whether FLX could suppress astrocyte necroptosis and reduce astrocytes by inhibiting the activation of necroptotic kinase.

**FIGURE 5:**
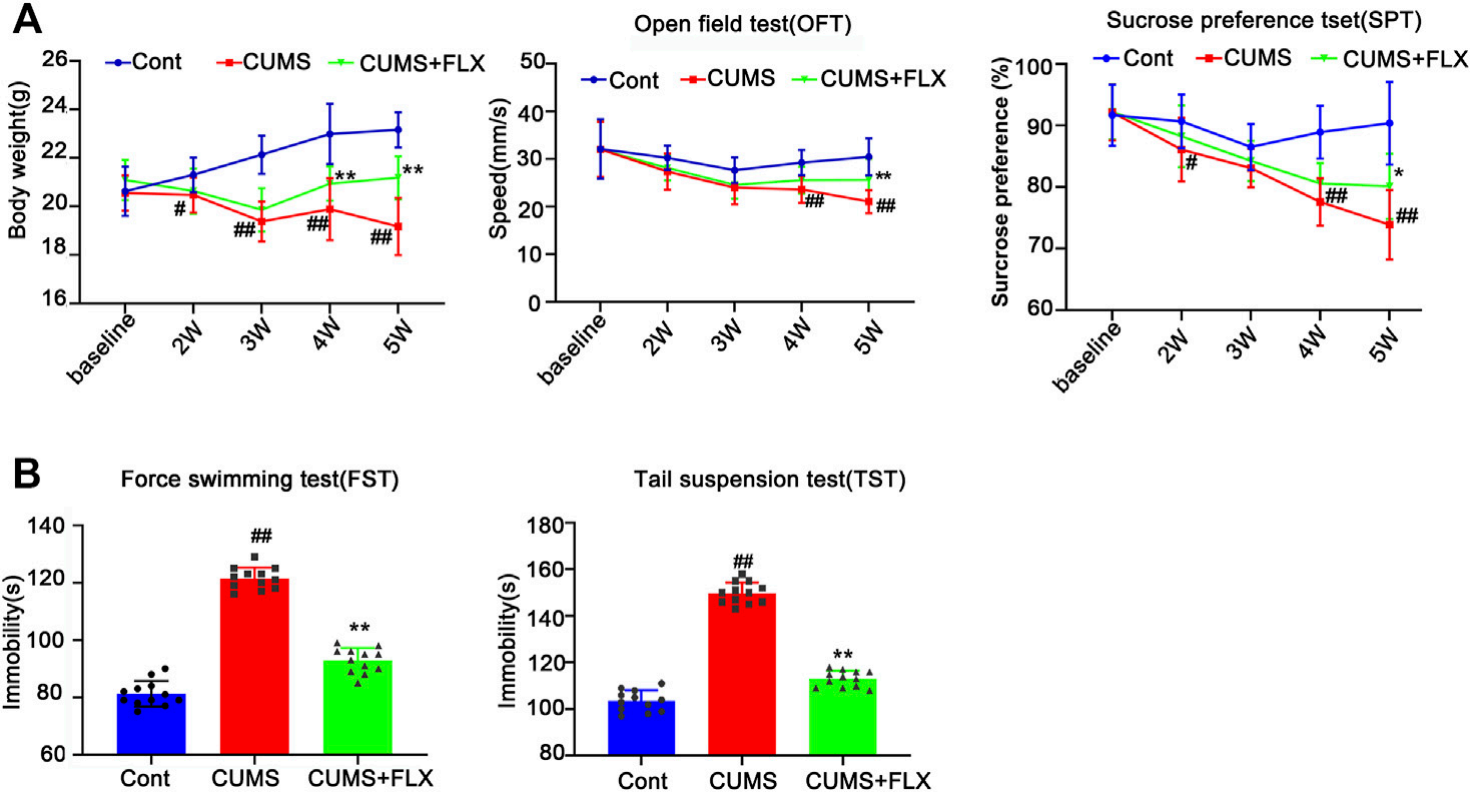
Fluoxetine (FLX) treatment improves CUMS-mediated depressive-like behaviors in mice. Mice were treated with CUMS for 5 weeks, and the body weight, open field test and sucrose preference test were performed before and after CUMS treatment for 2, 3, 4 and 5 weeks **(A)** The line chart graphically shows that body weight, open field test and sucrose preference test were changed at different weeks. The data are expressed as the mean *± SD* (*n* = 12 per group). *#p* < 0.05*, ##p* < 0.01, vs. Cont. group; **p < 0.01 vs. CUMS. Statistical analysis was carried out with two-way *ANOVA* followed by a *post hoc Tukey test*. **(B)** FLX treatment decreased the immobility time of CUMS mice in the force swimming test and tail suspension test. The force swimming test and tail suspension test were performed at week 5 after CUMS treatment. The results are expressed as the mean ± *SD* (*n* = 12 per group). ##*p* < 0.01, *vs.* Cont. group, **p < 0.01 vs. CUMS. group. Statistical analysis was carried out with one-way *ANOVA* followed by the *Tukey test.*

**FIGURE 6:**
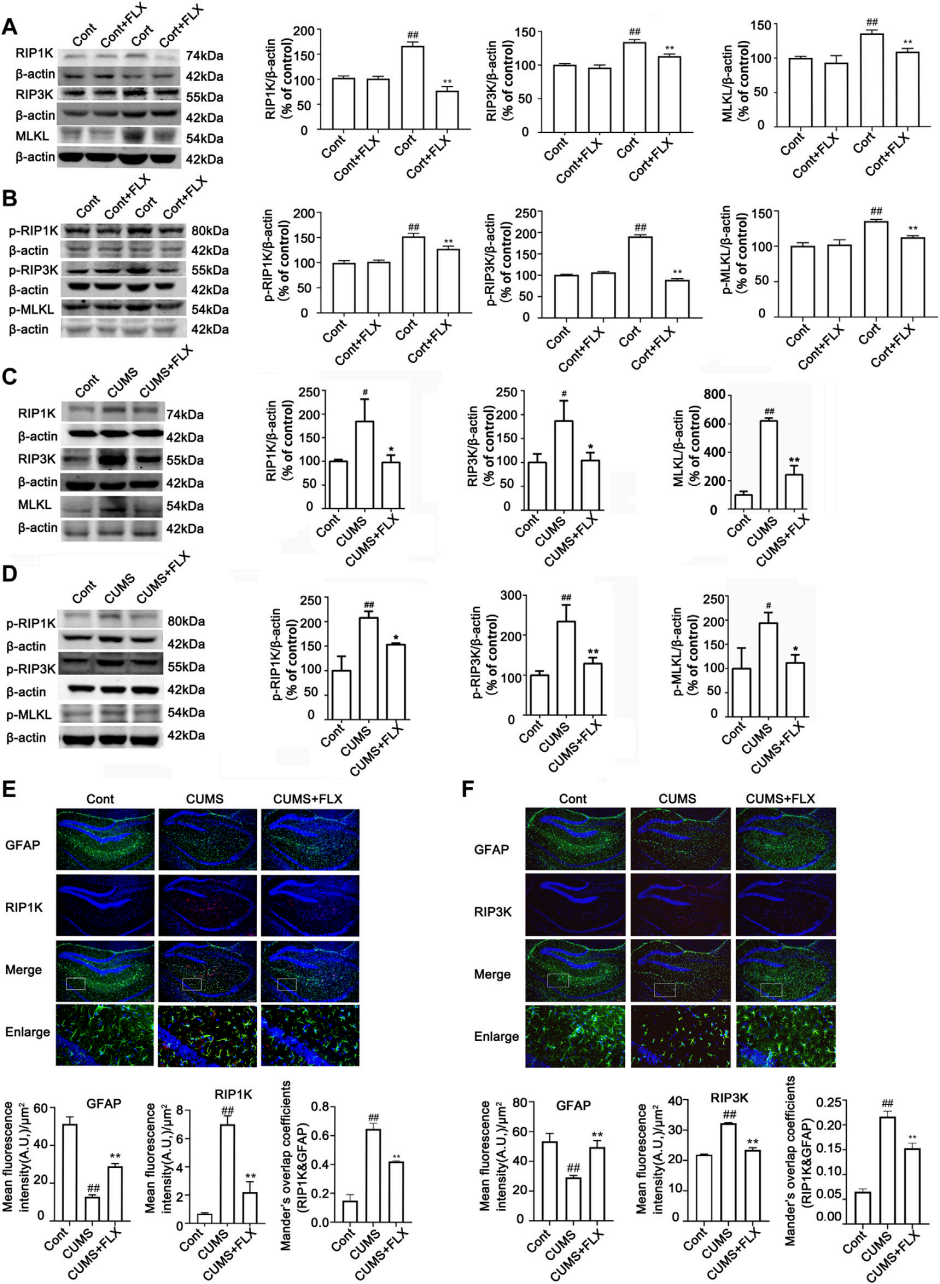
FLX treatment inhibits CUMS- or Cort-induced activation of necroptotic kinases of astrocytes in the hippocampus of mice or in HA cells. HA was exposed to 200 μM Cort for 1 h. HA was treated with 1 μM FLX during Cort treatment. The mice were treated with CUMS for 5 weeks, and FLX (10 mg/kg) was administered intraperitoneally every day during CUMS treatment. **(A and B)** Representative Western blotting images showing the expression of RIPK1/RIPK3/MLKL and p-RIPK1/p-RIPK3/p-MLKL in Cont- or Cort-treated HA. β-actin was used as a loading control. ImageJ software was used for immunoblotting quantification, and statistics were performed by one-way *ANOVA* with *Tukey’s test*. Mean ± *SD*, n = 3 per group, #*p* < 0.05, ##*p* < 0.01 *vs.* Cont. group, *p < 0.05, **p < 0.01 vs. Cort. group. **(C and D)** Representative Western blotting images showing the expression of necroptotic kinases RIPK1/RIPK3/MLKL and p-RIPK1/p-RIPK3/p-MLKL in the hippocampi of Cont- or CUMS-treated mice. β-actin was used as a loading control. ImageJ software was used for immunoblotting quantification, and statistical analysis was performed by one-way *ANOVA* with *Tukey’s test*. Mean *± SD, n* = 3 per group, *#p* < 0.05*, ##p* < 0.01 vs. Cont. group, *p < 0.05, **p < 0.01 vs. CUMS. group. **(E and F)** Representative images of double immunofluorescence staining showing the expression of RIPK1, RIPK3 and GFAP in the hippocampus of Cont- or CUMS-treated mice. GFAP: green; RIPK1 or RIPK3: red; Hoechst: blue. Scale bar indicates 200 μm. Columns represent quantitative analysis of GFAP and RIPK1 or RIPK3. Statistical analysis was performed by one-way *ANOVA* with *Tukey’s test*. The data are expressed as the mean *± SD, n* = 3 per group, *##p <* 0.01 *vs.* Cont. group. **p < 0.01 *vs.* CUMS. group.

**FIGURE 7:**
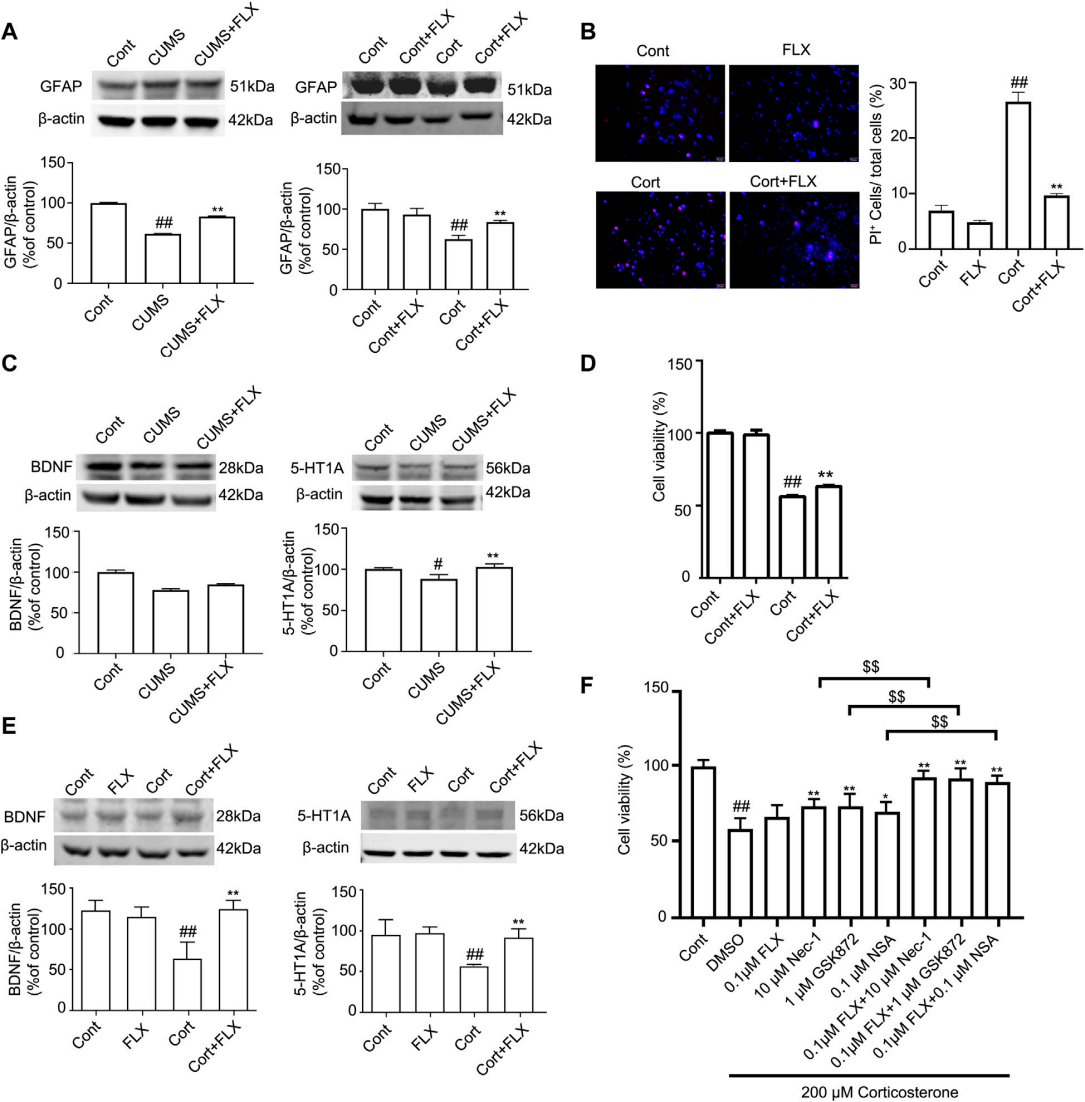
FLX treatment prevents depression-induced reduction of astrocytes and increases the level of GFAP protein *in vivo and in vitro.* HA was exposed to 200 μM Cort for 1 h. HA was treated with 1 μM FLX during Cort treatment. The mice were treated with CUMS for 5 weeks, and FLX (10 mg/kg) was administered intraperitoneally every day during CUMS treatment. **(A)** Representative Western blotting images of GFAP in the mouse hippocampus and HA cells. β-actin protein was used as a loading control. Data are mean ± *SD*, *n* = 3. ##*p* < 0.01 vs. Cont. group, **p < 0.01 vs. CUMS. group or Cort. group. Statistical analysis was carried out with one-way *ANOVA* followed by *Tukey’s test*. **(B)** HA necrosis was assessed with propidium iodide (PI) and Hoechst staining (PI: red; Hoechst: blue). Mean ± *SD*, *n* = 3. ##*p* < 0.01 *vs.* Cont. group, **p < 0.01 vs. Cort. group. Statistical analysis was carried out with one-way *ANOVA* followed by *Tukey’s test* **(C and E)** Representative Western blotting images of BDNF and 5-HT1A in the mouse hippocampus and HA cells. β-actin was used as a loading control. Mean ±* SD*, *n* = 3. #*p* < 0.05, ##*p* < 0.01 vs. Cont. group, **p < 0.01 *vs.* CUMS. group or Cort. group. Statistical analysis was carried out with one-way *ANOVA* followed by *Tukey’s test.* **(D)** CCK-8 assays were used to measure cell viability. Mean ± *SD*, *n* = 6. ##*p* < 0.01 *vs.* Cont. group, **p < 0.01 vs. Cort. group. Statistical analysis was carried out with one-way *ANOVA* followed by *Tukey’s test.* **(F)** The combined administration of low-dose FLX and necroptotic kinase inhibitors further increased cell viability in Cort-treated HA. HA cells were exposed to 200 μM Cort for 1 h. HA cells were treated with 0.1 μM FLX, 10 μM Nec1, 1 μM GSK872 and 0.1 μM NSA alone or 0.1 μM FLX cotreated with 10 μM Nec1, 1 μM GSK-872 or 0.1 μM NSA during Cort treatment. CCK-8 assay was used to measure the cell viability. Mean ± *SD*, *n* = 6. ##*p* < 0.01 *vs.* Cont. group, *p < 0.05, **p < 0.01 vs. Cort. group. $$ *p* < 0.01 *vs.* Nec-1 or GSK-872 or NSA groups. Statistical comparisons were carried out with one-way *ANOVA* followed by *Tukey’s test.*

FLX (10 mg/kg) was administered intraperitoneally each day during CUMS treatment in mice for 5 weeks. Treatment with FLX for 4 or 5 weeks improved the body weight gain of CUMS mice, increased the locomotor activity of depressed mice in the OFT and elevated the percentage of sucrose consumed by mice subjected to CUMS (Figure 5). In addition, treatment with FLX for 5 weeks suppressed the CUMS-mediated reduction in GFAP levels (Figure 7A), downregulated the levels of RIPK1/p-RIPK1, RIPK3/p-RIPK3 and MLKL/p-MLKL (Figures 6C, D), reversed the CUMS-induced reduction in the levels of 5-HT1A and BDNF (Figure 7C), inhibited CUMS-mediated inflammatory cytokine release (Supplementary Figure S3A), and downregulated the immunostaining of RIPK1 or RIPK3 in GFAP-positive astrocytes in the hippocampus of mice (Figures 6E, F). Similarly, FLX treatment inhibited the levels of necroptotic kinases, including RIPK1/p-RIPK1, RIPK3/p-RIPK3 MLKL/p-MLKL, in the Cort-induced human astrocyte injury model (Figures 6A, B), reversed the Cort-induced reduction in cell viability (Figure 7D) and the Cort-induced increase in the proportion of propidium iodide (PI)-positive cells (Figure 7B), increased the protein levels of GFAP (Figure 7A), BDNF and 5-HT1A (Figure 7E), and decreased the Cort-induced release of inflammatory cytokines from human astrocytes (Supplementary Figure S3B).

### FLX has no direct inhibitory effect on RIPK1 phosphorylation

We next detected whether FLX has a direct inhibitory effect on RIPK1 phosphorylation using an *in vitro* ADP-Glo kinase assay. The results showed that the positive control Nec-1 could directly inhibit RIPK1 phosphorylation, whereas FLX had no direct inhibitory effect on RIPK1 phosphorylation (Supplementary Figure S4).

### The combined administration of fluoxetine and necroptotic kinase inhibitors further reduced Cort-induced astrocyte injury

The CCK-8 assay showed that in Cort-treated HA cells, a low dose of fluoxetine alone had no effects on cell viability, and a low dose of Nec-1, GSK872 or NSA alone slightly increased cell viability. However, the combined administration of a low dose of fluoxetine and a low dose of Nec-1, GSK872 or NSA further increased cell viability (Figure 7F). These data indicate that the combination of fluoxetine with necroptotic kinase inhibitors might produce synergistic pharmacological effects in protecting against depression-induced astrocyte injury.

## Discussion

In the present study, first, our results showed that CUMS treatment for 3–5 weeks induced depression-like behavior and body weight loss, accompanied by a series of biochemical changes in the mouse hippocampus presenting a reduced number of astrocytes, decreased protein levels of BDNF, activated necroptosis kinases, and upregulated inflammatory cytokines. Second, our results indicated that Cort-induced astrocyte necroptosis may contribute to the reduction in astrocytes, reflected by necroptosis kinase inhibitors decreasing the necrosis rate of astrocytes and necroptosis-related protein levels, including RIPK1/p-RIPK1, RIPK3/p-RIPK3, and MLKL/p-MLKL, upregulating the protein levels of BDNF and 5-HT1A in astrocytes and decreasing the levels of inflammatory cytokines in astrocytes. Third, our data suggested that FLX treatment improves CUMS-mediated depressive-like behaviors in mice, and its mechanism is associated with FLX decreasing necroptosis-related protein levels, such as RIPK1/p-RIPK1, RIPK3/p-RIPK3, and MLKL/p-MLKL, inhibiting astrocyte necrosis, upregulating the protein levels of BDNF and 5-HT1A in astrocytes and decreasing the levels of inflammatory cytokines in astrocytes. Fourth, we found that FLX has no direct inhibitory effect on RIPK1 phosphorylation. Fifth, our data revealed that the combination of FLX and necroptosis inhibitors further reduced the Cort-induced necrosis of astrocytes.

Cellular and molecular abnormalities arising from genetic and environmental factors play a critical role in the pathology of depression (Krishnan and Nestler, 2008). It is believed that reductions in astrocytes are key features in the pathology of major depressive disorder (MDD). Histopathological studies of *postmortem* brain tissue revealed reductions in the packing density or number of general Nissl-stained populations of glial cells in fronto-limbic brain regions, including the dorsolateral prefrontal cortex (Rajkowska et al., 1999; Cotter et al., 2002), orbitofrontal cortex (Rajkowska et al., 1999), subgenual cortex (Ongur et al., 1998), anterior cingulate cortex (Cotter et al., 2001; Gittins and Harrison, 2011) and amygdala (Bowley et al., 2002), in subjects diagnosed with MDD compared to non-psychiatric control subjects (Ongur et al., 1998; Rajkowska et al., 1999; Cotter et al., 2001; Bowley et al., 2002; Cotter et al., 2002; Gittins and Harrison, 2011). Decreases in the density and area fraction of GFAP-immunoreactive astrocytes and in the levels of GFAP protein or GFAP mRNA were also found in fronto-limbic cortical regions, in the CA1 and CA2 subregions of the hippocampus, as well as in subcortical brain regions including locus coeruleus, limbic thalamic nuclei, putamen and the internal capsule from subjects with MDD, revealing dysfunctional astrocytes in MDD. Similarly, preclinical studies further provide evidence that various types of stress cause reductions in measures of GFAP-immunoreactive astrocytes and in the levels of GFAP proteins or GFAP mRNA in cortical and hippocampal regions in animal models of depressive-like behavior. Consistent with these previous observations, the current study confirmed that CUMS treatment significantly reduced the number of GFAP-positive cells and the protein levels of GFAP in the hippocampus of CUMS-treated mice over time from 3 to 5 weeks, while consecutive treatment with FLX for 5 weeks markedly increased the level of GFAP in the hippocampus of CUMS mice and improved depression-like behaviors. Additionally, in Cort-treated HA, the protein level of GFAP was decreased, whereas FLX treatment increased it. CCK-8 results showed that FLX treatment reversed the Cort-induced reduction in HA viability compared to the control group. Furthermore, the levels of BDNF in the mouse hippocampus were markedly decreased over time from 3 to 5 weeks; in contrast, FLX treatment increased CUMS- or Cort-induced reduced levels of BDNF and 5-HT1A in the mouse hippocampus and in human astrocytes *in vitro*. Among the most potent factors known to trigger major depressive episodes are stressful life events (Xie et al., 2015). Psychological trauma or chronic stress is recognized as the critical causal or exacerbating factor of depression (Kessler et al., 2005). Upon exposure to strong and long-term physical and psychological stress stimuli, neuroendocrine activity is elevated, thus resulting in hypothalamic–pituitary–adrenocortical axis hyperactivity and increased glucocorticoid levels. Cortisol is believed to become an effective biomarker of stress-related and psychiatric disorders (Vahia, 2013). Emerging evidence has revealed that cortisol levels are elevated in patients with depression, thereby leading to cognitive dysfunction and mood disorders through hippocampal atrophy (Trivedi et al., 2006; Krishnan and Nestler, 2010). In patients with severe depression, bilateral hippocampal volume is remarkably reduced (Krishnan and Nestler, 2008). In rodents, long-term subcutaneous Cort injection causes hippocampal atrophy and induces depression-like behaviors (Volterra and Meldolesi, 2005; Rajkowska and Miguel-Hidalgo, 2007). Emerging evidence suggests that the CUMS model of depression can induce depressive-like behaviors, peripheral and central inflammation, neuronal cell damage, hyperactivity of the hypothalamic–pituitary–adrenal axis (HPA), increased hippocampal apoptosis, decreased neurogenesis, and reduced BDNF and 5-HT1A levels (Czeh and Di Benedetto, 2013; Yirmiya et al., 2015). Astrocytes widely express glucocorticoid receptors (GRs). Previous studies suggested that glucocorticoid receptor activation is involved in the inhibition of astrocyte proliferation (Yu et al., 2004; Unemura et al., 2012). In addition, astrocytes are able to synthesize and release BDNF, one of the major neurotrophic factors, regulating the survival, differentiation and outgrowth of peripheral and central neurons during development and in adulthood. Reduced BDNF protein were detected in the hippocampus both in *postmortem* brain samples from psychiatric disorder patients who had committed suicide, and in depressed rats (Zhang et al., 2016). Furthermore, astrocytes express several different 5-HT receptor subtypes, including 5-HT1A and 5-HT2A (Azmitia et al., 1996; Azmitia, 2001). In particular, the activation of 5-HT1A receptors is believed to be a crucial mechanism of action of SSRIs (Santarelli et al., 2003), occurring in high abundance on hippocampal astroglia (Azmitia et al., 1996). Therefore, reduced astrocytes in the limbic regions of the brain and decreased function of astrocytes, such as reduced levels of BDNF and 5-HT1A observed by others and by us, may be associated with glucocorticoid overproduction induced by stress (Chatterjee and Sikdar, 2013), and FLX treatment improves CUMS**-**induced depression-like behaviors, which may be associated with an increase in the number of astrocytes and with an enhancement in the function of astrocytes reflected by increased levels of BDNF and 5-HT1A.

Necroptosis is a non-caspase-dependent programmed cell death and is considered an injury process. Necroptosis presents some morphological characteristics of necrosis, except that it can be regulated by a variety of molecules (Vanden Berghe et al., 2010), and the formation of RIPK1, RIPK3 and MLKL complexes is the core initiation step for necroptosis (Li et al., 2012). Activated RIPK3 binds to and activates MLKL, and then MLKL translocates from the cytoplasm to the cell membrane, resulting in necroptosis (Sun et al., 2012; Dondelinger et al., 2014). To our knowledge, this study is the first to examine whether RIPK1/RIPK3/MLKL-induced necroptosis is involved in the reduction of astrocytes under long-term psychosocial stress and whether antidepressant fluoxetine treatment can inhibit RIPK1/RIPK3/MLKL-induced necroptosis of astrocytes. It was recently reported that aluminum trichloride can induce hippocampal neural cell necroptosis and subsequent depression-like behaviors in rats *via* the IL-1β/JNK signaling pathway (Tian et al., 2019). However, that study did not distinguish necroptosis occurring in hippocampal neurons or in astrocytes of depression-like behavior mice. In the current study, we found a reduced number of astrocytes and elevated levels of necroptosis kinases, including RIPK1/p-RIPK1, RIPK3/p-RIPK3, and MLKL/p-MLKL, in hippocampal astrocytes of depression-like behavior mice. Furthermore, we found that necroptosis kinase inhibitors decreased the Cort-induced increase in the necrosis rate of astrocytes and necroptosis-related protein levels, such as RIPK1/p-RIPK1, RIPK3/p-RIPK3, and MLKL/p-MLKL. These data indicate that stress-induced astrocyte necroptosis may contribute to the reduction in astrocytes in depression-like behavior mice. It is thought that antidepressants mediate their therapeutic effects by acting on neurons, especially monoaminergic neurons, but they also act on non-neuronal cells such as glial cells. However, to date, whether FLX can inhibit RIPK1/RIPK3/MLKL-induced necroptosis of astrocytes in MDD is still unclear. In the present study, we revealed that FLX treatment for 5 weeks improved depression-like behavior in mice, decreased necroptosis-related protein levels, such as RIPK1/p-RIPK1, RIPK3/p-RIPK3, and MLKL/p-MLKL, and inhibited astrocyte necrosis, suggesting that FLX-mediated antidepressive effects may be partially associated with inhibiting RIPK1/RIPK3/MLKL-induced necroptosis of astrocytes.

The activation of RIPK1/RIPK3/MLKL causes necroptosis and promotes inflammation. In a variety of animal disease models, RIPK1/RIPK3/MLKL inhibition has a certain anti-inflammatory effect (Newton and Manning, 2016). The RIPK1 inhibitor Nec-1 and MLKL inhibitor NSA effectively reduced imiquimod-induced inflammatory effects in mice and significantly downregulated the production of inflammatory factors such as IL-1β, IL-6, IL-17A, IL-23a, CXCL1, and CCL20. Necroptosis signaling is initiated by RIPK1 and requires RIPK3 and MLKL (Duan et al., 2020). The activation of RIPK1-mediated necroptosis significantly promotes neuroinflammation because it promotes the cascade expression of proinflammatory genes. RIPK1 acts as a scaffold, especially in the process of tumor necrosis factor-mediated activation of the NF-κB and JNK pathways to produce cytokines (Barabino et al., 2016; Dhuriya and Sharma, 2018). However, RIPK1 also activates and binds to EDD, mediates the JNK signaling pathway to promote the transcription of TNF-α, and promotes inflammation; this pathway is independent of NF-κB (Christofferson et al., 2012). The main pathway of inflammation after RIPK3 activates MLKL is the release of DAMPs (danger-associated molecular patterns) from cells. RIPK3 also directly activates the formation of inflammasomes, activating caspase-1 and caspase-11, and caspase-1 cleaves IL-1β into mature forms. This activation occurs through two different RIPK3-dependent pathways: one is mediated by caspase-8, and the other is mediated by NLPR 3 (NOD, LRR and pyrin) (Vince et al., 2012; Man et al., 2013). In addition, RIPK3 recruits complexes containing RIPK1, FADD and caspase-8 to promote inflammation (Lawlor et al., 2015; Moriwaki et al., 2015; Conos et al., 2017). Previous studies reported that apart from its effects on the monoaminergic neurotransmission system, FLX also exerts neuroprotective and anti-inflammatory effects (Nichols et al., 1990; Yu et al., 2004; Unemura et al., 2012; Chatterjee and Sikdar, 2013). FLX may reduce inflammatory cytokines (such as TNF-α, IL-1β, and IL-6) by inhibiting the phosphorylation and nuclear translocation of the p65 subunit of NF-κB and the phosphorylation of p38 mitogen-activated protein kinase (MAPK) in LPS-stimulated glial cells (Liu et al., 2011; Wang et al., 2019; Zhao et al., 2020).

In this study, we discovered a new mechanism by which FLX inhibits inflammation. We found that FLX can reduce inflammatory cytokines by indirectly inhibiting RIPK1/RIPK3/MLKL-mediated necroptosis in hippocampal astrocytes of mice. In addition, we also found that the combination of FLX and necroptosis inhibitors (Nec-1, GSK872 or NSA) could produce synergistic pharmacological effects in reducing Cort-induced cell death, which could provide a new strategy for the clinical treatment of depression. Interestingly, we found that FLX could not directly inhibit RIPK1 phosphorylation. How FLX can indirectly suppress RIPK1/RIPK3/MLKL signaling activation remains to be investigated. A recent study demonstrated that fluoxetine binds to TRKB and allosterically increases BDNF signaling (Casarotto et al., 2021), thereby directly linking the effects of antidepressant drugs to neuronal plasticity. Enhancing BDNF/TRkB signaling could activate the PI3K/Akt pathway (Tejeda and Diaz-Guerra, 2017; Wang et al., 2019). Activation of the PI3K/Akt pathway promotes the activation of p38, which suppresses the phosphorylation of Ser321/320 of cytosolic RIPK1 (Jaco et al., 2017; Wang et al., 2019). In addition, the activation of the PI3K/Akt pathway inhibits GSK3β, leading to a reduction in necroptosis (Tejeda and Diaz-Guerra, 2017; Liu et al., 2020). Therefore, we speculate that the inhibition of RIPK1/RIPK3/MLKL-induced necroptosis by fluoxetine may be related to its direct binding to TRKB.

## Conclusion

1) CUMS or Cort mediates RIPK1/RIPK3/MLKL activation in astrocytes and astrocyte necroptosis, contributing to the reduction and dysfunction of astrocytes and the release of inflammatory cytokines in depression-like mouse model or Cort-induced HA injury.
2) Necroptotic inhibitors such as Nec-1, GSK872 and NSA suppress astrocyte necroptosis by inhibiting RIPK1/RIPK3/MLKL activation, resulting in protection against Cort-induced astrocyte injury and reduced release of inflammatory cytokines.
3) Fluoxetine exerts an antidepressive effect by indirectly inhibiting RIPK1/RIPK3/MLKL-mediated astrocytic necroptosis, leading to protection against CUMS- or Cort-induced astrocyte injury, enhancing astrocyte function, and reducing the release of inflammatory cytokines.
4) Fluoxetine and necroptosis inhibitors (Nec-1, GSK872 or NSA) produce synergistic pharmacological effects in protecting against Cort-induced astrocyte injury.

However, there are still some limitations to this study, including the fact that in CUMS mice model, we did not detect the necroptotic astrocytes directly, and also did not clarify the effect of necroptosis inhibitors Nec-1, GSK872 or NSA. These issues remains to be investigate in the future.

## Data Availability

The original contributions presented in the study are included in the article/Supplementary Materials, further inquiries can be directed to the corresponding authors.

## Abbreviations

FLX, Fluoxetine; FADD, Fas-Associated Protein With Death Domain; FST, Force Swimming Test; GFAP, Glial Fibrillary Acidic Protein; GDNF, Glial-Derived Neurotropic Factor; GR, Glucocorticoids Receptors; HPA, Hypothalamic-Pituitary-Adrenal Axis; IL-6, Interleukin 6; IL-1β, Interleukin 1 Beta; MDD, Major Depressive Order; MLKL, Mixed Lineage Kinase Like Protein; MAOIS, Mono Amine Oxidase Inhibiter; MR, Mineralocorticoid Receptors; OFT, Open Field Test; RIPK1, Receptor Interacting Protein 1 Kinase; RIPK3, Receptor Interacting Protein 3 Kinase; SSRI, Selective Serotonin Reuptake Inhibitor; SPT, Sucrose Preference Test; TNF-α, Tumor Necrosis Factor; TRADD, TNF-Receptor-Associated Death Domain; TST, Tail Suspension Test; TCAS, Tricyclic Antidepressants; 5-HT1A, 5-Hydroxytryptamine or Serotonin.
