# Supplementary material for: Necroptotic kinases are involved in the reduction of depression-induced astrocytes and fluoxetine’s inhibitory effects on necroptotic kinases
Source: Front Pharmacol. 2023 Jan 4;13:1060954. doi: 10.3389/fphar.2022.1060954 (PMC9847570; doi:10.3389/fphar.2022.1060954)
Supplement: Supplementary file 1 [file DataSheet1.pdf]

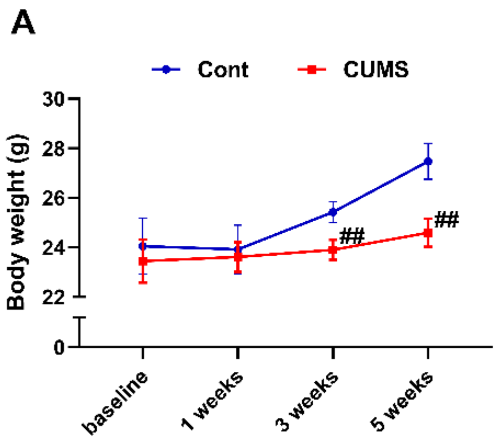

Table Analyzed	body weight
Column A	Cont
vs.	vs.
Column B	CUMS
Test details	
Test name	Unpaired t test
Variance assumption	Individual variance for each row
Multiple comparisons	Set P value threshold
Method	Bonferroni-Dunn method
Alpha	0.05

Body Weight	Cont							
baseline	24.8	23.6	23.1	22.2	26	24.3	24	24.4
1 weeks	24.5	25.2	23.1	22.5	24.9	23	24.4	23.7
3 weeks	25.5	26	24.9	24.8	25.3	25.8	25.7	25.4
5 weeks	27.4	26.7	26.9	27	29	27.9	27.5	27.4
	CUMS							
baseline	24.8	23.7	24	23.1	23.7	23.8	22.3	22.2
1 weeks	23	23.9	24.8	23.2	23.9	23.6	23.5	23
3 weeks	23.5	24.4	23.9	24.6	23.6	23.8	23.5	23.9
5 weeks	24.8	24	23.9	24.8	24	25.3	25.3	24.6

Groups	P value	Mean of Cont	Mean of CUMS	Difference	SE of difference	t ratio	DF	Adjusted P Value
Baseline	0.257371	24.05	23.45	0.6	0.5081	1.181	14	>0.999999
1 weeks	0.472792	23.91	23.61	0.3	0.4066	0.7379	14	>0.999999
3 weeks	0.000003	25.43	23.9	1.525	0.2068	7.374	14	0.000014
5 weeks	<0.000001	27.48	24.59	2.888	0.3258	8.863	14	0.000002

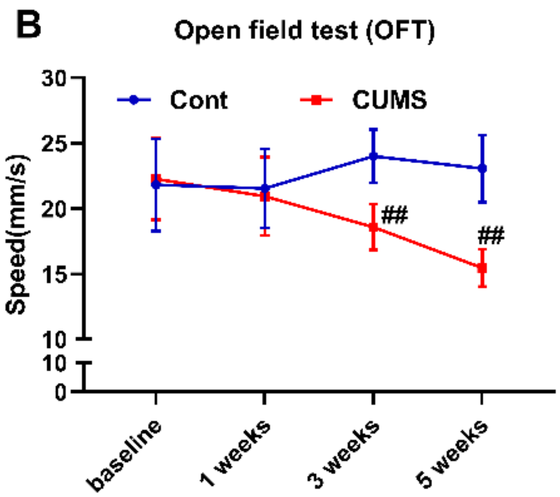

Table Analyzed	Speed (OFT)
Column A	Cont
vs.	vs.
Column B	CUMS
Test details	
Test name	Unpaired t test
Variance assumption	Individual variance for each row
Multiple comparisons	Set P value threshold
Method	Bonferroni-Dunn method
Alpha	0.05

**Speed**	**Cont**							
baseline	21.82555556	23.16777778	24.98944444	19.93611111	19.75	15.72166667	21.90277778	27.26888889
1 weeks	19.60333333	24.27888889	24.98944444	19.93611111	20.86111111	16.27722222	24.68055556	21.71333333
3 weeks	26.15777778	22.01444444	27.675	22.03722222	22.72333333	24.40666667	24.16666667	22.89888889
5 weeks	21.71333333	23.12555556	21.56388889	18.14833333	24.94555556	24.40666667	26.38888889	24.16833333
	**CUMS**							
baseline	22.01444444	27.11944	22.03722	24.94556	24.40667	18.64278	20.77778	18.16667
1 weeks	23.23055556	21.48167	24.39	24.40667	18.64278	20.77778	18.16667	16.41111
3 weeks	21.82555556	17.61222	17.21167	16.04722	19.75	18.49944	19.125	18.58111
5 weeks	16.27	17.61222	13.87833	14.38056	16.80556	15.72167	13.56944	15.46222
Groups	P value	Mean of Cont	Mean of CUMS	Difference	SE of difference	t ratio	DF	Adjusted P Value
Baseline	0.793117	21.82	22.26	-0.4435	1.659	0.2673	14	>0.999999
1 weeks	0.694076	21.54	20.94	0.6041	1.504	0.4016	14	>0.999999
3 weeks	0.000053	24.01	18.58	5.428	0.9489	5.721	14	0.000212
5 weeks	0.000004	23.06	15.46	7.595	1.037	7.321	14	0.000015

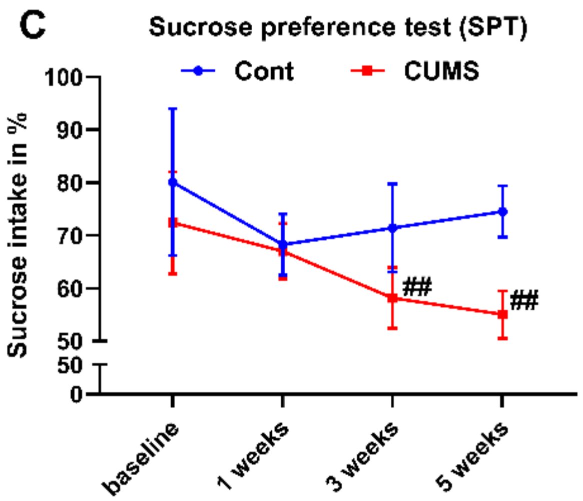

Table Analysed	SPT
Column A	Cont
vs.	vs.
Column B	CUMS
Test details	
Test name	Unpaired t test
Variance assumption	Individual variance for each row
Multiple comparisons	Set P value threshold
Method	Holm-Šídák method
Alpha	0.05

SPT	Cont							
baseline	83.5	76.1	83.6	90.6	78.8	96.2	82.2	49.5
1 weeks	73.4	70	71.6	57.3	69.3	61.4	72.6	70.5
3 weeks	59	70	79.4	62.5	69.6	84.6	75	71
5 weeks	67	77	79.4	72.5	69.6	81.6	75	74
	**CUMS**							
baseline	78.1	60.2	80.9	72.1	70	60	87.6	70.1
1 weeks	65.3	68.9	70.4	70.7	55.9	63.8	69	72
3 weeks	50.9	64.6	66.3	60.3	58	50	57	58
5 weeks	53.9	57.6	61.3	60.3	49	53	50	55

Groups	P value	Mean of Cont	Mean of CUMS	Difference	SE of difference	t ratio	DF	Adjusted P Value
Baseline	0.219317	80.06	72.38	7.688	5.978	1.286	14	0.390534
1 weeks	0.654507	68.26	67	1.263	2.761	0.4572	14	0.654507
3 weeks	0.002431	71.39	58.14	13.25	3.592	3.689	14	0.007274
5 weeks	<0.000001	74.51	55.01	19.5	2.338	8.34	14	0.000003

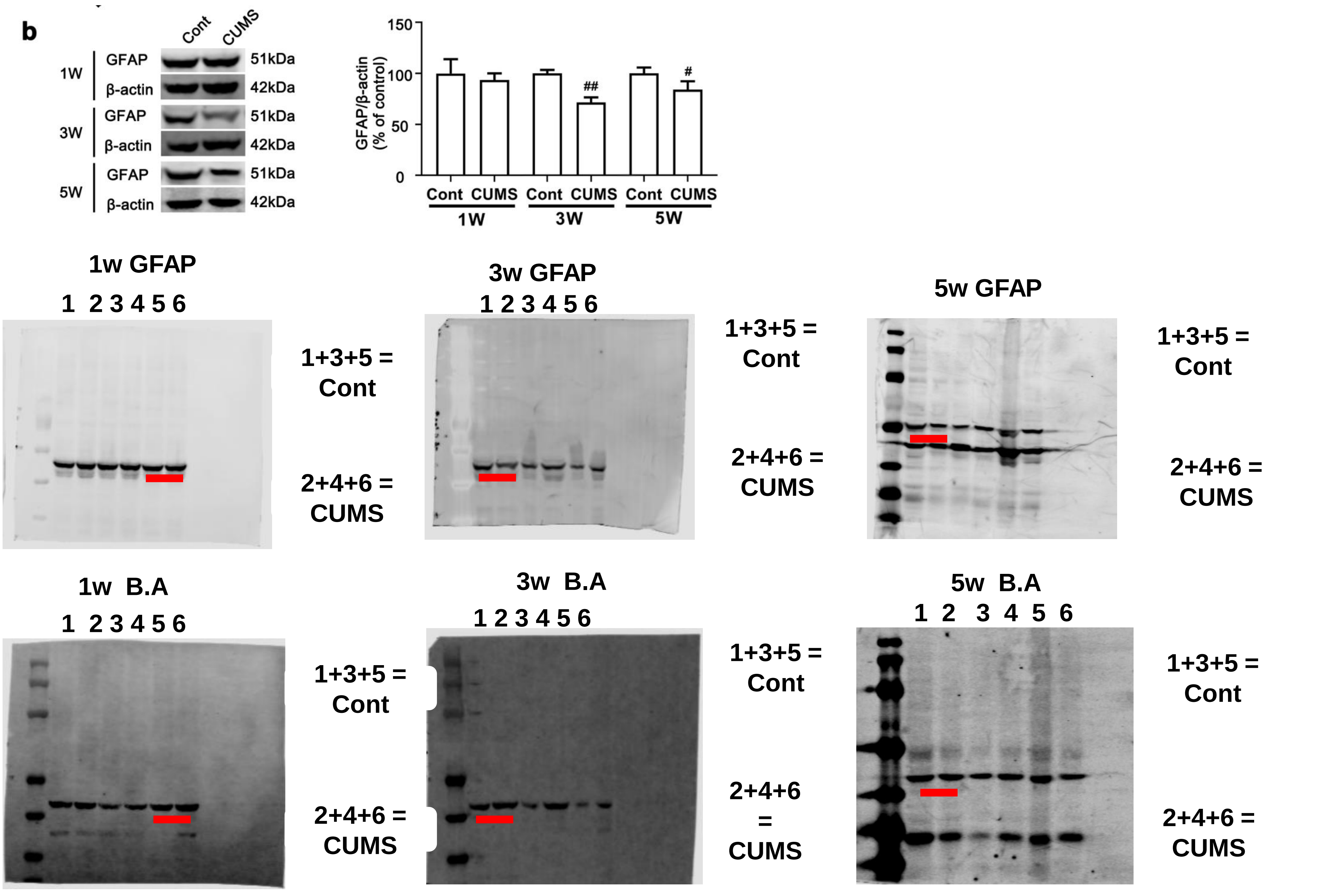

b
Cont
CUMS
1W
GFAP
β-actin
3W
GFAP
β-actin
5W
GFAP
β-actin
51kDa
42kDa
GFAP/β-actin (% of control)
150
100
50
0
##
#
Cont CUMS Cont CUMS Cont CUMS
1W 3W 5W
1w GFAP
1 2 3 4 5 6
1+3+5 = Cont
2+4+6 = CUMS
3w GFAP
1 2 3 4 5 6
1+3+5 = Cont
2+4+6 = CUMS
5w GFAP
1+3+5 = Cont
2+4+6 = CUMS
1w B.A
1 2 3 4 5 6
1+3+5 = Cont
2+4+6 = CUMS
3w B.A
1 2 3 4 5 6
1+3+5 = Cont
2+4+6 = CUMS
5w B.A
1 2 3 4 5 6
1+3+5 = Cont
2+4+6 = CUMS

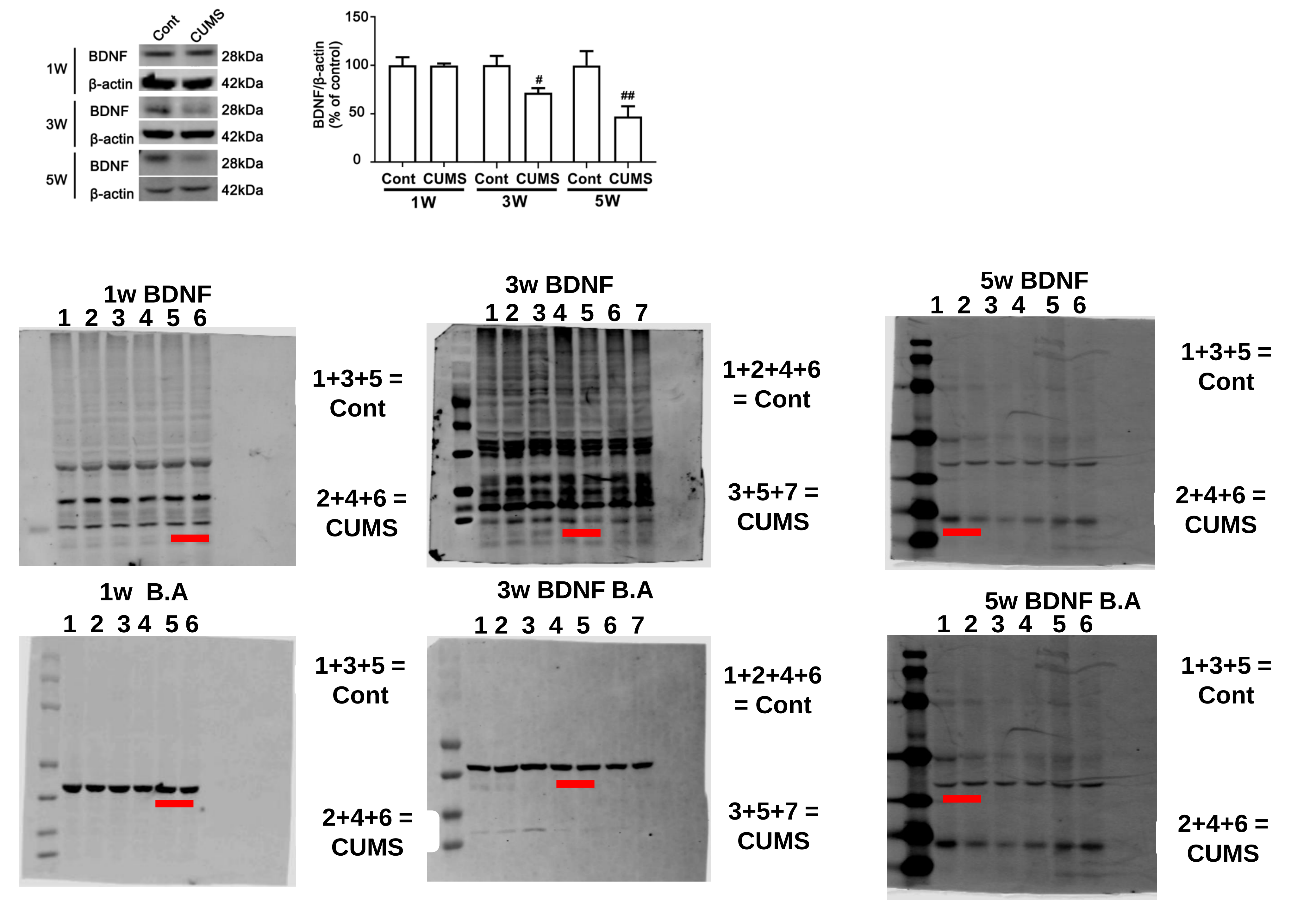

Cont
CUMS
1W
BDNF
β-actin
3W
5W
28kDa
42kDa
BDNF/β-actin (% of control)
150
100
50
0
#
##
Cont CUMS Cont CUMS Cont CUMS
1W 3W 5W
1w BDNF
1 2 3 4 5 6
1+3+5 = Cont
2+4+6 = CUMS
3w BDNF
1 2 3 4 5 6 7
1+2+4+6 = Cont
3+5+7 = CUMS
5w BDNF
1 2 3 4 5 6
1+3+5 = Cont
2+4+6 = CUMS
1w B.A
1 2 3 4 5 6
1+3+5 = Cont
2+4+6 = CUMS
3w BDNF B.A
1 2 3 4 5 6 7
1+2+4+6 = Cont
3+5+7 = CUMS
5w BDNF B.A
1 2 3 4 5 6
1+3+5 = Cont
2+4+6 = CUMS

B

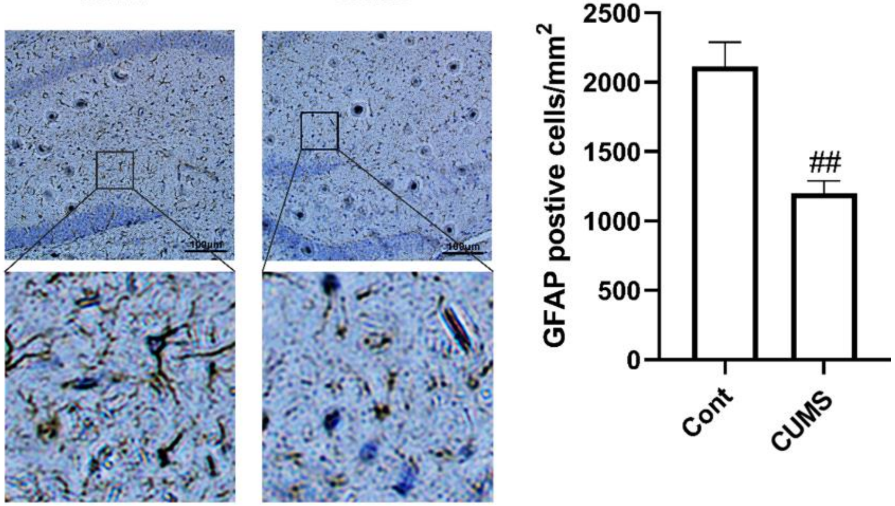

GFAP Positive cells/mm2		
	**Cont**	**CUMS**
	1800	1300
	2200	1100
	2200	1100
	2300	1200
	2100	1300
	2100	1200
**Average**	1658.333333	1200

Table Analysed	Data 1
Column B	CUMS
vs.	vs.
Column A	Cont
Unpaired t test	
P value	<0.0001
P value summary	****
Significantly different (P < 0.05)?	Yes
One- or two-tailed P value?	Two-tailed
t, df	t=11.57, df=10
How big is the difference?	
Mean of column A	2117
Mean of column B	1200
Difference between means (B - A) ± SEM	-916.7 ± 79.23
95% confidence interval	-1093 to -740.1
R squared (eta squared)	0.9305

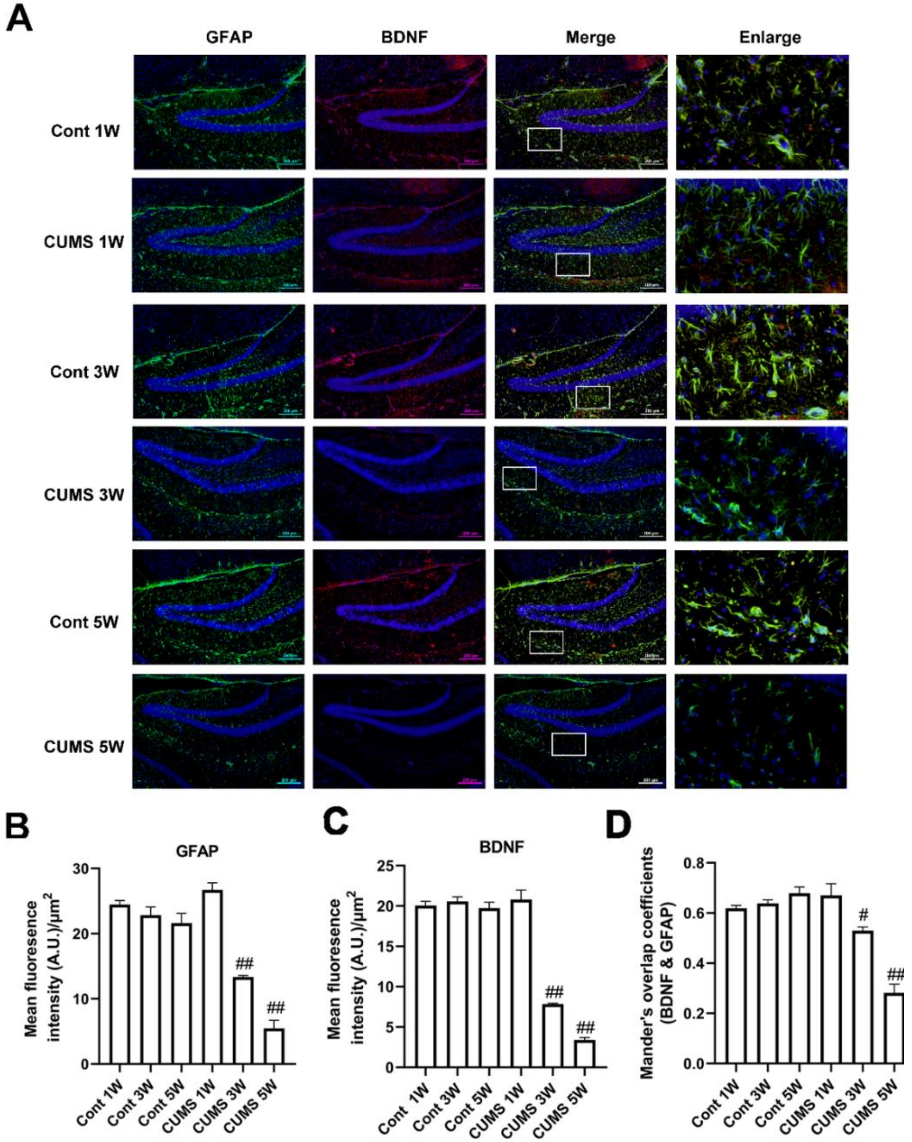

A
GFAP
BDNF
Merge
Enlarge
Cont 1W
CUMS 1W
Cont 3W
CUMS 3W
Cont 5W
CUMS 5W
B
GFAP
Mean fluoresence intensity (A.U.)/μm2
C
BDNF
Mean fluoresence intensity (A.U.)/μm2
D
Mander's overlap coefficients (BDNF & GFAP)
Cont 1W
Cont 3W
Cont 5W
CUMS 1W
CUMS 3W
CUMS 5W
##
#

CUMS induces reduction of BDNF levels in mice hippocampus.

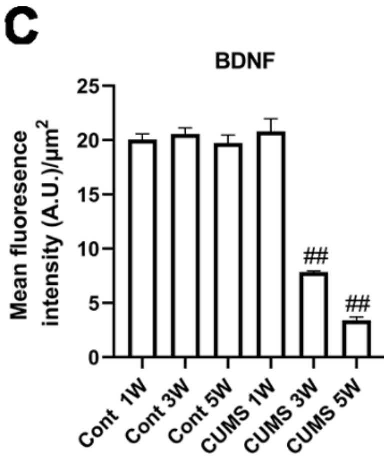

BDNF	Cont 1W	Cont 3W	Cont 5W	CUMS 1W	CUMS 3W	CUMS 5W
Mean fluorescence	20.589	20.768	19.257	20.948	7.943	3.754
intensity (A.U.)/μm2	19.581	21.007	19.406	21.895	7.843	3.242
Average	20	19.947	20.574	19.559	7.741	3.208

Tukey's multiple comparisons test	Mean Diff.	95.00% CI of diff.	Below threshold?	Summary	Adjusted P Value	q	DF
Cont 1W vs. Cont 3W	-0.5173	-2.312 to 1.278	No	ns	0.9196	1.369	12
Cont 1W vs. Cont 5W	0.311	-1.484 to 2.106	No	ns	0.9904	0.8231	12
Cont 1W vs. CUMS 1W	-0.744	-2.539 to 1.051	No	ns	0.7309	1.969	12
Cont 1W vs. CUMS 3W	12.21	10.42 to 14.01	Yes	****	<0.0001	32.33	12
Cont 1W vs. CUMS 5W	16.66	14.86 to 18.45	Yes	****	<0.0001	44.08	12
Cont 3W vs. Cont 5W	0.8283	-0.9666 to 2.623	No	ns	0.6421	2.192	12
Cont 3W vs. CUMS 1W	-0.2267	-2.022 to 1.568	No	ns	0.9978	0.5999	12
Cont 3W vs. CUMS 3W	12.73	10.94 to 14.53	Yes	****	<0.0001	33.69	12
Cont 3W vs. CUMS 5W	17.17	15.38 to 18.97	Yes	****	<0.0001	45.45	12
Cont 5W vs. CUMS 1W	-1.055	-2.850 to 0.7399	No	ns	0.4082	2.792	12
Cont 5W vs. CUMS 3W	11.9	10.11 to 13.70	Yes	****	<0.0001	31.5	12
Cont 5W vs. CUMS 5W	16.34	14.55 to 18.14	Yes	****	<0.0001	43.26	12
CUMS 1W vs. CUMS 3W	12.96	11.16 to 14.75	Yes	****	<0.0001	34.29	12
CUMS 1W vs. CUMS 5W	17.4	15.60 to 19.19	Yes	****	<0.0001	46.05	12
CUMS 3W vs. CUMS 5W	4.441	2.646 to 6.236	Yes	****	<0.0001	11.75	12

CUMS induces reduction of BDNF levels in mice hippocampus.

B

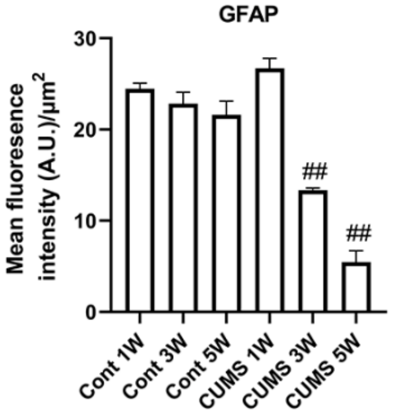

GFAP	Cont 1W	Cont 3W	Cont 5W	CUMS 1W	CUMS 3W	CUMS 5W
Mean fluorescence	24.247	23.11	20.875	27.974	13.412	6.9
intensity (A.U.)/µm2	25.167	21.5	20.59	26.224	13.124	4.765
	24	23.925	23.349	25.946	13.559	4.717

Tukey's multiple comparisons test	Mean Diff.	95.00% CI of diff.	Below threshold?	Summary	Adjusted P Value	q	DF
Cont 1W vs. Cont 3W	1.626	-1.339 to 4.592	No	ns	0.4772	2.605	12
Cont 1W vs. Cont 5W	2.867	-0.09889 to 5.832	No	ns	0.0603	4.592	12
Cont 1W vs. CUMS 1W	-2.243	-5.209 to 0.7222	No	ns	0.1867	3.593	12
Cont 1W vs. CUMS 3W	11.11	8.141 to 14.07	Yes	****	<0.0001	17.79	12
Cont 1W vs. CUMS 5W	19.01	16.05 to 21.98	Yes	****	<0.0001	30.45	12
Cont 3W vs. Cont 5W	1.24	-1.725 to 4.206	No	ns	0.7241	1.987	12
Cont 3W vs. CUMS 1W	-3.87	-6.835 to -0.9041	Yes	**	0.0089	6.198	12
Cont 3W vs. CUMS 3W	9.48	6.514 to 12.45	Yes	****	<0.0001	15.19	12
Cont 3W vs. CUMS 5W	17.38	14.42 to 20.35	Yes	****	<0.0001	27.85	12
Cont 5W vs. CUMS 1W	-5.11	-8.076 to -2.144	Yes	***	0.0009	8.185	12
Cont 5W vs. CUMS 3W	8.24	5.274 to 11.21	Yes	****	<0.0001	13.2	12
Cont 5W vs. CUMS 5W	16.14	13.18 to 19.11	Yes	****	<0.0001	25.86	12
CUMS 1W vs. CUMS 3W	13.35	10.38 to 16.32	Yes	****	<0.0001	21.38	12
CUMS 1W vs. CUMS 5W	21.25	18.29 to 24.22	Yes	****	<0.0001	34.04	12
CUMS 3W vs. CUMS 5W	7.904	4.939 to 10.87	Yes	****	<0.0001	12.66	12

CUMS induces reduction of BDNF levels in mice hippocampus.

D

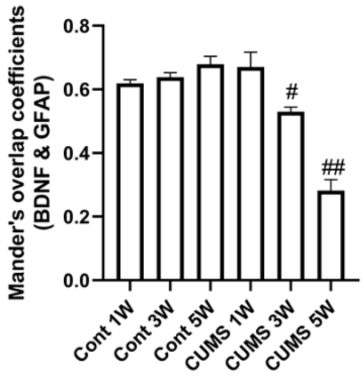

Groups	Cont 1W	Cont 3W	Cont 5W	CUMS 1W	CUMS 3W	CUMS 5W
Mander's overlap coefficients	0.608	0.647	0.682	0.621	0.528	0.322
(BDNF & GFAP)	0.618	0.646	0.702	0.679	0.517	0.261
	0.631	0.623	0.653	0.712	0.545	0.261

Tukey's multiple comparisons test	Mean Diff.	95.00% CI of diff.	Below threshold?	Summary	Adjusted P Value	q	DF
Cont 1W vs. Cont 3W	-0.01967	-0.09466 to 0.05532	No	ns	0.9441	1.246	12
Cont 1W vs. Cont 5W	-0.06	-0.1350 to 0.01499	No	ns	0.1491	3.801	12
Cont 1W vs. CUMS 1W	-0.05167	-0.1267 to 0.02332	No	ns	0.2601	3.273	12
Cont 1W vs. CUMS 3W	0.089	0.01401 to 0.1640	Yes	*	0.0173	5.638	12
Cont 1W vs. CUMS 5W	0.3377	0.2627 to 0.4127	Yes	****	<0.0001	21.39	12
Cont 3W vs. Cont 5W	-0.04033	-0.1153 to 0.03466	No	ns	0.4965	2.555	12
Cont 3W vs. CUMS 1W	-0.032	-0.1070 to 0.04299	No	ns	0.7083	2.027	12
Cont 3W vs. CUMS 3W	0.1087	0.03368 to 0.1837	Yes	**	0.004	6.883	12
Cont 3W vs. CUMS 5W	0.3573	0.2823 to 0.4323	Yes	****	<0.0001	22.63	12
Cont 5W vs. CUMS 1W	0.008333	-0.06666 to 0.08332	No	ns	0.9988	0.5279	12
Cont 5W vs. CUMS 3W	0.149	0.07401 to 0.2240	Yes	***	0.0003	9.438	12
Cont 5W vs. CUMS 5W	0.3977	0.3227 to 0.4727	Yes	****	<0.0001	25.19	12
CUMS 1W vs. CUMS 3W	0.1407	0.06568 to 0.2157	Yes	***	0.0004	8.91	12
CUMS 1W vs. CUMS 5W	0.3893	0.3143 to 0.4643	Yes	****	<0.0001	24.66	12
CUMS 3W vs. CUMS 5W	0.2487	0.1737 to 0.3237	Yes	****	<0.0001	15.75	12

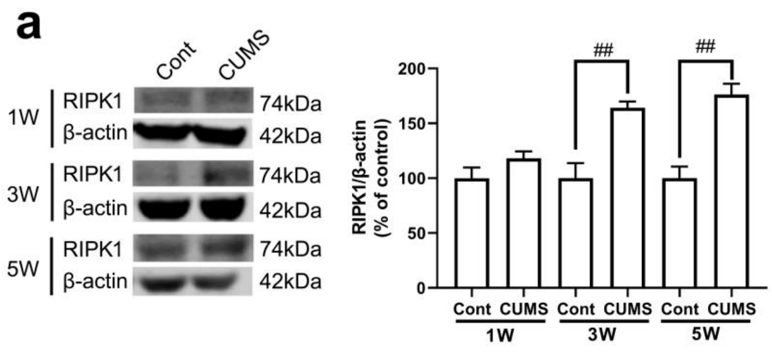

1w RIP1

1 2 3 4 5 6

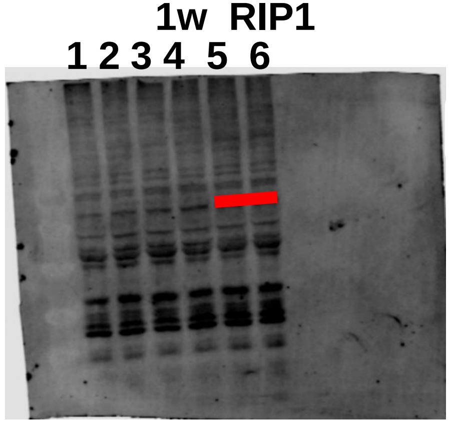

1+3+5 = Cont

2+4+6 = CUMS

3w RIP1

1 2 3 4 5 6

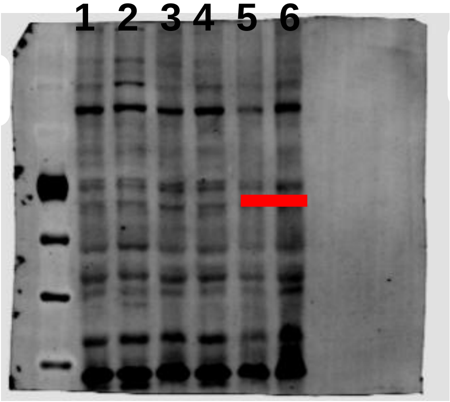

1+3+5= Cont

2+4+6 = CUMS

5w RIP1

1 2 3 4 5 6 7 8 9 10

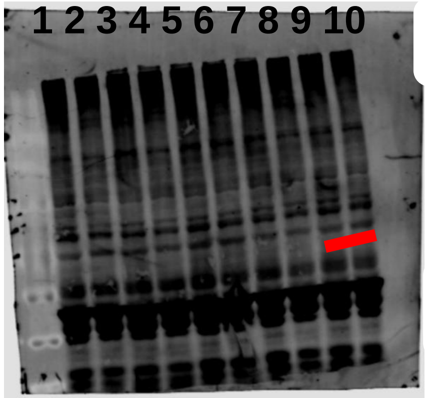

1+3+5+7 +9 = Cont

2+4+6+8+ 10 = CUMS

1w B.A

1 2 3 4 5 6

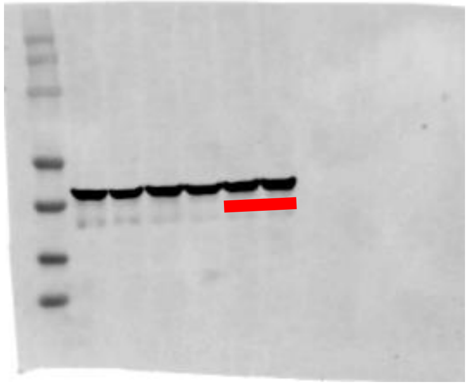

1+3+5 = Cont

2+4+6 = CUMS

3w RIP1 B.A

1 2 3 4 5 6

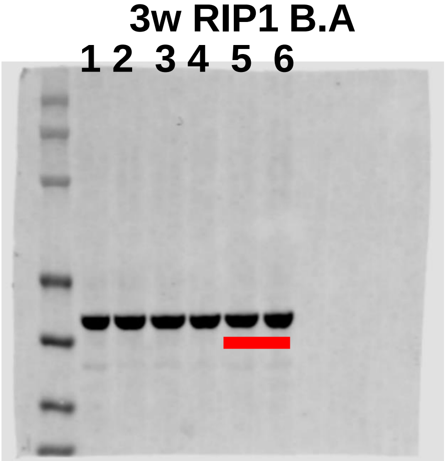

1+3+5 = Cont

2+4+6 = CUMS

5w RIP1 B.A

1 2 3 4 5 6 7 8 9 10

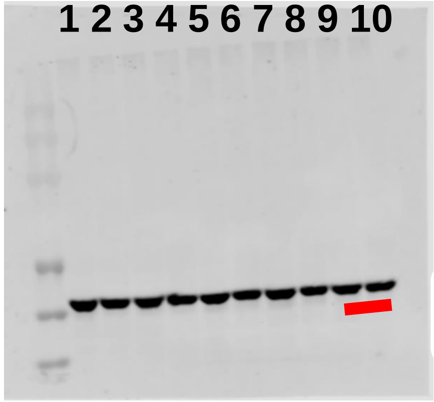

1+3+5+7 +9 = Cont

2+4+6+8+ 10 = CUMS

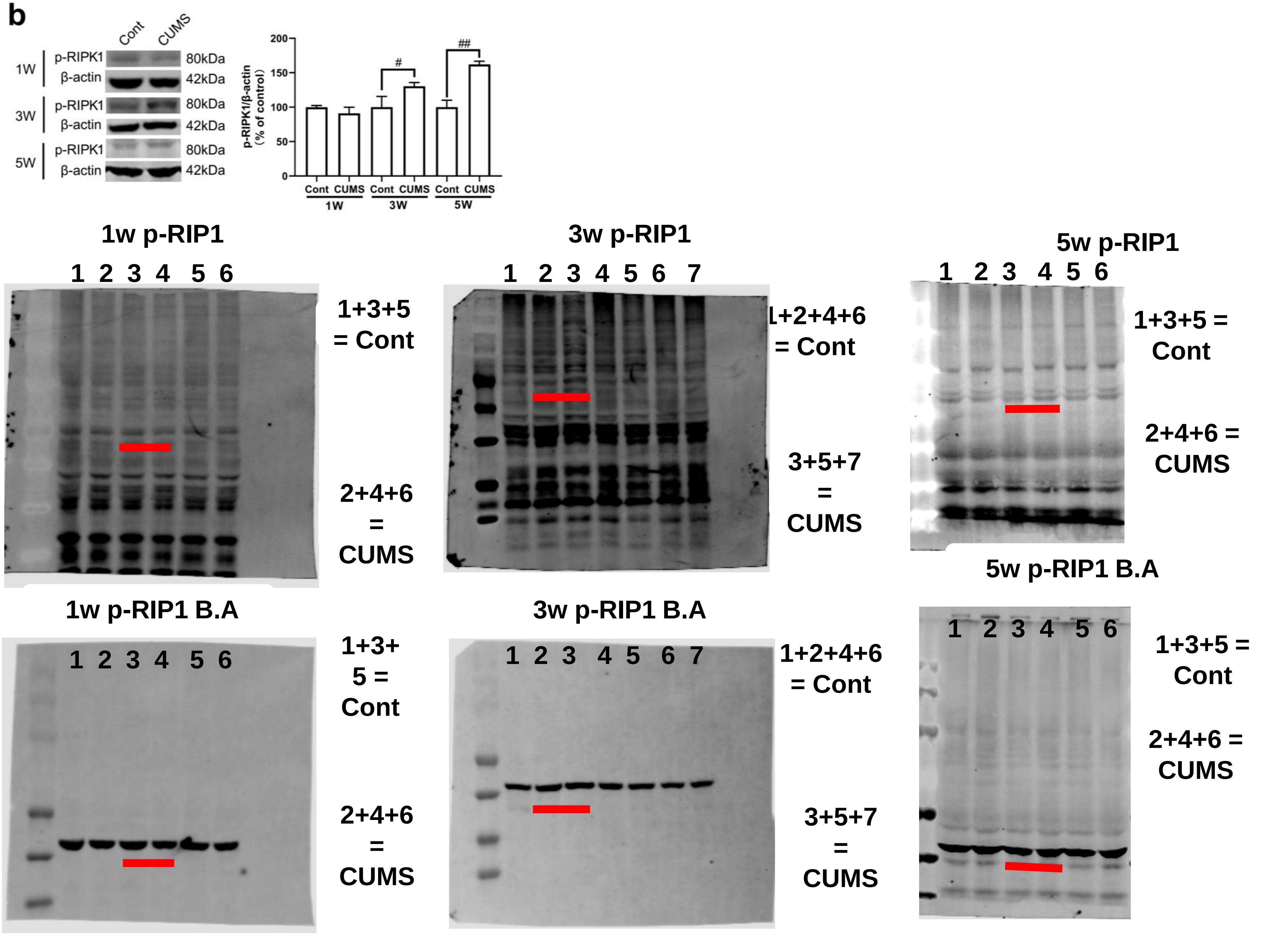

b
Cont
CUMS
1W
p-RIPK1
β-actin
80kDa
42kDa
3W
p-RIPK1
β-actin
80kDa
42kDa
5W
p-RIPK1
β-actin
80kDa
42kDa
p-RIPK1/β-actin (% of control)
200
150
100
50
0
#
##
Cont CUMS Cont CUMS Cont CUMS
1W 3W 5W
1w p-RIP1
1 2 3 4 5 6
1+3+5 = Cont
2+4+6 = CUMS
3w p-RIP1
1 2 3 4 5 6 7
1+2+4+6 = Cont
3+5+7 = CUMS
5w p-RIP1
1 2 3 4 5 6
1+3+5 = Cont
2+4+6 = CUMS
1w p-RIP1 B.A
1 2 3 4 5 6
1+3+5 = Cont
2+4+6 = CUMS
3w p-RIP1 B.A
1 2 3 4 5 6 7
1+2+4+6 = Cont
3+5+7 = CUMS
5w p-RIP1 B.A
1 2 3 4 5 6
1+3+5 = Cont
2+4+6 = CUMS

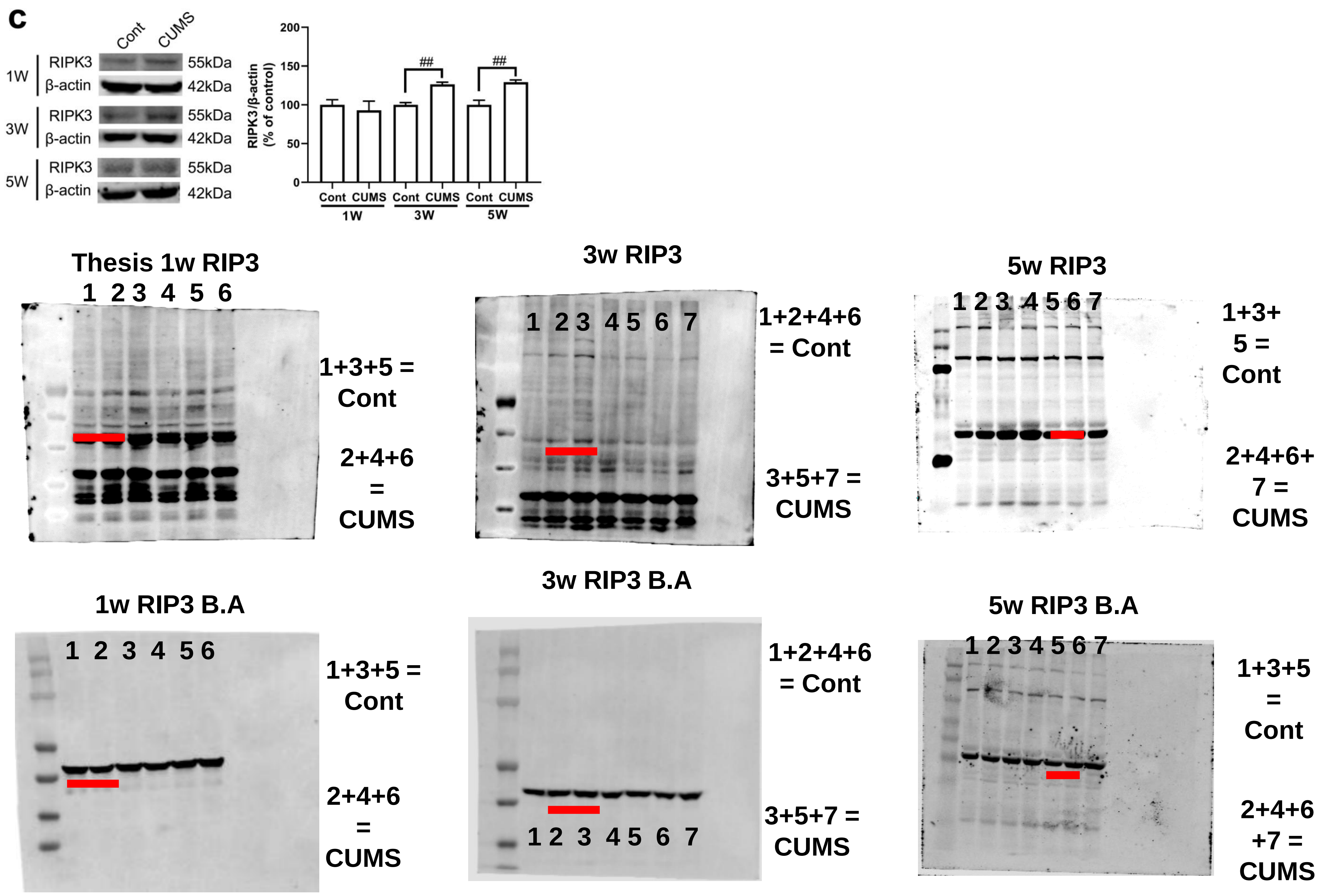

c
Cont
CUMS
1W
3W
5W
RIPK3
β-actin
55kDa
42kDa
RIPK3/β-actin (% of control)
200
150
100
50
0
##
##
Cont CUMS
1W
Cont CUMS
3W
Cont CUMS
5W
Thesis 1w RIP3
1 2 3 4 5 6
1+3+5 = Cont
2+4+6 = CUMS
3w RIP3
1 2 3 4 5 6 7
1+2+4+6 = Cont
3+5+7 = CUMS
5w RIP3
1 2 3 4 5 6 7
1+3+5 = Cont
2+4+6+7 = CUMS
1w RIP3 B.A
1 2 3 4 5 6
1+3+5 = Cont
2+4+6 = CUMS
3w RIP3 B.A
1 2 3 4 5 6 7
1+2+4+6 = Cont
3+5+7 = CUMS
5w RIP3 B.A
1 2 3 4 5 6 7
1+3+5 = Cont
2+4+6+7 = CUMS

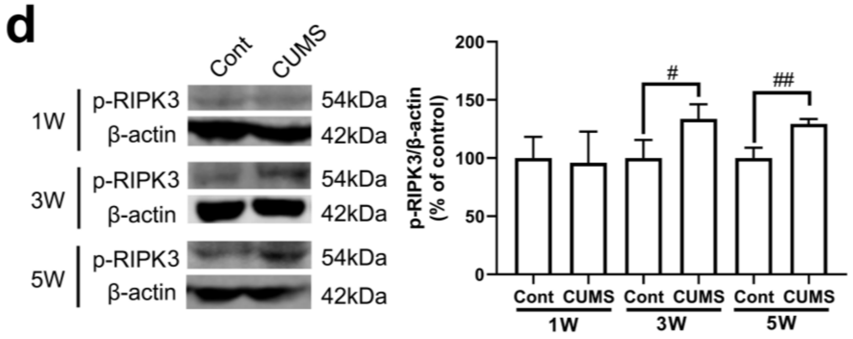

d
Cont
CUMS
1W
p-RIPK3
54kDa
β-actin
42kDa
3W
p-RIPK3
54kDa
β-actin
42kDa
5W
p-RIPK3
54kDa
β-actin
42kDa
p-RIPK3/β-actin (% of control)
200
150
100
50
0
#
##
Cont CUMS Cont CUMS Cont CUMS
1W
3W
5W

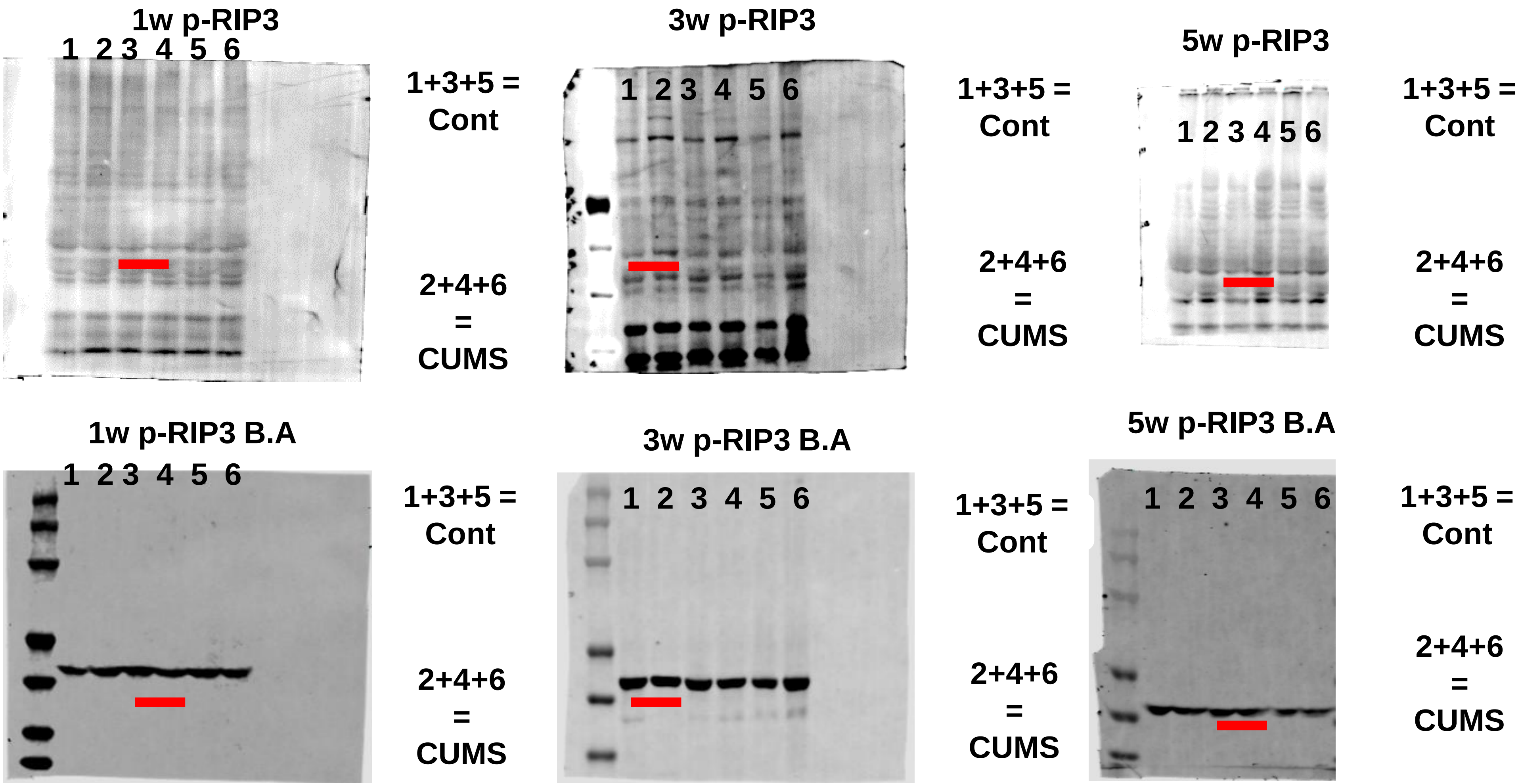

1w p-RIP3
1 2 3 4 5 6
1+3+5 = Cont
2+4+6 = CUMS
3w p-RIP3
1 2 3 4 5 6
1+3+5 = Cont
2+4+6 = CUMS
5w p-RIP3
1 2 3 4 5 6
1+3+5 = Cont
2+4+6 = CUMS
1w p-RIP3 B.A
1 2 3 4 5 6
1+3+5 = Cont
2+4+6 = CUMS
3w p-RIP3 B.A
1 2 3 4 5 6
1+3+5 = Cont
2+4+6 = CUMS
5w p-RIP3 B.A
1 2 3 4 5 6
1+3+5 = Cont
2+4+6 = CUMS

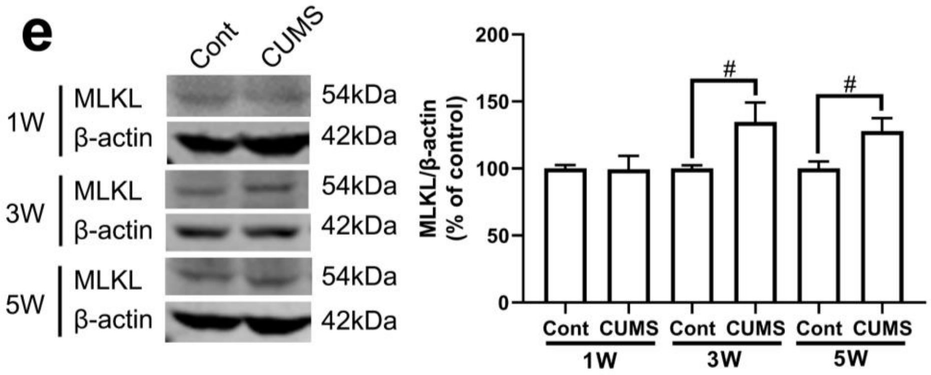

1w MLKL

1 2 3 4 5 6

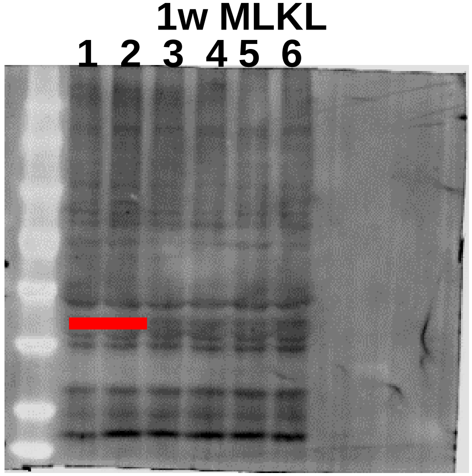

1+3+5 = Cont

2+4+6 = CUMS

3w MLKL

1 2 3 4 5 6

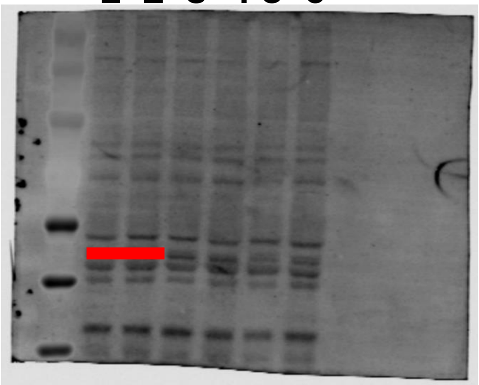

1+3+5 = Cont

2+4+6 = CUMS

5w MLKL

1 2 3 4 5 6

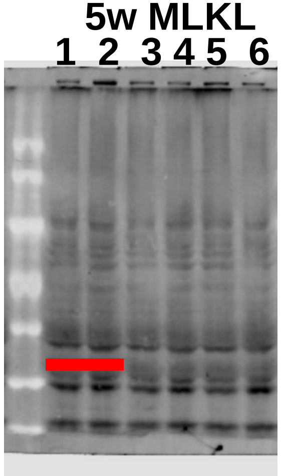

1+3+5 = Cont

2+4+6 = CUMS

1w MLKL B.A

1 2 3 4 5 6

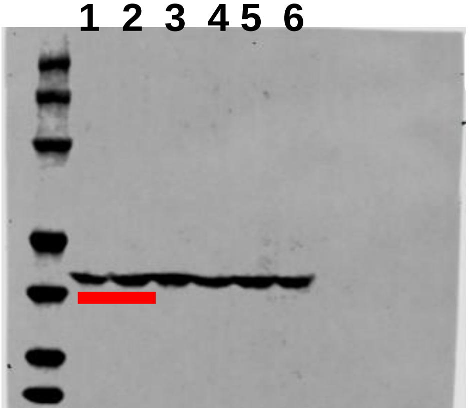

1+3+5 = Cont

2+4+6 = CUMS

3w MLKL B.A

1 2 3 4 5 6

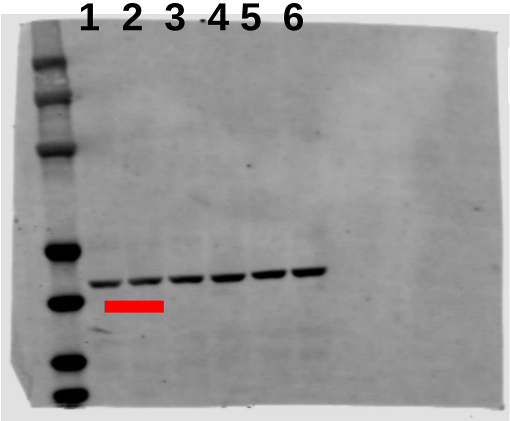

1+3+5 = Cont

2+4+6 = CUMS

5w MLKL B.A

1 2 3 4 5 6

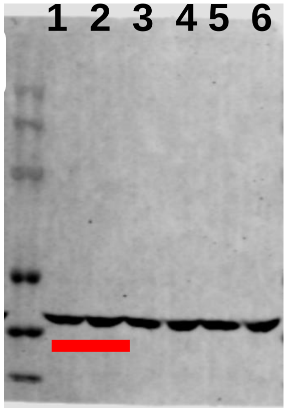

1+3+5 = Cont

2+4+6 = CUMS

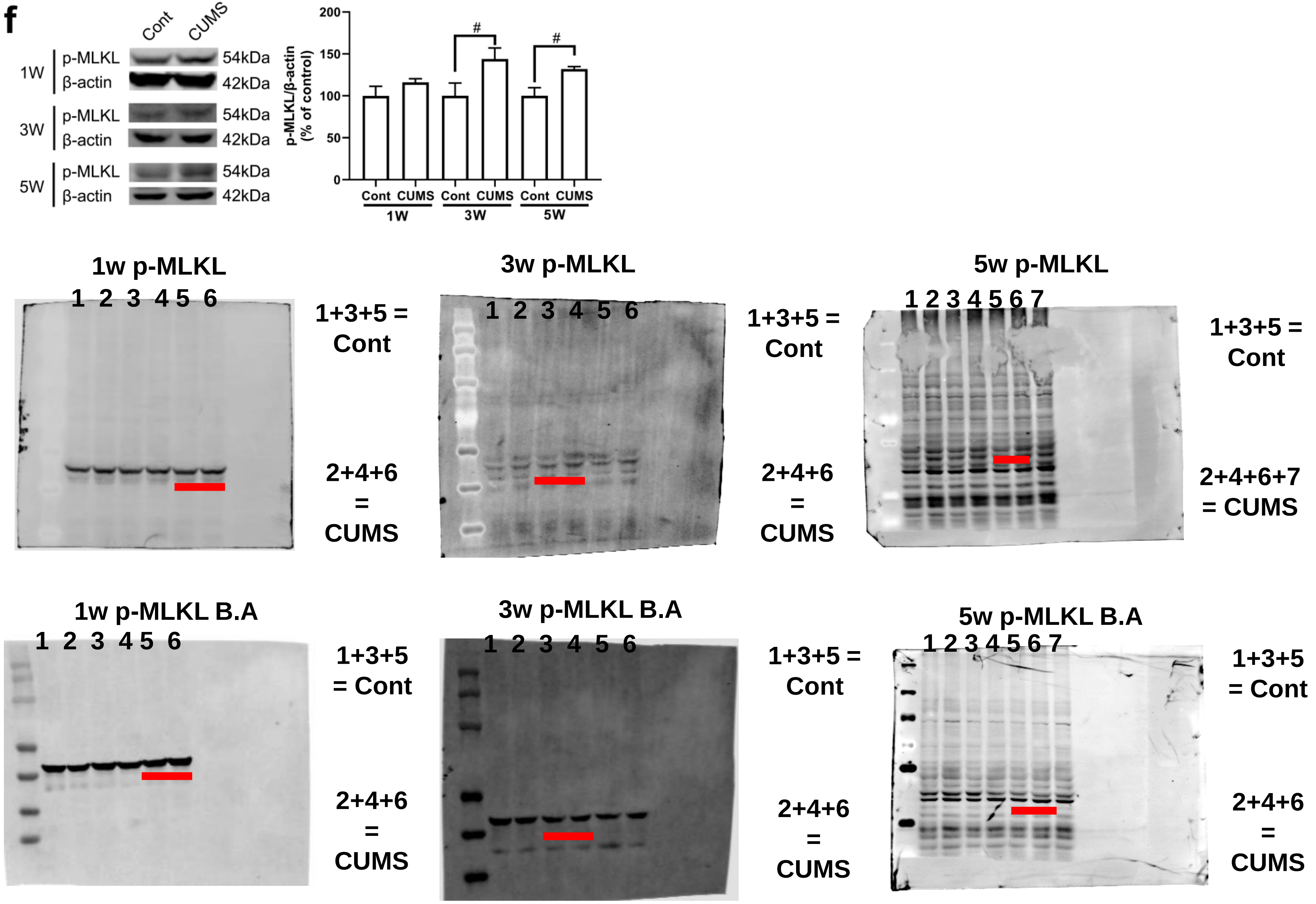
f
Cont
CUMS
1W
p-MLKL
β-actin
54kDa
42kDa
3W
p-MLKL
β-actin
54kDa
42kDa
5W
p-MLKL
β-actin
54kDa
42kDa
p-MLKL/β-actin (% of control)
200
150
100
50
0
#
#
Cont CUMS Cont CUMS Cont CUMS
1W 3W 5W
1w p-MLKL
1 2 3 4 5 6
1+3+5 = Cont
2+4+6 = CUMS
3w p-MLKL
1 2 3 4 5 6
1+3+5 = Cont
2+4+6 = CUMS
5w p-MLKL
1 2 3 4 5 6 7
1+3+5 = Cont
2+4+6+7 = CUMS
1w p-MLKL B.A
1 2 3 4 5 6
1+3+5 = Cont
2+4+6 = CUMS
3w p-MLKL B.A
1 2 3 4 5 6
1+3+5 = Cont
2+4+6 = CUMS
5w p-MLKL B.A
1 2 3 4 5 6 7
1+3+5 = Cont
2+4+6 = CUMS

The expression of RIP1K is increased in the hippocampal astrocytes of CUMS mice in a time-dependent manner.

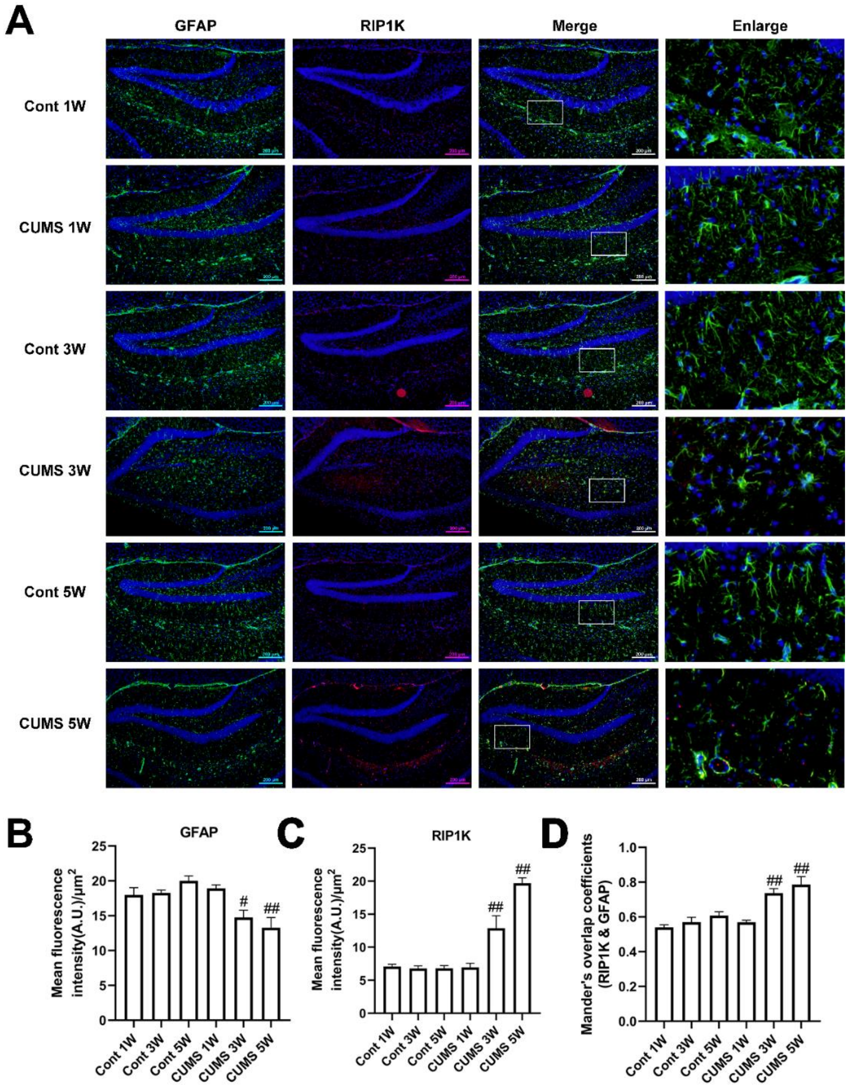

The expression of RIP1K is increased in the hippocampal astrocytes of CUMS mice in a time-dependent manner.

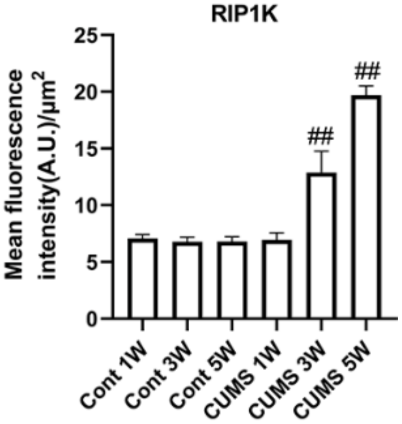

RIP1K	Cont 1W	Cont 3W	Cont 5W	CUMS 1W	CUMS 3W	CUMS 5W
Mean fluorescence	7.057	6.525	6.509	7.665	13.985	20.456
intensity (A.U.)/μm2	6.715	6.622	7.267	6.616	10.713	19.807
	7.438	7.224	6.643	6.537	13.947	18.859

Tukey's multiple comparisons test	Mean Diff.	95.00% CI of diff.	Below threshold?	Summary	Adjusted P Value	q	DF
Cont 1W vs. Cont 3W	0.2797	-2.226 to 2.785	No	ns	0.9988	0.5303	12
Cont 1W vs. Cont 5W	0.2637	-2.242 to 2.769	No	ns	0.9991	0.4999	12
Cont 1W vs. CUMS 1W	0.1307	-2.375 to 2.636	No	ns	>0.9999	0.2477	12
Cont 1W vs. CUMS 3W	-5.812	-8.317 to -3.306	Yes	****	<0.0001	11.02	12
Cont 1W vs. CUMS 5W	-12.64	-15.14 to -10.13	Yes	****	<0.0001	23.96	12
Cont 3W vs. Cont 5W	-0.016	-2.521 to 2.489	No	ns	>0.9999	0.03034	12
Cont 3W vs. CUMS 1W	-0.149	-2.654 to 2.356	No	ns	>0.9999	0.2825	12
Cont 3W vs. CUMS 3W	-6.091	-8.597 to -3.586	Yes	****	<0.0001	11.55	12
Cont 3W vs. CUMS 5W	-12.92	-15.42 to -10.41	Yes	****	<0.0001	24.49	12
Cont 5W vs. CUMS 1W	-0.133	-2.638 to 2.372	No	ns	>0.9999	0.2522	12
Cont 5W vs. CUMS 3W	-6.075	-8.581 to -3.570	Yes	****	<0.0001	11.52	12
Cont 5W vs. CUMS 5W	-12.9	-15.41 to -10.40	Yes	****	<0.0001	24.46	12
CUMS 1W vs. CUMS 3W	-5.942	-8.448 to -3.437	Yes	****	<0.0001	11.27	12
CUMS 1W vs. CUMS 5W	-12.77	-15.27 to -10.26	Yes	****	<0.0001	24.21	12
CUMS 3W vs. CUMS 5W	-6.826	-9.331 to -4.320	Yes	****	<0.0001	12.94	12

The expression of RIP1K is increased in the hippocampal astrocytes of CUMS mice in a time-dependent manner.

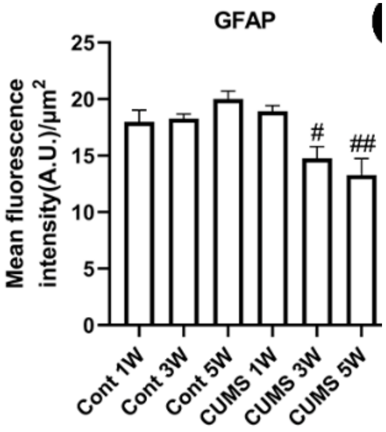

GFAP	Cont 1W	Cont 3W	Cont 5W	CUMS 1W	CUMS 3W	CUMS 5W
Mean fluorescence	17.235	18.051	19.192	19.515	15.952	14.727
intensity (A.U.)/μm2	17.554	18.748	20.545	18.649	13.972	13.306
	19.184	18.064	20.272	18.636	14.36	11.839

Tukey's multiple comparisons test	Mean Diff.	95.00% CI of diff.	Below threshold?	Summary	Adjusted P Value	q	DF
Cont 1W vs. Cont 3W	-0.2967	-2.851 to 2.257	No	ns	0.9985	0.5518	12
Cont 1W vs. Cont 5W	-2.012	-4.566 to 0.5421	No	ns	0.159	3.742	12
Cont 1W vs. CUMS 1W	-0.9423	-3.496 to 1.612	No	ns	0.8102	1.753	12
Cont 1W vs. CUMS 3W	3.23	0.6756 to 5.784	Yes	*	0.0112	6.007	12
Cont 1W vs. CUMS 5W	4.7	2.146 to 7.254	Yes	***	0.0005	8.742	12
Cont 3W vs. Cont 5W	-1.715	-4.269 to 0.8388	No	ns	0.2823	3.19	12
Cont 3W vs. CUMS 1W	-0.6457	-3.200 to 1.908	No	ns	0.9517	1.201	12
Cont 3W vs. CUMS 3W	3.526	0.9722 to 6.080	Yes	**	0.0058	6.558	12
Cont 3W vs. CUMS 5W	4.997	2.443 to 7.551	Yes	***	0.0003	9.294	12
Cont 5W vs. CUMS 1W	1.07	-1.484 to 3.624	No	ns	0.723	1.989	12
Cont 5W vs. CUMS 3W	5.242	2.688 to 7.796	Yes	***	0.0002	9.749	12
Cont 5W vs. CUMS 5W	6.712	4.158 to 9.266	Yes	****	<0.0001	12.48	12
CUMS 1W vs. CUMS 3W	4.172	1.618 to 6.726	Yes	**	0.0015	7.759	12
CUMS 1W vs. CUMS 5W	5.643	3.089 to 8.197	Yes	****	<0.0001	10.49	12
CUMS 3W vs. CUMS 5W	1.471	-1.083 to 4.025	No	ns	0.4286	2.735	12

The expression of RIP1K is increased in the hippocampal astrocytes of CUMS mice in a time-dependent manner.

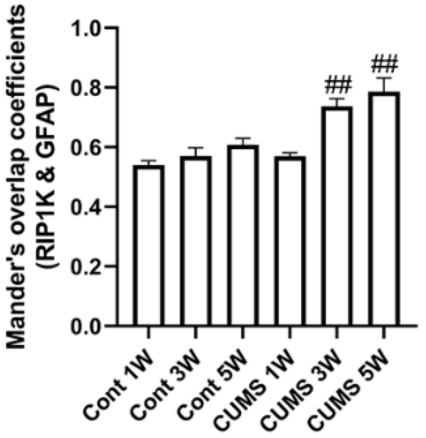

Groups	Cont 1W	Cont 3W	Cont 5W	CUMS 1W	CUMS 3W	CUMS 5W
Mander's overlap coefficients	0.524	0.587	0.623	0.565	0.754	0.837
(RIP1 & GFAP)	0.548	0.539	0.584	0.583	0.75	0.748
	0.549	0.586	0.618	0.56	0.707	0.774

Tukey's multiple comparisons test	Mean Diff.	95.00% CI of diff.	Below threshold?	Summary	Adjusted P Value	q	DF
Cont 1W vs. Cont 3W	-0.03033	-0.1039 to 0.04328	No	ns	0.7354	1.957	12
Cont 1W vs. Cont 5W	-0.068	-0.1416 to 0.005613	No	ns	0.0765	4.388	12
Cont 1W vs. CUMS 1W	-0.029	-0.1026 to 0.04461	No	ns	0.7679	1.871	12
Cont 1W vs. CUMS 3W	-0.1967	-0.2703 to -0.1231	Yes	****	<0.0001	12.69	12
Cont 1W vs. CUMS 5W	-0.246	-0.3196 to -0.1724	Yes	****	<0.0001	15.87	12
Cont 3W vs. Cont 5W	-0.03767	-0.1113 to 0.03595	No	ns	0.5455	2.431	12
Cont 3W vs. CUMS 1W	0.001333	-0.07228 to 0.07495	No	ns	>0.9999	0.08604	12
Cont 3W vs. CUMS 3W	-0.1663	-0.2399 to -0.09272	Yes	****	<0.0001	10.73	12
Cont 3W vs. CUMS 5W	-0.2157	-0.2893 to -0.1421	Yes	****	<0.0001	13.92	12
Cont 5W vs. CUMS 1W	0.039	-0.03461 to 0.1126	No	ns	0.5114	2.517	12
Cont 5W vs. CUMS 3W	-0.1287	-0.2023 to -0.05505	Yes	***	0.0008	8.303	12
Cont 5W vs. CUMS 5W	-0.178	-0.2516 to -0.1044	Yes	****	<0.0001	11.49	12
CUMS 1W vs. CUMS 3W	-0.1677	-0.2413 to -0.09405	Yes	****	<0.0001	10.82	12
CUMS 1W vs. CUMS 5W	-0.217	-0.2906 to -0.1434	Yes	****	<0.0001	14	12
CUMS 3W vs. CUMS 5W	-0.04933	-0.1229 to 0.02428	No	ns	0.2842	3.183	12

The expression of RIP3K is increased in the hippocampal astrocytes of CUMS mice in a time-dependent manner.

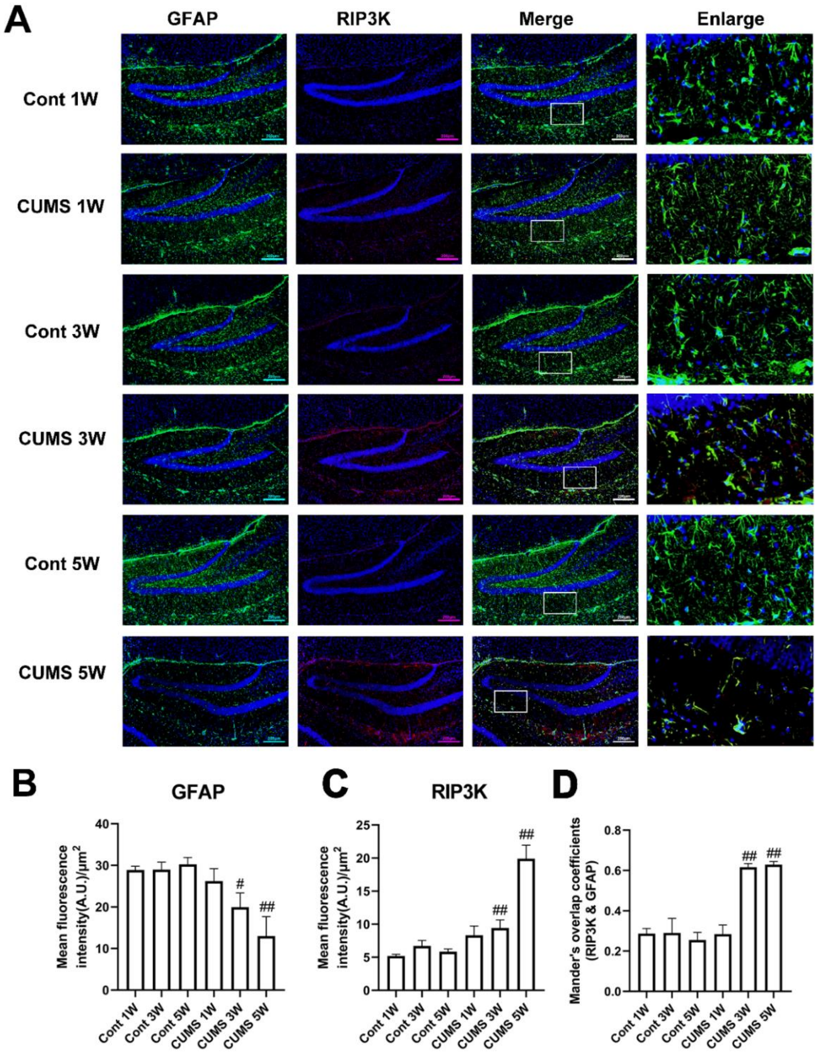

The expression of RIP3K is increased in the hippocampal astrocytes of CUMS mice in a time-dependent manner.

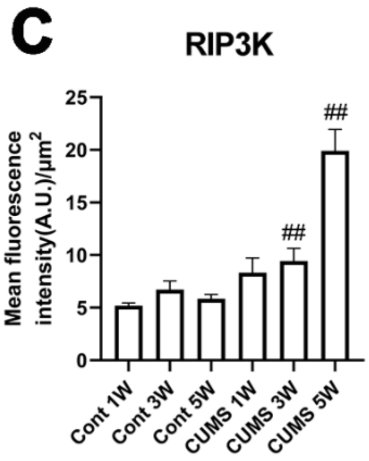

Groups	Cont 1W	Cont 3W	Cont 5W	CUMS 1W	CUMS 3W	CUMS 5W
RIPK3	5.399	6.134	6.313	7.559	8.423	19.932
Mean fluorescence	4.911	7.661	5.487	7.486	10.782	21.938
intensity (A.U.)/μm2	5.268	6.351	5.727	9.944	9.052	17.837

Tukey's multiple comparisons test	Mean Diff.	95.00% CI of diff.	Below threshold?	Summary	Adjusted P Value	q	DF
Cont 1W vs. Cont 3W	-1.523	-4.802 to 1.757	No	ns	0.6367	2.205	12
Cont 1W vs. Cont 5W	-0.6497	-3.929 to 2.630	No	ns	0.9827	0.941	12
Cont 1W vs. CUMS 1W	-3.137	-6.417 to 0.1426	No	ns	0.0638	4.544	12
Cont 1W vs. CUMS 3W	-4.226	-7.506 to -0.9467	Yes	**	0.0098	6.121	12
Cont 1W vs. CUMS 5W	-14.71	-17.99 to -11.43	Yes	****	<0.0001	21.31	12
Cont 3W vs. Cont 5W	0.873	-2.407 to 4.153	No	ns	0.9408	1.264	12
Cont 3W vs. CUMS 1W	-1.614	-4.894 to 1.665	No	ns	0.5828	2.338	12
Cont 3W vs. CUMS 3W	-2.704	-5.983 to 0.5760	No	ns	0.1312	3.916	12
Cont 3W vs. CUMS 5W	-13.19	-16.47 to -9.907	Yes	****	<0.0001	19.1	12
Cont 5W vs. CUMS 1W	-2.487	-5.767 to 0.7923	No	ns	0.1848	3.603	12
Cont 5W vs. CUMS 3W	-3.577	-6.856 to -0.2970	Yes	*	0.03	5.18	12
Cont 5W vs. CUMS 5W	-14.06	-17.34 to -10.78	Yes	****	<0.0001	20.36	12
CUMS 1W vs. CUMS 3W	-1.089	-4.369 to 2.190	No	ns	0.8659	1.578	12
CUMS 1W vs. CUMS 5W	-11.57	-14.85 to -8.293	Yes	****	<0.0001	16.76	12
CUMS 3W vs. CUMS 5W	-10.48	-13.76 to -7.204	Yes	****	<0.0001	15.18	12

The expression of RIP3K is increased in the hippocampal astrocytes of CUMS mice in a time-dependent manner.

B

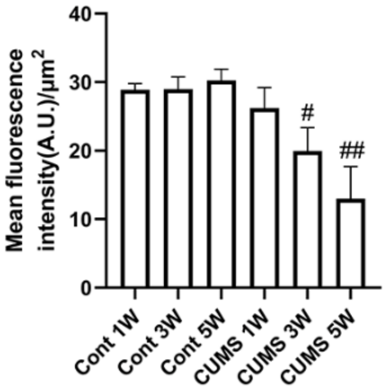

Groups	Cont 1W	Cont 3W	Cont 5W	CUMS 1W	CUMS 3W	CUMS 5W
GFAP	29.719	29.88	31.284	22.779	18.032	8.63
Mean fluoresence	27.938	26.944	28.373	27.832	17.971	17.949
intensity (A.U.)/μm2	28.975	30.16	31.114	28.054	23.883	12.391

Tukey's multiple comparisons test	Mean Diff.	95.00% CI of diff.	Below threshold?	Summary	Adjusted P Value	q	DF
Cont 1W vs. Cont 3W	-0.1173	-7.959 to 7.725	No	ns	>0.9999	0.07107	12
Cont 1W vs. Cont 5W	-1.38	-9.222 to 6.462	No	ns	0.9897	0.8357	12
Cont 1W vs. CUMS 1W	2.656	-5.186 to 10.50	No	ns	0.8567	1.609	12
Cont 1W vs. CUMS 3W	8.915	1.073 to 16.76	Yes	*	0.023	5.4	12
Cont 1W vs. CUMS 5W	15.89	8.045 to 23.73	Yes	***	0.0002	9.624	12
Cont 3W vs. Cont 5W	-1.262	-9.104 to 6.580	No	ns	0.9931	0.7647	12
Cont 3W vs. CUMS 1W	2.773	-5.069 to 10.61	No	ns	0.8345	1.68	12
Cont 3W vs. CUMS 3W	9.033	1.191 to 16.87	Yes	*	0.0212	5.472	12
Cont 3W vs. CUMS 5W	16	8.163 to 23.85	Yes	***	0.0002	9.695	12
Cont 5W vs. CUMS 1W	4.035	-3.807 to 11.88	No	ns	0.54	2.444	12
Cont 5W vs. CUMS 3W	10.3	2.453 to 18.14	Yes	**	0.0085	6.236	12
Cont 5W vs. CUMS 5W	17.27	9.425 to 25.11	Yes	****	<0.0001	10.46	12
CUMS 1W vs. CUMS 3W	6.26	-1.582 to 14.10	No	ns	0.1506	3.792	12
CUMS 1W vs. CUMS 5W	13.23	5.390 to 21.07	Yes	**	0.0011	8.015	12
CUMS 3W vs. CUMS 5W	6.972	-0.8700 to 14.81	No	ns	0.0926	4.223	12

The expression of RIP3K is increased in the hippocampal astrocytes of CUMS mice in a time-dependent manner.

D

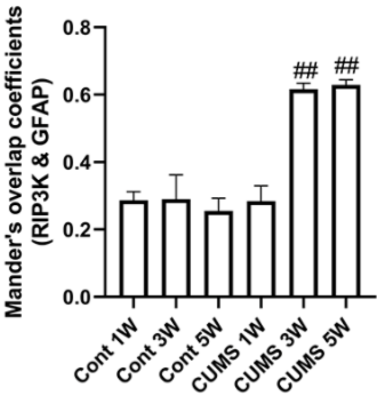

Groups	Cont 1W	Cont 3W	Cont 5W	CUMS 1W	CUMS 3W	CUMS 5W
Mander's overlap coefficients	0.294	0.373	0.232	0.235	0.636	0.618
(RIPK3 & GFAP)	0.308	0.242	0.234	0.293	0.612	0.646
	0.259	0.256	0.299	0.325	0.602	0.624

Tukey's multiple comparisons test	Mean Diff.	95.00% CI of diff.	Below threshold?	Summary	Adjusted P Value	q	DF
Cont 1W vs. Cont 3W	-0.003333	-0.1146 to 0.1079	No	ns	>0.9999	0.1424	12
Cont 1W vs. Cont 5W	0.032	-0.07922 to 0.1432	No	ns	0.9201	1.367	12
Cont 1W vs. CUMS 1W	0.002667	-0.1086 to 0.1139	No	ns	>0.9999	0.1139	12
Cont 1W vs. CUMS 3W	-0.3297	-0.4409 to -0.2184	Yes	****	<0.0001	14.08	12
Cont 1W vs. CUMS 5W	-0.3423	-0.4536 to -0.2311	Yes	****	<0.0001	14.62	12
Cont 3W vs. Cont 5W	0.03533	-0.07589 to 0.1466	No	ns	0.8852	1.509	12
Cont 3W vs. CUMS 1W	0.006	-0.1052 to 0.1172	No	ns	>0.9999	0.2563	12
Cont 3W vs. CUMS 3W	-0.3263	-0.4376 to -0.2151	Yes	****	<0.0001	13.94	12
Cont 3W vs. CUMS 5W	-0.339	-0.4502 to -0.2278	Yes	****	<0.0001	14.48	12
Cont 5W vs. CUMS 1W	-0.02933	-0.1406 to 0.08189	No	ns	0.9429	1.253	12
Cont 5W vs. CUMS 3W	-0.3617	-0.4729 to -0.2504	Yes	****	<0.0001	15.45	12
Cont 5W vs. CUMS 5W	-0.3743	-0.4856 to -0.2631	Yes	****	<0.0001	15.99	12
CUMS 1W vs. CUMS 3W	-0.3323	-0.4436 to -0.2211	Yes	****	<0.0001	14.19	12
CUMS 1W vs. CUMS 5W	-0.345	-0.4562 to -0.2338	Yes	****	<0.0001	14.73	12
CUMS 3W vs. CUMS 5W	-0.01267	-0.1239 to 0.09856	No	ns	0.9986	0.541	12

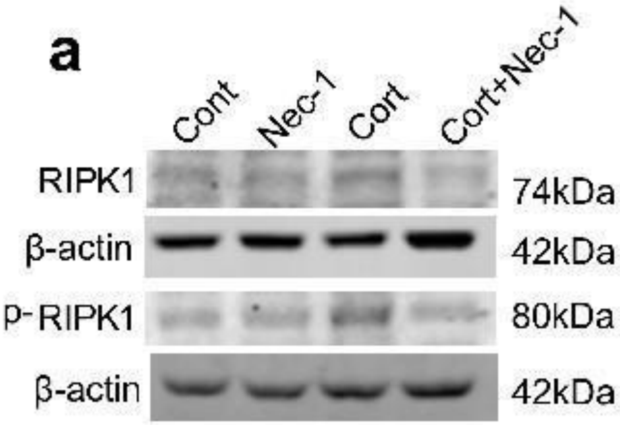

NEC-1 RIP1

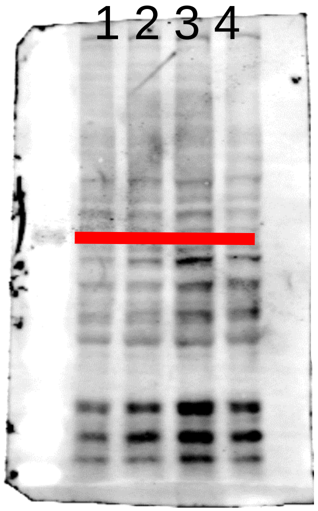

1= Cont
2 = C+Nec 1
3= Cort 200
4=Cort+Nec1

NEC-1 B.A

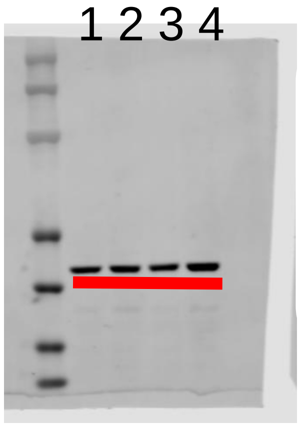

1= Cont
2 = C+Nec 1
3= Cort 200
4=Cort+Nec1

NEC-1 P.RIP1

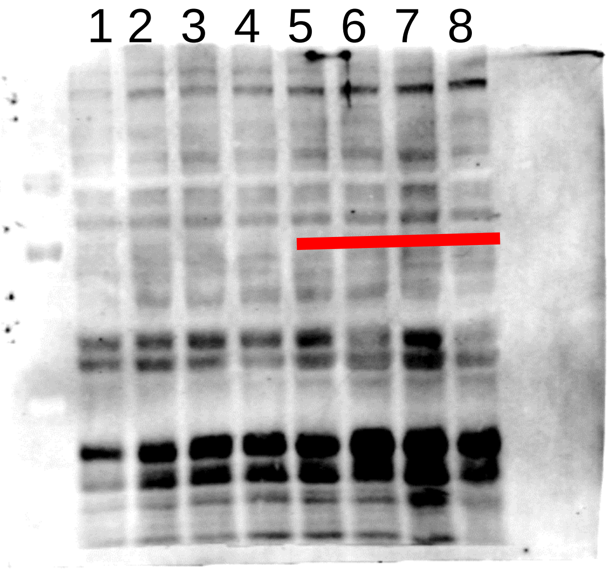

1+5= Cont
2+6= C+Nec 1
3+7= Cort 200
4+8= Cort+Nec1

NEC-1 B.A

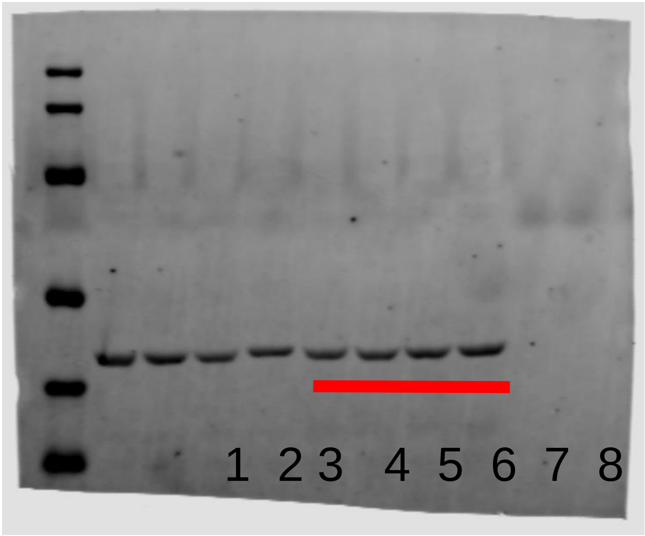

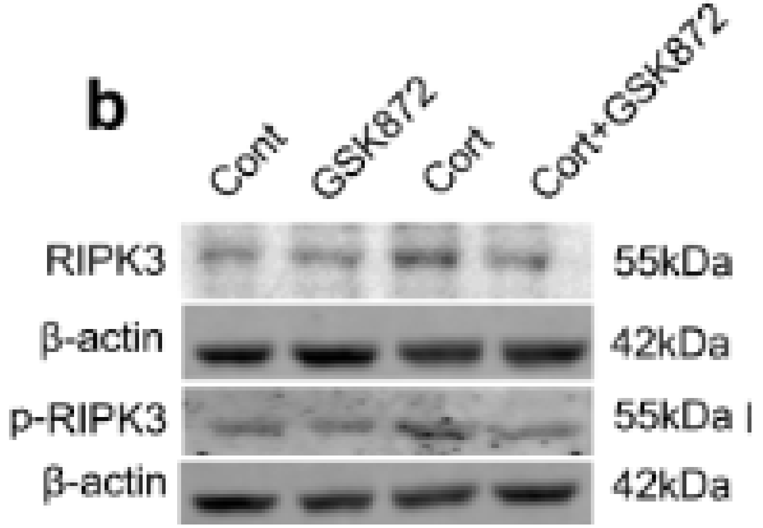

GSK RIP3

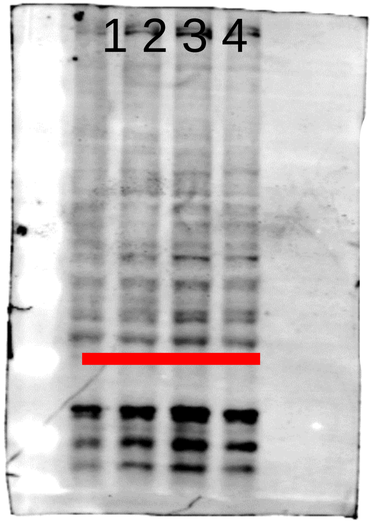

1= Cont
2= C+GSK
3= Cort 200
4= Cort+GSK

GSK B.A

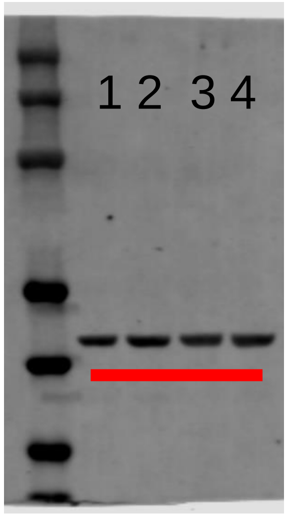

GSK p-RIP3

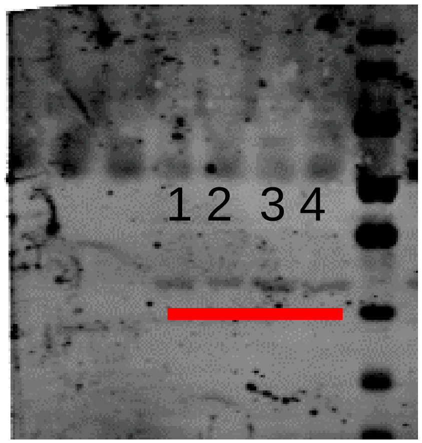

1= Cont
2= C+GSK
3= Cort 200
4=Cort+GSK

GSK B.A

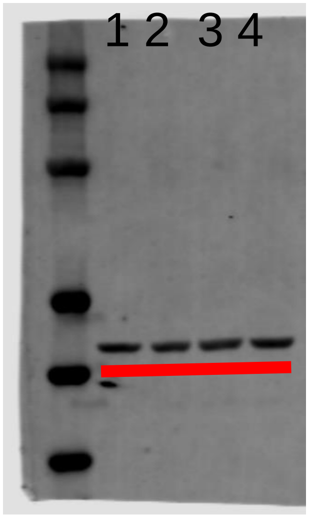

1= Cont
2= C+GSK
3= Cort 200
4=Cort+GSK

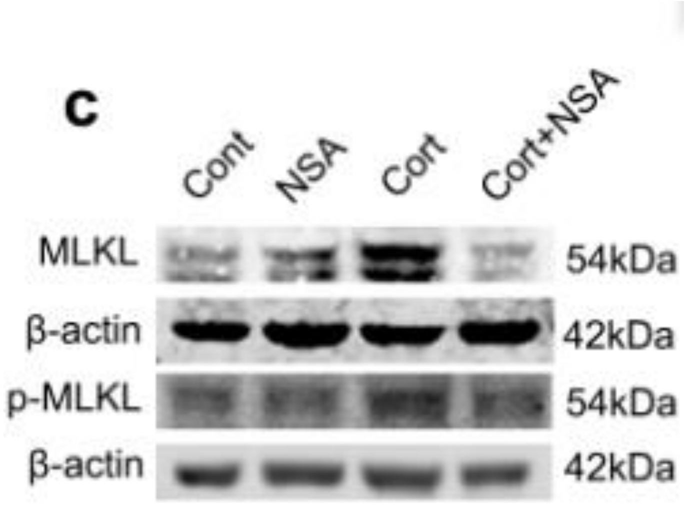

NSA PMLKL

1= Cont
2= C+NSA
3= Cort 200
4= Cort+NSA

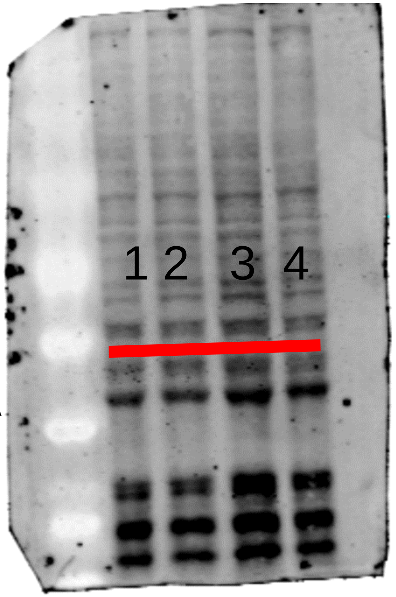

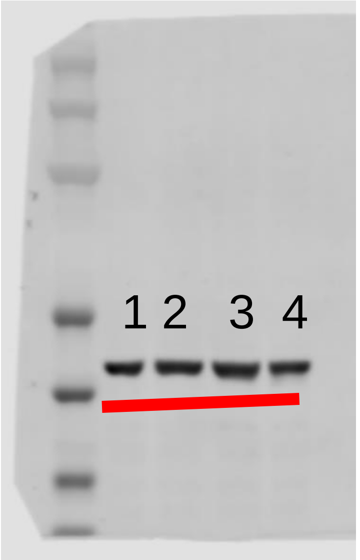

NSA MLKL-1+2

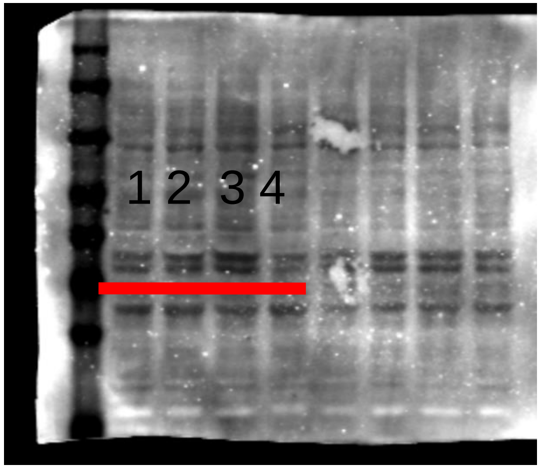

1+5= Cont
2+6= C+NSA
3+7= Cort 200
4+8= Cort+NSA

NSA MLKL-1+2 B.A

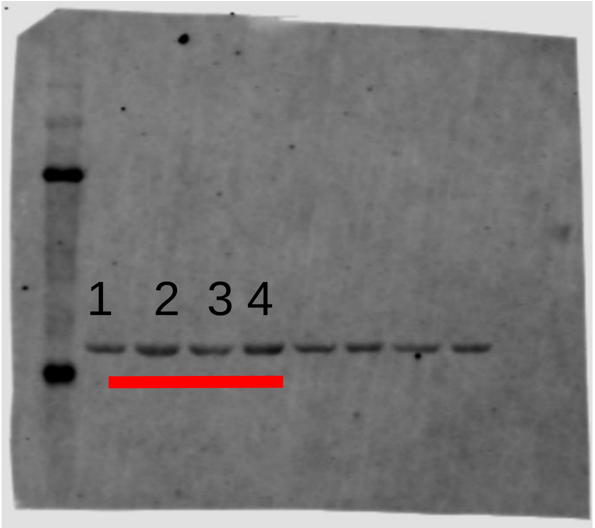

1+5= Cont
2+6= C+NSA
3+7= Cort 200
4+8= Cort+NSA

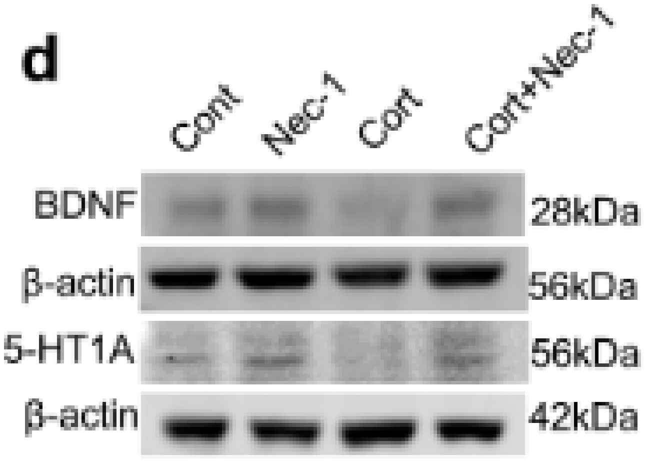

NEC-1 5HT1A-3

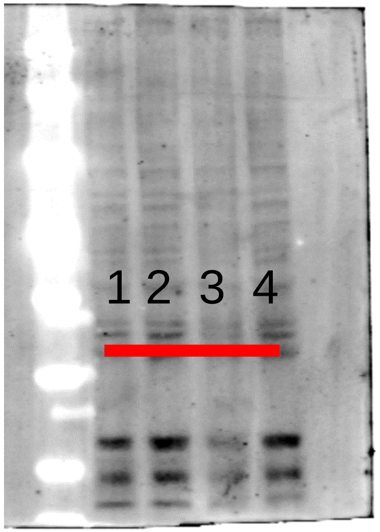

NEC-1 BDNF-3

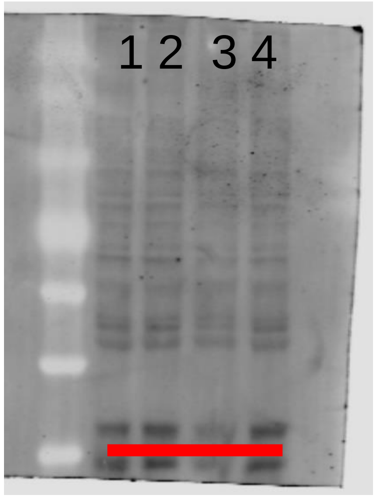

1= Cont
2 = C+Nec 1
3= Cort 200
4=Cort+Nec1

NEC-1 5HT1A-3 B.A

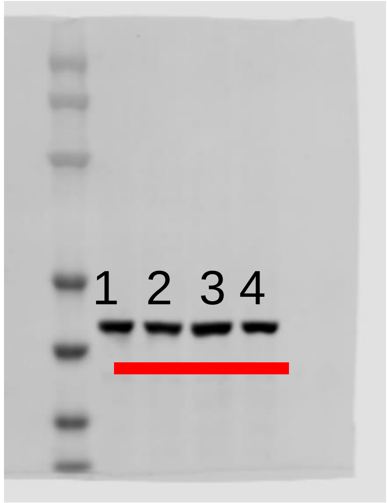

NEC-1 BDNF B.A

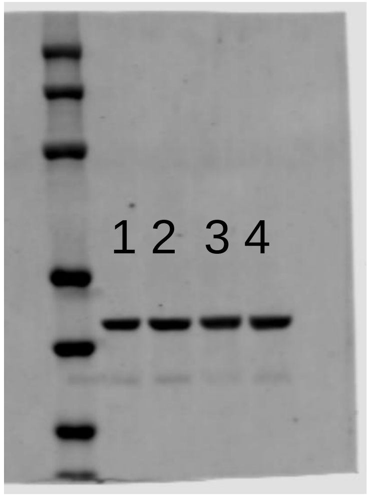

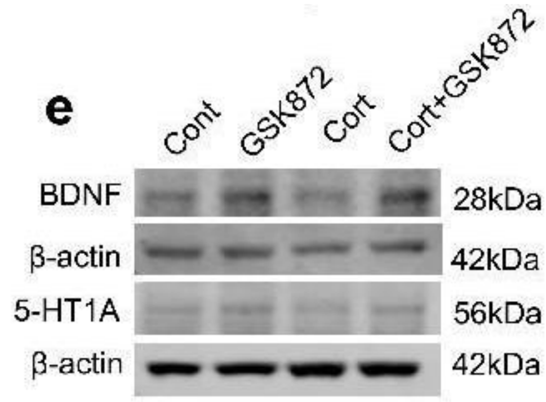

GSK BDNF-1

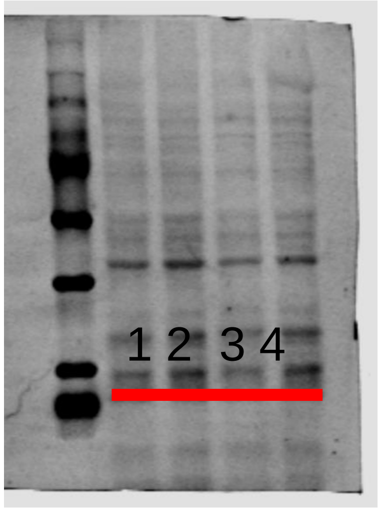

GSK BDNF-3 B.A

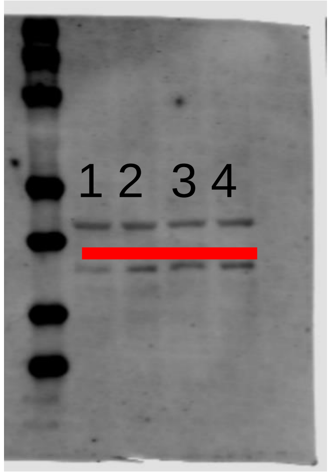

GSK 5HT1A-3 B.A

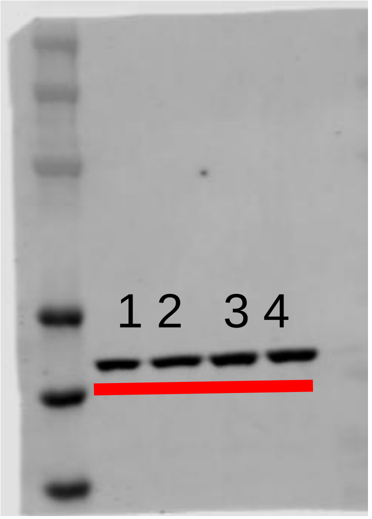

GSK 5HT1A-3

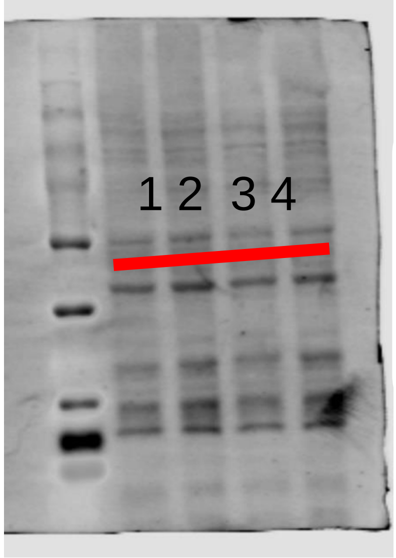

1= Cont
2= C+GSK
3= Cort 200
4= Cort+GSK

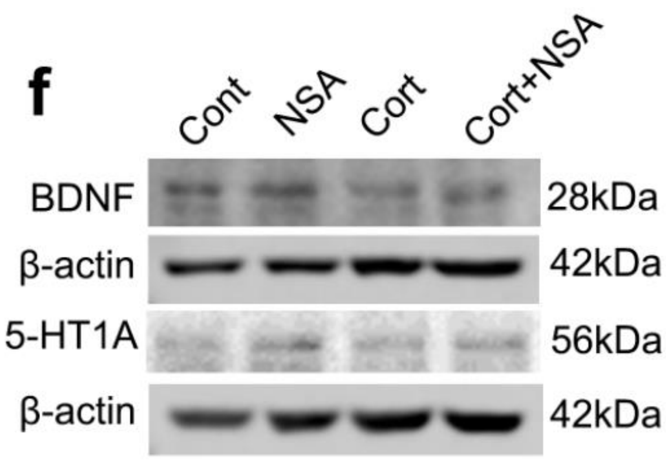

NSA BDNF-3

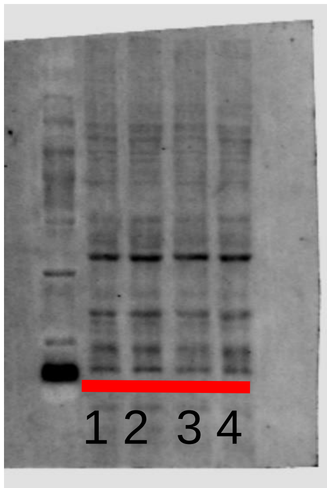

NSA BDNF-3 B.A

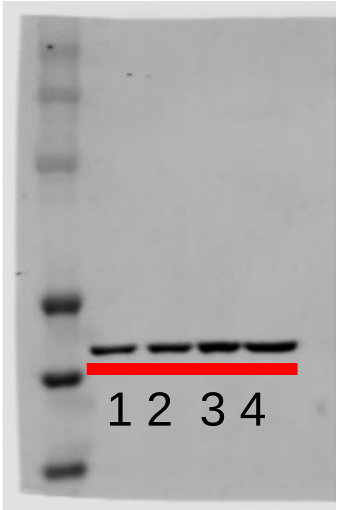

NSA 5HT1A-2

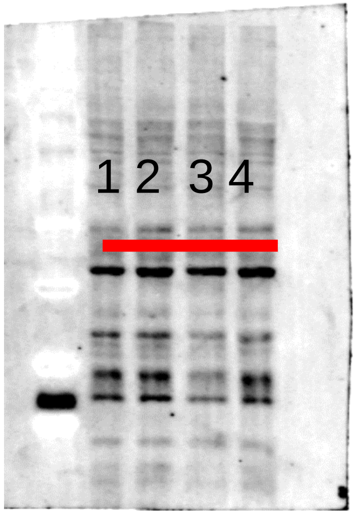

NSA B.A

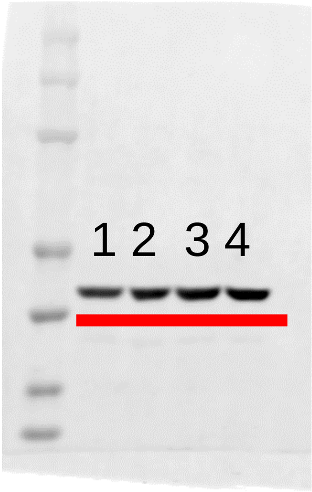

1= Cont
2= C+NSA
3= Cort 200
4= Cort+NSA

Necroptotic kinase inhibitors reduce Cort-induced HA necrosis

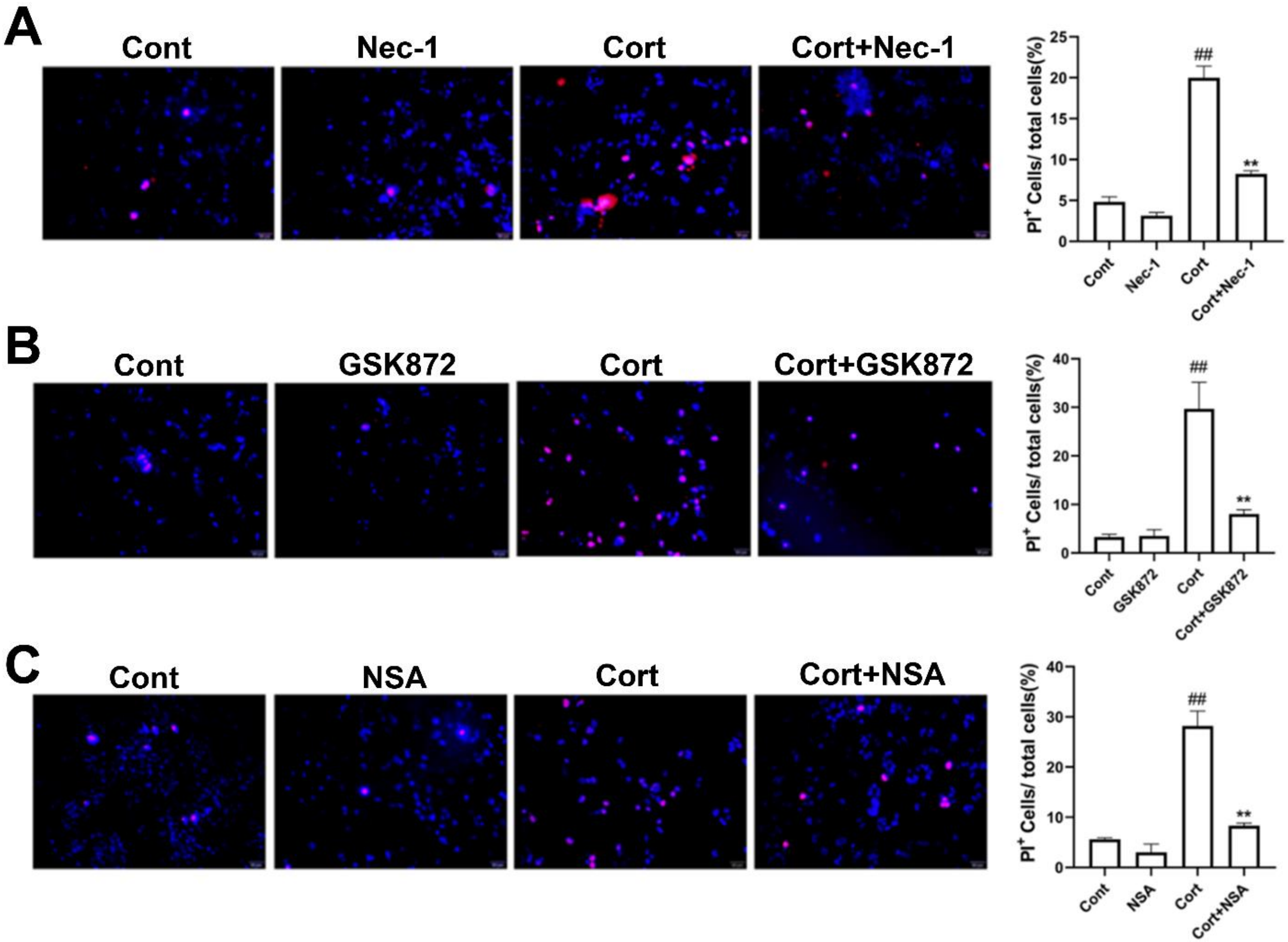

Necroptotic kinase inhibitors reduce Cort-induced HA necrosis

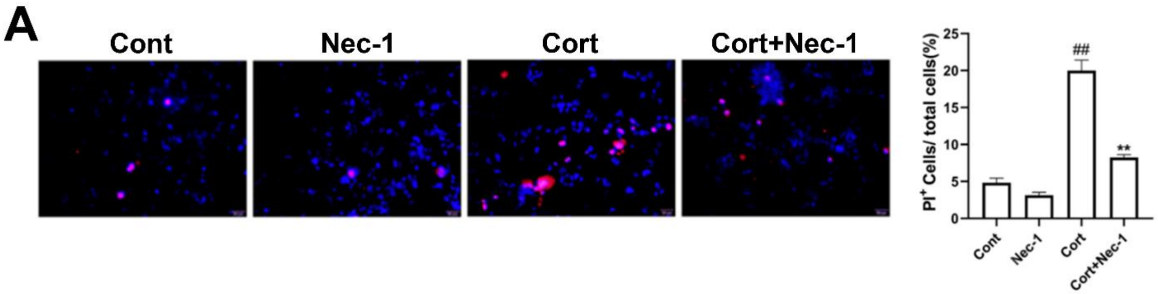

Groups	Cont	Cont+Nec-1	Cort	Cort+Nec-1
Nec-1	4.123711	2.702703	21.15385	8.695652
PI+ Cells/ total cells(%)	5.154639	3.305785	20.38835	8.045977
	5.208333	3.418803	18.42105	7.954545

Tukey's multiple comparisons test	Mean Diff.	95.00% CI of diff.	Below threshold?	Summary	Adjusted P Value	q	DF
Cont vs. Cont+Nec-1	1.686	-0.4508 to 3.824	No	ns	0.1295	3.574	8
Cont vs. Cort	-15.16	-17.30 to -13.02	Yes	****	<0.0001	32.12	8
Cont vs. Cort+Nec-1	-3.403	-5.540 to -1.266	Yes	**	0.0041	7.211	8
Cont+Nec-1 vs. Cort	-16.85	-18.98 to -14.71	Yes	****	<0.0001	35.69	8
Cont+Nec-1 vs. Cort+Nec-1	-5.09	-7.227 to -2.952	Yes	***	0.0003	10.78	8
Cort vs. Cort+Nec-1	11.76	9.618 to 13.89	Yes	****	<0.0001	24.91	8

Necroptotic kinase inhibitors reduce Cort-induced HA necrosis

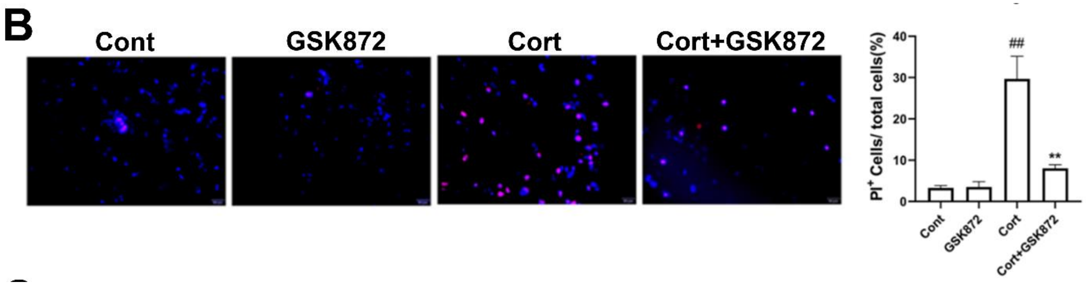

Groups	Cont	Cont+GSK872	Cort	Cort+GSK872
GSK872	2.912621	2.272727	33	8.988764
PI+ Cells/ total cells(%)	2.970297	3.409091	32.67327	7.865169
	3.921569	4.854369	23.40426	7.216495

Tukey's multiple comparisons test	Mean Diff.	95.00% CI of diff.	Below threshold?	Summary	Adjusted P Value	q	DF
Cont vs. Cont+GSK872	-0.2439	-7.695 to 7.207	No	ns	0.9996	0.1482	8
Cont vs. Cort	-26.42	-33.88 to -18.97	Yes	****	<0.0001	16.06	8
Cont vs. Cort+GSK872	-4.755	-12.21 to 2.696	No	ns	0.2495	2.89	8
Cont+GSK872 vs. Cort	-26.18	-33.63 to -18.73	Yes	****	<0.0001	15.91	8
Cont+GSK872 vs. Cort+GSK872	-4.511	-11.96 to 2.940	No	ns	0.2856	2.742	8
Cort vs. Cort+GSK872	21.67	14.22 to 29.12	Yes	****	<0.0001	13.17	8

Necroptotic kinase inhibitors reduce Cort-induced HA necrosis

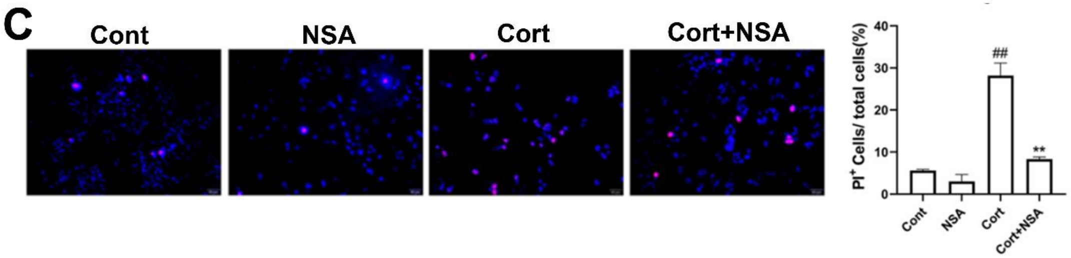

Groups NSA	Cont	Cont+NSA	Cort	Cort+NSA
	5.30303	2.061856	30.85106	7.692308
PI+ Cells/ total cells(%)	5.737705	2.040816	28.7234	8.653846
	5.785124	4.901961	25	8.571429

Tukey's multiple comparisons test	Mean Diff.	95.00% CI of diff.	Below threshold?	Summary	Adjusted P Value	q	DF
Cont vs. Cont+NSA	2.607	-1.890 to 7.105	No	ns	0.3169	2.625	8
Cont vs. Cort	-22.58	-27.08 to -18.09	Yes	****	<0.0001	22.74	8
Cont vs. Cort+NSA	-2.697	-7.195 to 1.800	No	ns	0.2923	2.716	8
Cont+NSA vs. Cort	-25.19	-29.69 to -20.69	Yes	****	<0.0001	25.37	8
Cont+NSA vs. Cort+NSA	-5.304	-9.802 to -0.8069	Yes	*	0.0225	5.341	8
Cort vs. Cort+NSA	19.89	15.39 to 24.38	Yes	****	<0.0001	20.02	8

Necroptotic kinase inhibitors reduce Cort-induced HA necrosis

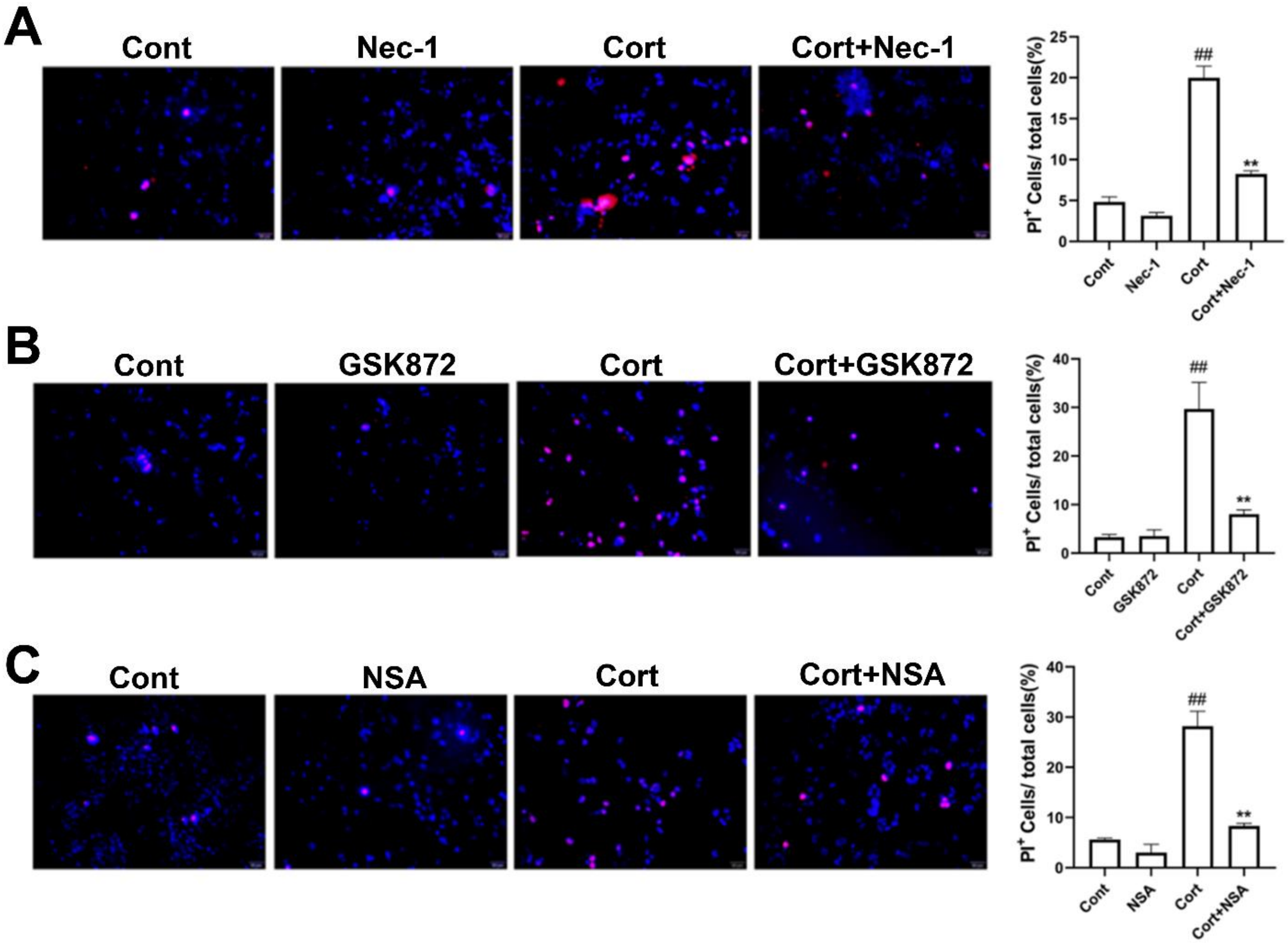

Necroptotic kinase inhibitors reduce Cort-induced HA necrosis

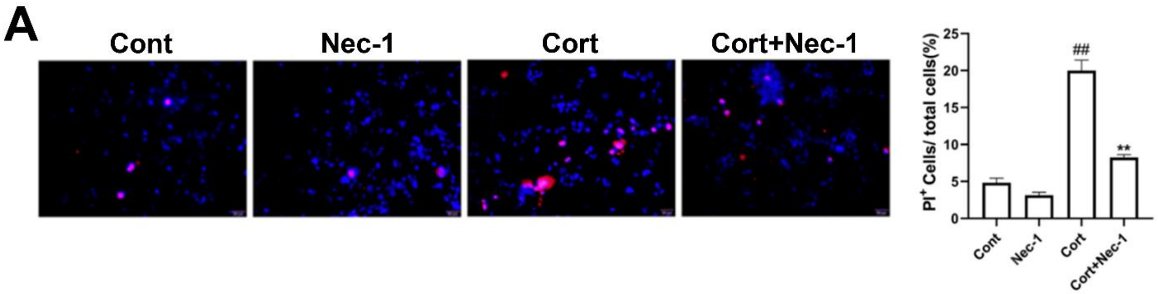

Groups	Cont	Cont+Nec-1	Cort	Cort+Nec-1
Nec-1	4.123711	2.702703	21.15385	8.695652
PI+ Cells/ total cells(%)	5.154639	3.305785	20.38835	8.045977
	5.208333	3.418803	18.42105	7.954545

Tukey's multiple comparisons test	Mean Diff.	95.00% CI of diff.	Below threshold?	Summary	Adjusted P Value	q	DF
Cont vs. Cont+Nec-1	1.686	-0.4508 to 3.824	No	ns	0.1295	3.574	8
Cont vs. Cort	-15.16	-17.30 to -13.02	Yes	****	<0.0001	32.12	8
Cont vs. Cort+Nec-1	-3.403	-5.540 to -1.266	Yes	**	0.0041	7.211	8
Cont+Nec-1 vs. Cort	-16.85	-18.98 to -14.71	Yes	****	<0.0001	35.69	8
Cont+Nec-1 vs. Cort+Nec-1	-5.09	-7.227 to -2.952	Yes	***	0.0003	10.78	8
Cort vs. Cort+Nec-1	11.76	9.618 to 13.89	Yes	****	<0.0001	24.91	8

Necroptotic kinase inhibitors reduce Cort-induced HA necrosis

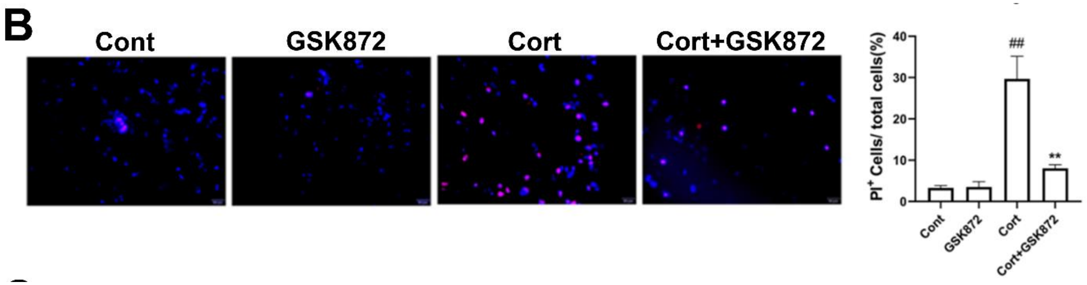

Groups	Cont	Cont+GSK872	Cort	Cort+GSK872
GSK872	2.912621	2.272727	33	8.988764
PI+ Cells/ total cells(%)	2.970297	3.409091	32.67327	7.865169
	3.921569	4.854369	23.40426	7.216495

Tukey's multiple comparisons test	Mean Diff.	95.00% CI of diff.	Below threshold?	Summary	Adjusted P Value	q	DF
Cont vs. Cont+GSK872	-0.2439	-7.695 to 7.207	No	ns	0.9996	0.1482	8
Cont vs. Cort	-26.42	-33.88 to -18.97	Yes	****	<0.0001	16.06	8
Cont vs. Cort+GSK872	-4.755	-12.21 to 2.696	No	ns	0.2495	2.89	8
Cont+GSK872 vs. Cort	-26.18	-33.63 to -18.73	Yes	****	<0.0001	15.91	8
Cont+GSK872 vs. Cort+GSK872	-4.511	-11.96 to 2.940	No	ns	0.2856	2.742	8
Cort vs. Cort+GSK872	21.67	14.22 to 29.12	Yes	****	<0.0001	13.17	8

Necroptotic kinase inhibitors reduce Cort-induced HA necrosis

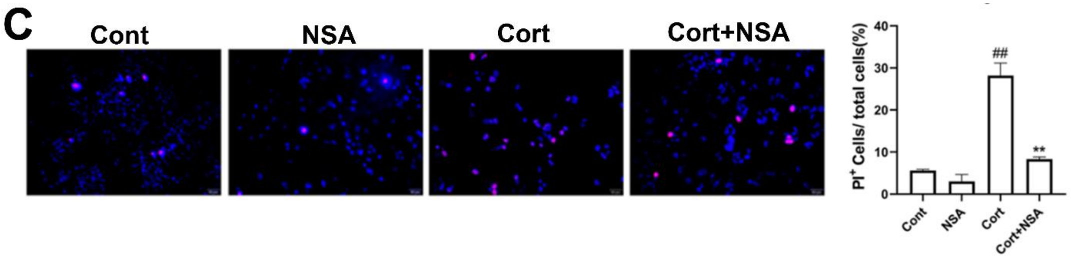

Groups	Cont	Cont+NSA	Cort	Cort+NSA
NSA	5.30303	2.061856	30.85106	7.692308
PI+ Cells/ total cells(%)	5.737705	2.040816	28.7234	8.653846
	5.785124	4.901961	25	8.571429

Tukey's multiple comparisons test	Mean Diff.	95.00% CI of diff.	Below threshold?	Summary	Adjusted P Value	q	DF
Cont vs. Cont+NSA	2.607	-1.890 to 7.105	No	ns	0.3169	2.625	8
Cont vs. Cort	-22.58	-27.08 to -18.09	Yes	****	<0.0001	22.74	8
Cont vs. Cort+NSA	-2.697	-7.195 to 1.800	No	ns	0.2923	2.716	8
Cont+NSA vs. Cort	-25.19	-29.69 to -20.69	Yes	****	<0.0001	25.37	8
Cont+NSA vs. Cort+NSA	-5.304	-9.802 to -0.8069	Yes	*	0.0225	5.341	8
Cort vs. Cort+NSA	19.89	15.39 to 24.38	Yes	****	<0.0001	20.02	8

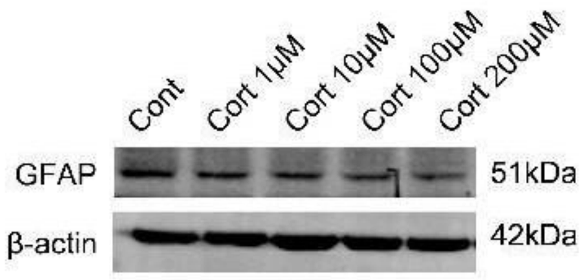

1 = Cont
2 = Cort 1
3= Cort 10
4= Cort 100
5= Cort 200

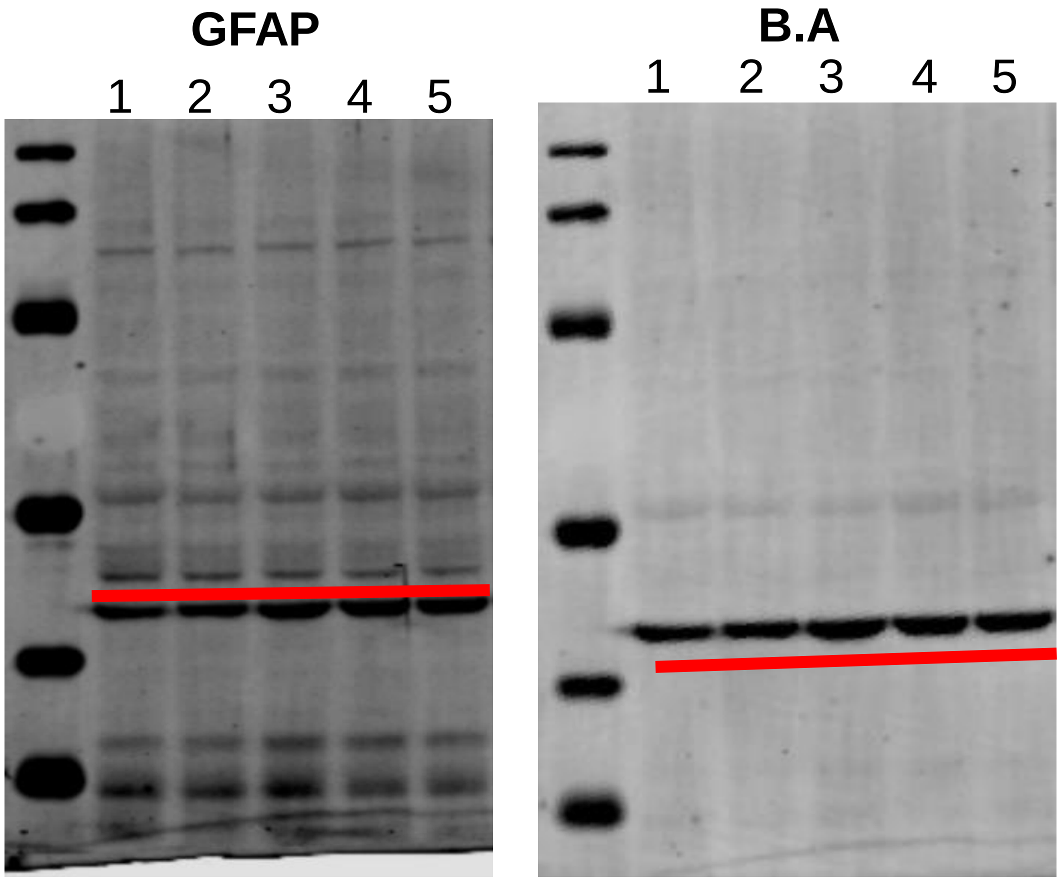

Fluoxetine-treatment ameliorates the depressive-like behaviours in the CUMS-induced mice

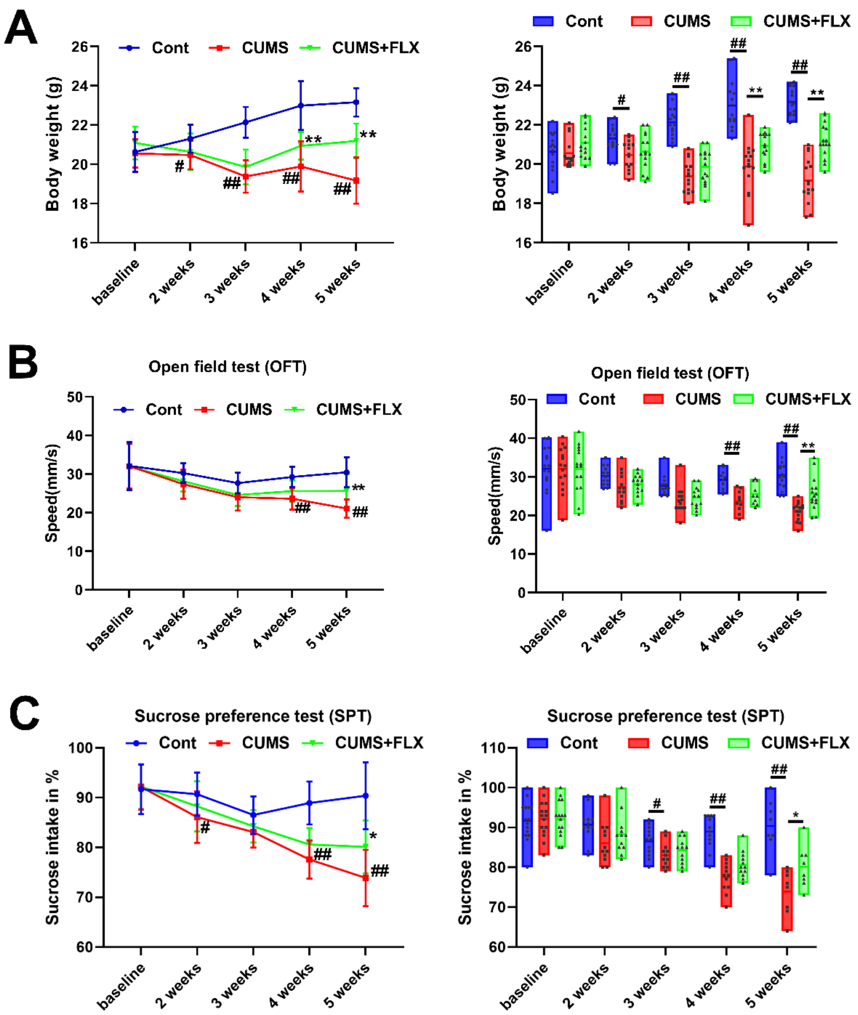

Fluoxetine-treatment ameliorates the depressive-like behaviours in the CUMS-induced mice

Body Weight	Cont															
	baseline	21.2	19.7	19.1	20.9	21	19.9	21.6	18.5	20.1	20.5	22.2	20.7	21.5	21.6	20.9
	2 weeks	21.8	21.5	22.3	21.5	22	21.1	21	21.7	21.3	21	20.9	22.4	20	21	20
	3 weeks	21.8	22.8	22.3	21.5	22	21.1	22.5	21.7	21.3	23.3	20.9	22.4	23.6	22	22.8
	4 weeks	23.6	23.3	22.3	22.2	21.7	22.5	21.3	21.9	22.2	25.3	23	23.7	22.3	24.1	25.4
	5 weeks	23.2	23	23.1	24	22.5	22.1	22.6	22.7	24.1	24.2	22.5	22.2	24	23.7	23.5
	CUMS															
	baseline	22.1	21.7	20	20	20.3	19.9	20.4	20.9	20.1	20.3	20.4	21.8	20.2	20.3	19.9
	2 weeks	20.5	20	19.9	21.4	21	19.5	21	20.4	21.2	19.7	19.2	21.5	20.8	21	20
	3 weeks	20.5	19	19.9	19.4	18	19.5	18.5	20.4	19.9	19.7	19.2	18.6	20.8	18.6	18.7
	4 weeks	20.2	20.8	19.9	19.8	19.4	19.8	16.9	18.5	22.5	20.7	20.4	20.6	20	18.4	20.4
	5 weeks	17.3	19	18.7	18.5	17.4	18.7	18.6	19.8	20.1	19.9	20.7	19.1	18	21	20.8
	CUMS+FLX															
	baseline	21.1	20.3	20.3	20.4	20.7	21.8	22.1	19.9	20.8	21.4	22.5	20.6			
	2 weeks	19.3	20.5	22	19.1	20.6	21.1	20	21.1	21.5	20.3	19.4	20.5	21.6		
	3 weeks	19.3	20.5	19	19.1	20.6	21.1	20	21.1	18.1	20.3	19.4	20.5	19.5		
	4 weeks	21.5	20.7	21.6	19.6	20	21	21.9	20.9	21.5	20.6	21.5	21.5	20.5		
	5 weeks	22.6	20.6	21.9	20.2	20	19.6	21	22.2	21.8	21.2	21	22.5	21		

Fluoxetine-treatment ameliorates the depressive-like behaviours in the CUMS-induced mice

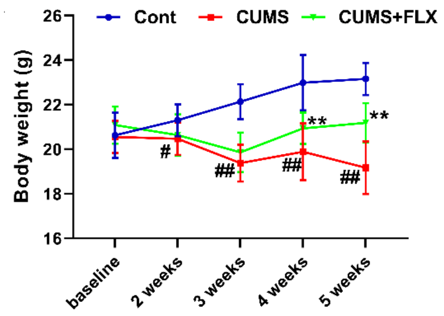

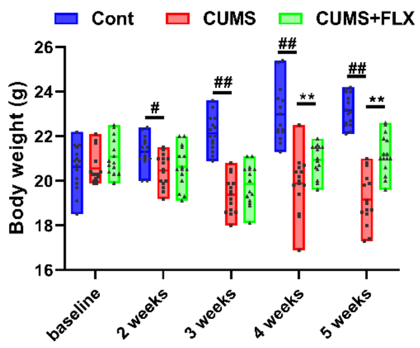

Tukey's multiple comparisons test	Predicted (LS) mean diff.	95.00% CI of diff.	Below threshold?	Summary	Adjusted P Value	q	DF
Baseline							
Cont vs. CUMS	0.07333	-0.7159 to 0.8626	No	ns	0.9738	0.3102	209
Cont vs. CUMS+FLX	-0.459	-1.262 to 0.3442	No	ns	0.3698	1.908	209
CUMS vs. CUMS+FLX	-0.5324	-1.336 to 0.2708	No	ns	0.2633	2.213	209
2 weeks							
Cont vs. CUMS	0.8267	0.03742 to 1.616	Yes	*	0.0377	3.497	209
Cont vs. CUMS+FLX	0.6667	-0.1226 to 1.456	No	ns	0.1162	2.82	209
CUMS vs. CUMS+FLX	-0.16	-0.9492 to 0.6292	No	ns	0.8815	0.6768	209
3 weeks							
Cont vs. CUMS	2.753	1.964 to 3.543	Yes	****	<0.0001	11.65	209
Cont vs. CUMS+FLX	2.273	1.484 to 3.063	Yes	****	<0.0001	9.616	209
CUMS vs. CUMS+FLX	-0.48	-1.269 to 0.3092	No	ns	0.3245	2.03	209
4 weeks							
Cont vs. CUMS	3.1	2.311 to 3.889	Yes	****	<0.0001	13.11	209
Cont vs. CUMS+FLX	2.047	1.257 to 2.836	Yes	****	<0.0001	8.657	209
CUMS vs. CUMS+FLX	-1.053	-1.843 to -0.2641	Yes	**	0.0053	4.455	209
5 weeks							
Cont vs. CUMS	3.987	3.197 to 4.776	Yes	****	<0.0001	16.86	209
Cont vs. CUMS+FLX	1.973	1.184 to 2.763	Yes	****	<0.0001	8.347	209
CUMS vs. CUMS+FLX	-2.013	-2.803 to -1.224	Yes	****	<0.0001	8.516	209

Fluoxetine-treatment ameliorates the depressive-like behaviours in the CUMS-induced mice

OFT	Cont														
baseline	40.18	39.97	37.51	37.4	35.25	34.75	32.89	32.85	31.62	31.44	29.83	29.17	26.66	25.82	16.02
2 weeks	32	27	31	29	26.82	28	30	28	31	33	29	35	33		
3 weeks	28	26	27	25	26.82	25.44	30	28	27	27	29	35	25		
4 weeks	26	29.2	26.82	25.44	33.1	29.8	31.05	30.25	28.19	32.6					
5 weeks	26.02	29.09	30.67	35	38.97	25.01	27.66	24.97	32.79	28	32.69	33.74	29.94	30	32.21
	CUMS														
baseline	40.44	38.53	37.57	37.12	35.44	34.71	33.14	32.82	31.68	30.19	29.88	28.13	26.73	25.4	18.74
2 weeks	27	30	26	28	31	22	26	35	25	27	23	23.6	32		
3 weeks	25	22	26	18	22	22	23	33	25	23.97	26	23.6	22		
4 weeks	19	20.28	24.68	26	27.13	23	21.72	23.97	22.81	27.6					
5 weeks	20.09	23	18	19	22.5	22.3	23.7	21	15.99	21	25	18.24	22	21.9	22
	CUMS+FLX														
baseline	41.79	38.52	37.67	37	36.23	33.34	33.31	32.78	32.63	30.12	30.03	27.15	26.99	21.76	20.23
2 weeks	30	28	22.76	27	30	29	26.45	29	26	25.01	29	32	31		
3 weeks	26	23	22.76	20	25	27	26.45	22	23	25.01	29	29	21		
4 weeks	22.76	29	25	23.8	26.45	29.53	23	25.01	22	29					
5 weeks	25	23.33	26	23.5	29.1	24.27	27.11	19.39	24	22.07	25.59	19.48	27	33.49	35

Fluoxetine-treatment ameliorates the depressive-like behaviours in the CUMS-induced mice

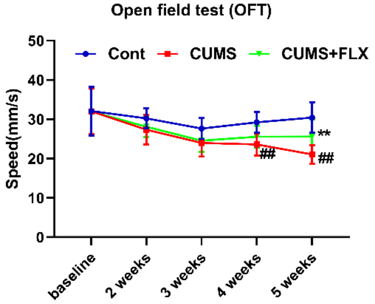

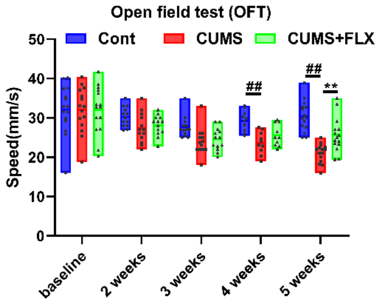

Tukey's multiple comparisons test	Predicted (LS) mean diff.	95.00% CI of diff.	Below threshold?	Summary	Adjusted P Value	q	DF
Baseline							
Cont vs. CUMS	0.056	-3.409 to 3.521	No	ns	0.9992	0.054	183
Cont vs. CUMS+FLX	0.1207	-3.345 to 3.586	No	ns	0.9963	0.1164	183
CUMS vs. CUMS+FLX	0.06467	-3.401 to 3.530	No	ns	0.9989	0.06236	183
2 weeks							
Cont vs. CUMS	2.863	-0.8593 to 6.585	No	ns	0.1667	2.57	183
Cont vs. CUMS+FLX	2.123	-1.599 to 5.845	No	ns	0.3707	1.906	183
CUMS vs. CUMS+FLX	-0.74	-4.462 to 2.982	No	ns	0.8856	0.6643	183
3 weeks							
Cont vs. CUMS	3.668	-0.05392 to 7.391	No	ns	0.0544	3.293	183
Cont vs. CUMS+FLX	3.08	-0.6424 to 6.802	No	ns	0.1264	2.765	183
CUMS vs. CUMS+FLX	-0.5885	-4.311 to 3.134	No	ns	0.926	0.5283	183
4 weeks							
Cont vs. CUMS	5.626	1.382 to 9.870	Yes	**	0.0057	4.43	183
Cont vs. CUMS+FLX	3.69	-0.5542 to 7.934	No	ns	0.1024	2.905	183
CUMS vs. CUMS+FLX	-1.936	-6.180 to 2.308	No	ns	0.529	1.524	183
5 weeks							
Cont vs. CUMS	9.403	5.937 to 12.87	Yes	****	<0.0001	9.067	183
Cont vs. CUMS+FLX	4.829	1.363 to 8.294	Yes	**	0.0034	4.656	183
CUMS vs. CUMS+FLX	-4.574	-8.039 to -1.109	Yes	**	0.006	4.411	183

Fluoxetine-treatment ameliorates the depressive-like behaviours in the CUMS-induced mice

SPT					Cont										
baseline	100	98	95	95	95	95	92	92	91	90	89	88	88	87	80
2 weeks	90	89	83	91	97	84	92	92	98	90	92	90			
3 weeks	90	89	83	91	87	84	82	92	88	80	85	87			
4 weeks	86	89	83	90	87	93	88	92	93	80	93	93			
5 weeks	88	78	100	88	92	94	96	87							
			CUMS												
baseline	100	98	96	96	94	94	93	93	92	90	90	89	88	86	83
2 weeks	90	89	85	80	90	80	98	84	83	84	82	88			
3 weeks	82	89	85	80	81	80	79	84	83	84	82	88			
4 weeks	78	73	78	70	75	80	79	83	75	81	82	77			
5 weeks	70	78	75	79	80	64	76	69							
			CUMS+FLX												
baseline	100	98	97	97	93	93	93	93	92	90	90	89	88	85	85
2 weeks	88	83	85	82	95	89	86	88	85	88	90	100			
3 weeks	88	83	85	82	85	89	86	81	85	88	79	80			
4 weeks	81	83	77	82	79	76	78	88	80	84	79	80			
5 weeks	76	83	81	90	73	78	77	83							

Fluoxetine-treatment ameliorates the depressive-like behaviours in the CUMS-induced mice

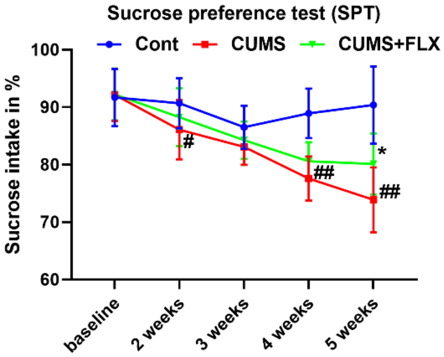

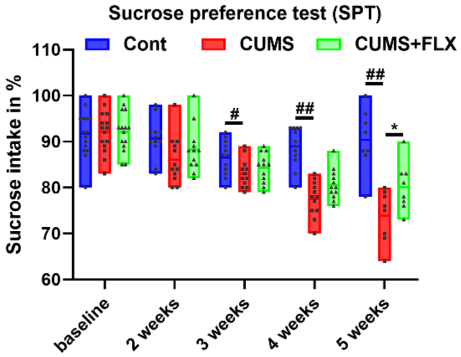

Tukey's multiple comparisons test	Predicted (LS) mean diff.	95.00% CI of diff.	Below threshold?	Summary	Adjusted P Value	q	DF
Baseline							
Cont vs. CUMS	-0.4667	-4.371 to 3.438	No	ns	0.9569	0.3999	162
Cont vs. CUMS+FLX	-0.5333	-4.438 to 3.371	No	ns	0.9441	0.457	162
CUMS vs. CUMS+FLX	-0.06667	-3.971 to 3.838	No	ns	0.9991	0.05712	162
2 weeks							
Cont vs. CUMS	4.583	0.2183 to 8.948	Yes	*	0.0371	3.513	162
Cont vs. CUMS+FLX	2.417	-1.948 to 6.782	No	ns	0.3919	1.852	162
CUMS vs. CUMS+FLX	-2.167	-6.532 to 2.198	No	ns	0.4703	1.66	162
3 weeks							
Cont vs. CUMS	3.417	-0.9483 to 7.782	No	ns	0.1563	2.618	162
Cont vs. CUMS+FLX	2.25	-2.115 to 6.615	No	ns	0.4435	1.724	162
CUMS vs. CUMS+FLX	-1.167	-5.532 to 3.198	No	ns	0.8026	0.8941	162
4 weeks							
Cont vs. CUMS	11.33	6.968 to 15.70	Yes	****	<0.0001	8.686	162
Cont vs. CUMS+FLX	8.333	3.968 to 12.70	Yes	****	<0.0001	6.387	162
CUMS vs. CUMS+FLX	-3	-7.365 to 1.365	No	ns	0.2377	2.299	162
5 weeks							
Cont vs. CUMS	16.5	11.15 to 21.85	Yes	****	<0.0001	10.32	162
Cont vs. CUMS+FLX	10.25	4.904 to 15.60	Yes	****	<0.0001	6.414	162
CUMS vs. CUMS+FLX	-6.25	-11.60 to -0.9040	Yes	*	0.0174	3.911	162

Fluoxetine decreases the immobility time of CUMS mice in FST and TST

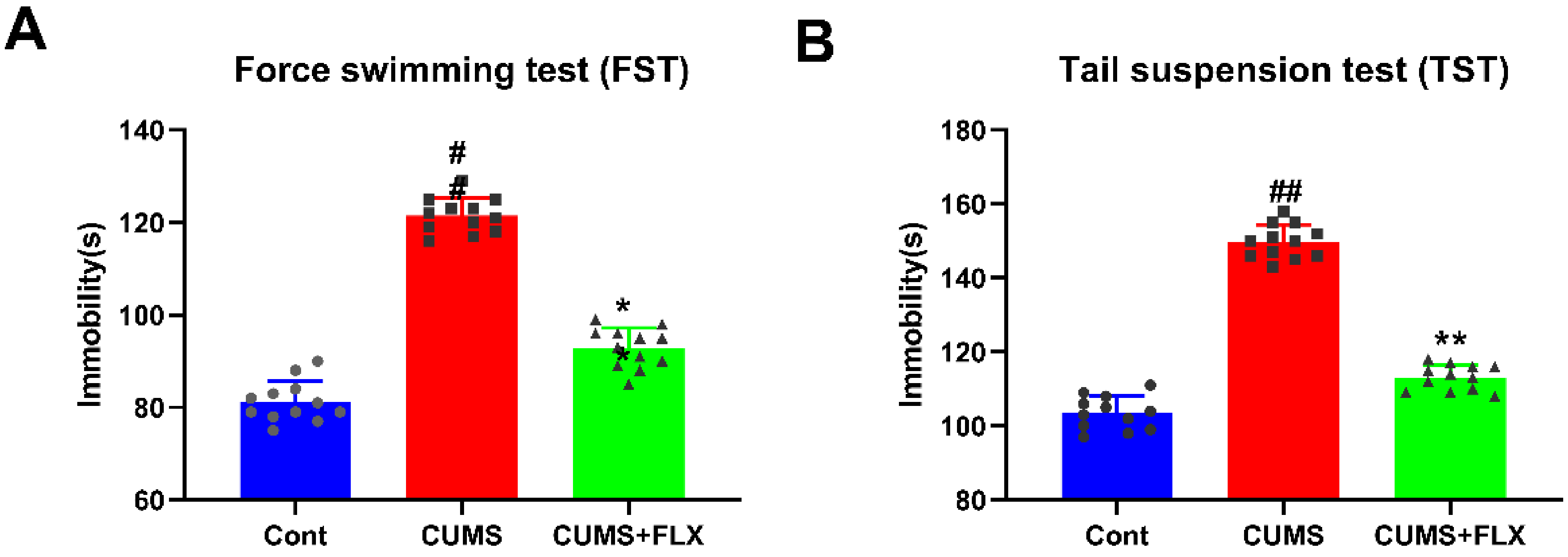

Fluoxetine decreases the immobility time of CUMS mice in Force swimming test

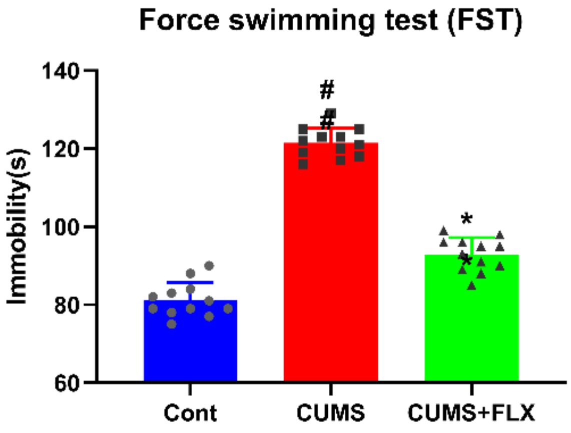

FST											
Cont	84	79	81	88	90	79	78	75	77	79	83	82
CUMS	125	116	123	122	125	117	120	121	119	123	118	129
CUMS+FLX	95	89	91	96	88	98	96	99	95	93	85	90

Tukey's multiple comparisons test	Mean Diff.	95.00% CI of diff.	Below threshold?	Summary	Adjusted P Value			
Cont vs. CUMS	-40.25	-44.44 to -36.06	Yes	****	<0.0001	A-B		
Cont vs. CUMS+FLX	-11.67	-15.86 to -7.474	Yes	****	<0.0001	A-C		
CUMS vs. CUMS+FLX	28.58	24.39 to 32.78	Yes	****	<0.0001	B-C		
Test details	Mean 1	Mean 2	Mean Diff.	SE of diff.	n1	n2	q	DF
Cont vs. CUMS	81.25	121.5	-40.25	1.709	12	12	33.31	33
Cont vs. CUMS+FLX	81.25	92.92	-11.67	1.709	12	12	9.655	33
CUMS vs. CUMS+FLX	121.5	92.92	28.58	1.709	12	12	23.66	33

Fluoxetine decreases the immobility time of CUMS mice in tail suspension test

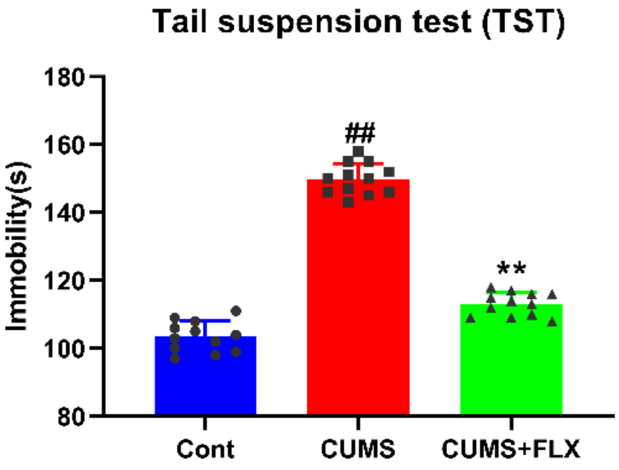

TST												
Cont	103	99	105	102	100	98	108	97	104	109	111	106
CUMS	155	143	145	147	146	150	151	158	152	146	155	150
CUMS+FLX	115	112	110	109	118	116	108	113	116	114	109	117

Tukey's multiple comparisons test	Mean Diff.	95.00% CI of diff.	Below threshold?	Summary	Adjusted P Value			
Cont vs. CUMS	-46.33	-50.56 to -42.11	Yes	****	<0.0001	A-B		
Cont vs. CUMS+FLX	-9.583	-13.81 to -5.356	Yes	****	<0.0001	A-C		
CUMS vs. CUMS+FLX	36.75	32.52 to 40.98	Yes	****	<0.0001	B-C		
Test details	Mean 1	Mean 2	Mean Diff.	SE of diff.	n1	n2	q	DF
Cont vs. CUMS	103.5	149.8	-46.33	1.723	12	12	38.04	33
Cont vs. CUMS+FLX	103.5	113.1	-9.583	1.723	12	12	7.867	33
CUMS vs. CUMS+FLX	149.8	113.1	36.75	1.723	12	12	30.17	33

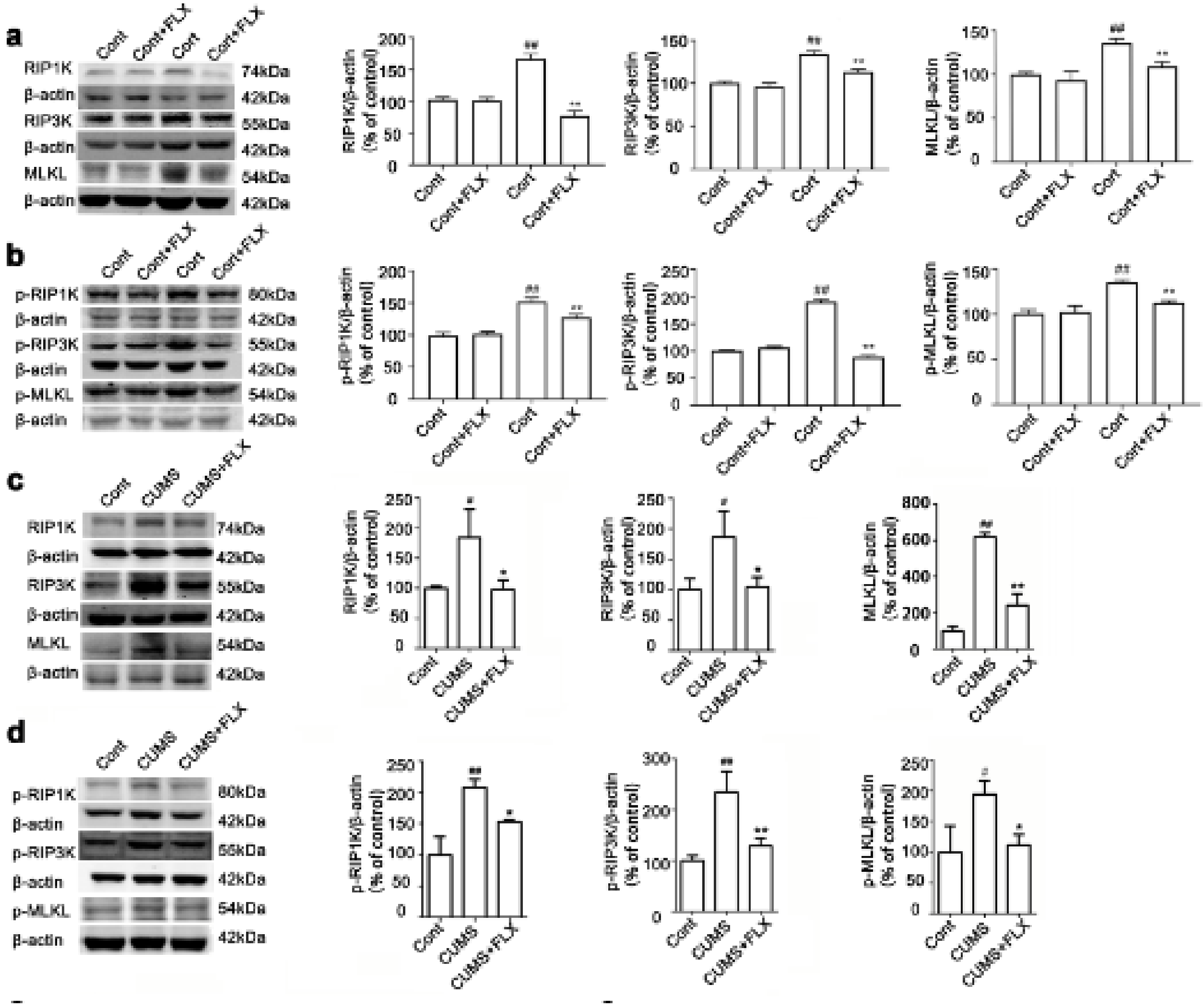
a
b
c
d
Cont
Cont+FLX
Cort
Cort+FLX
CUMS
CUMS+FLX
RIP1K
β-actin
RIP3K
MLKL
p-RIP1K
p-RIP3K
p-MLKL
74kDa
42kDa
55kDa
54kDa
80kDa
RIP1K/β-actin (% of control)
RIP3K/β-actin (% of control)
MLKL/β-actin (% of control)
p-RIP1K/β-actin (% of control)
p-RIP3K/β-actin (% of control)
p-MLKL/β-actin (% of control)

Fluoxetine decreases the expression of necroptosis kinases in human astrocytes.

FLX
RIP1-1

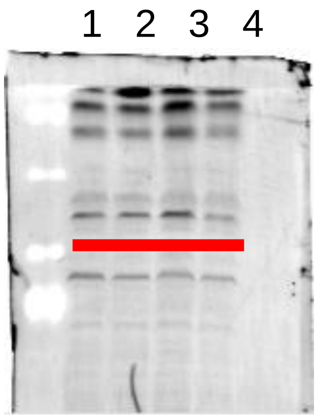

1= Cont
2= C+FLX
3= Cort 200
4= Cort+FLX

FLX RIP1-1
B.A

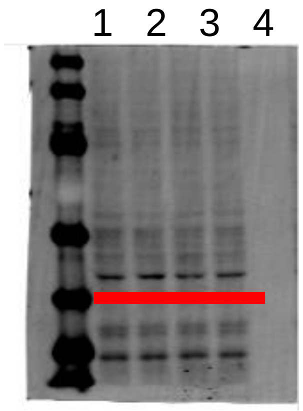

Fluoxetine decreases the expression of necroptosis kinases in human astrocytes.

FLX
P.RIP1-1

1 2 3 4 5 6 7 8

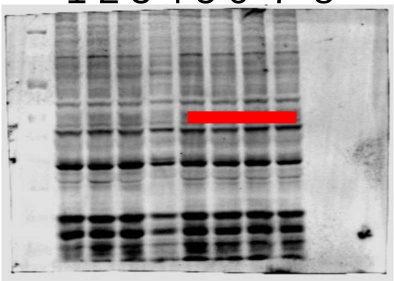

1+5= Cont
2+6= C+FLX
3+7= Cort 200
4+8= Cort+FLX

FLX CELL P.RIP1-1 B.A

1 2 3 4 5 6 7 8

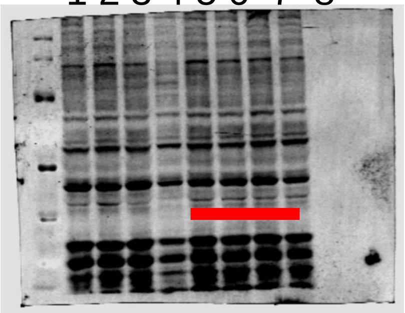

Fluoxetine decreases the expression of necroptosis kinases in human astrocytes.

FLX RIP3-1

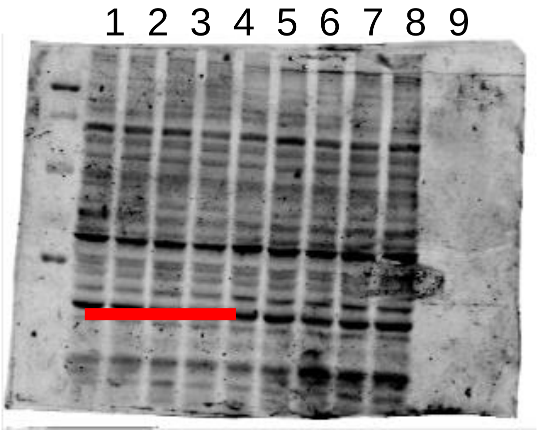

1+5= Cont
2+6= C+FLX
3+7= Cort 200
4+8+9= Cort+FLX

FLX CELL RIP3-1 B.A

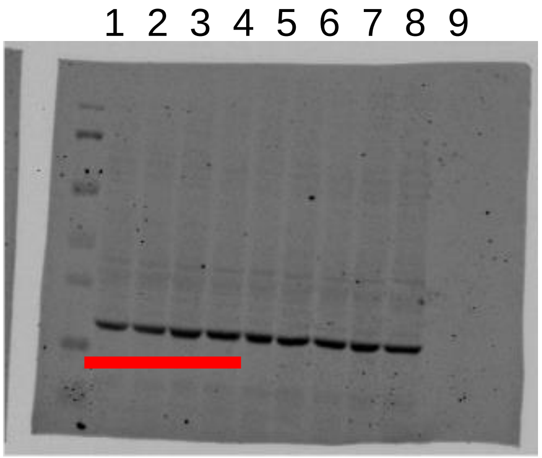

Fluoxetine decreases the expression of necroptosis kinases in human astrocytes.

FLX P.RIP3-1+2

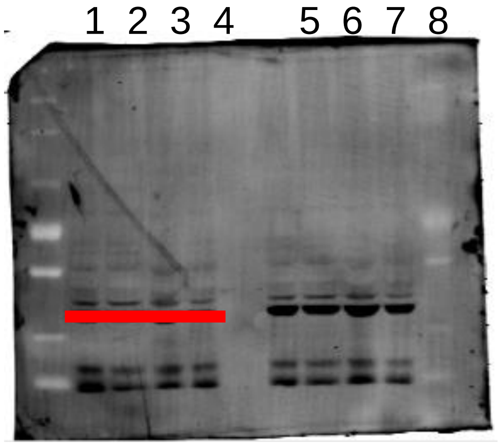

1+5= Cont
2+6= C+FLX
3+7= Cort 200
4+8= Cort+FLX

FLX P.RIP3-1+2 B.A

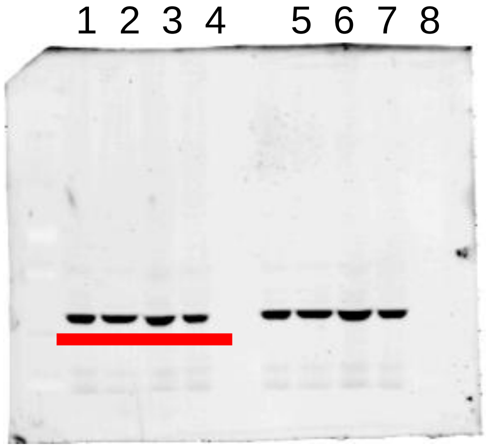

Fluoxetine decreases the expression of necroptosis kinases in human astrocytes.

FLX MLKL-
1+2

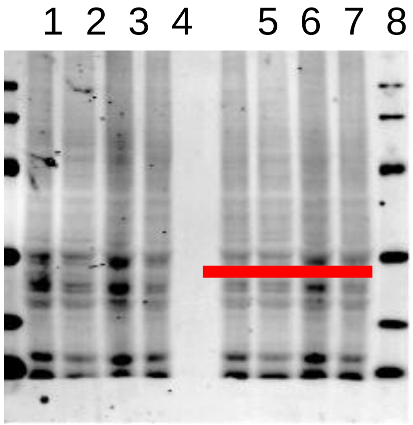

1+5= Cont
2+6= C+FLX
3+7= Cort 200
4+8= Cort+FLX

FLX MLKL1+2
B.A

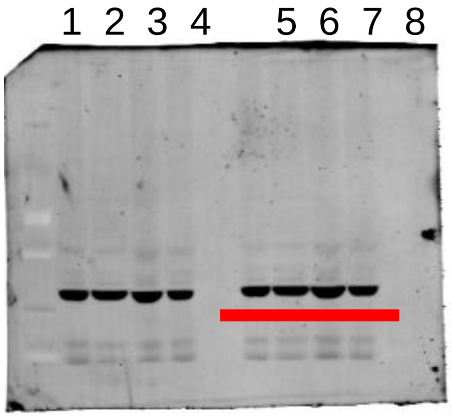

Fluoxetine decreases the expression of necroptosis kinases in human astrocytes.

FLX PMLKL-1+2

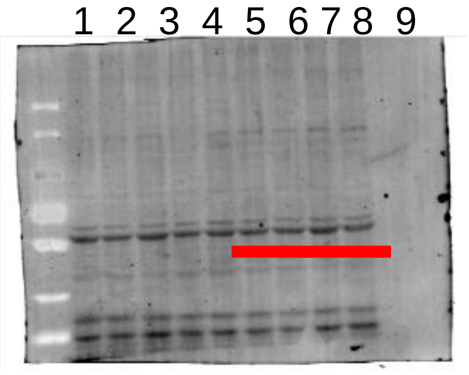

1+6= Cont
2+7= C+FLX
3+8= Cort 200
4+5+9= Cort+FLX

FLX PMLKL-1+2 B.A

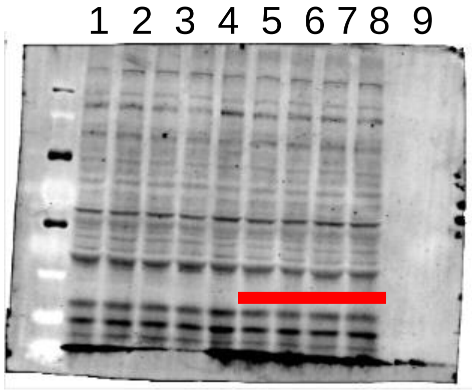

Fluoxetine treatment decreases the expression of necroptotic kinases in CUMS mice.

5W FLX RIP1-
1+2

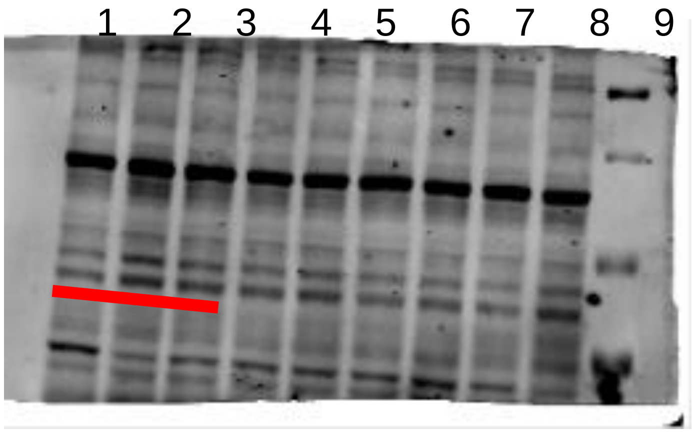

Cont=1+4
+7
CUMS=2+
5+8
CUMS+FL
X= 3+6+9

5W FLX RIP1 B.A

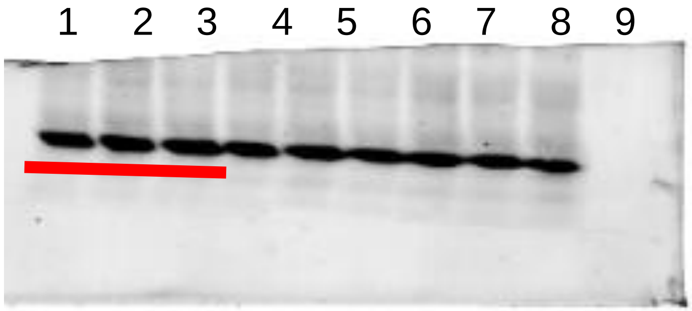

Fluoxetine treatment decreases the expression of necroptotic kinases in CUMS mice.

HP 5W P.RIP1-1

1 2 3 4 5 6 7 8 9 10

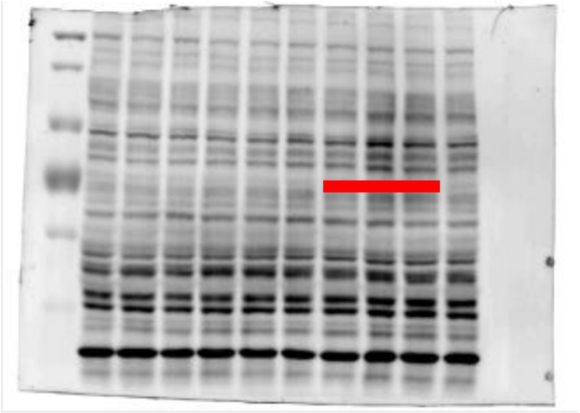

Cont=1+4 +7
CUMS=2+ 5+8
CUMS+FL X= 3+6+9+10

HP 5W P.RIP1-1
B.A

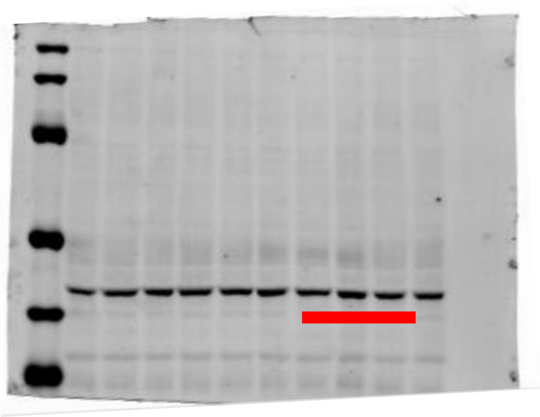

Cont=1+4 +7
CUMS=2+ 5+8
CUMS+FL X= 3+6+9+10

Fluoxetine treatment decreases the expression of necroptotic kinases in CUMS mice.

HP 5W FLX RIP3-1

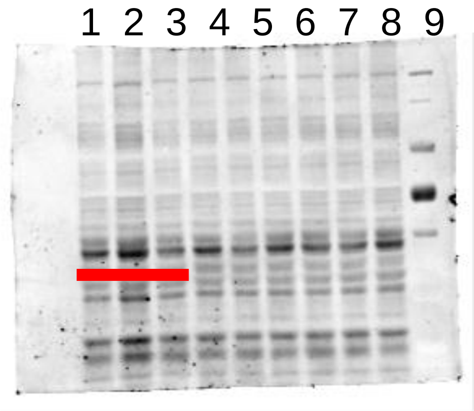

Cont=1+4+7
CUMS=2+5+8
CUMS+FLX = 3+6+9

HP 5W FLX RIP3 B.A

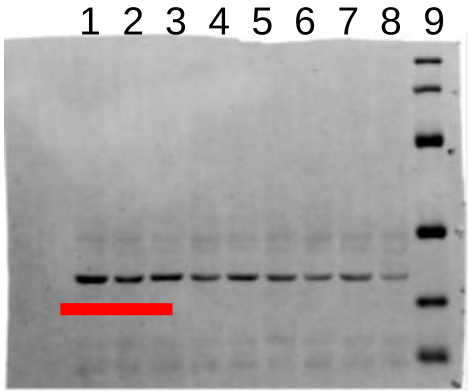

Fluoxetine treatment decreases the expression of necroptotic kinases in CUMS mice.

HP 5W
P.RIP3-1

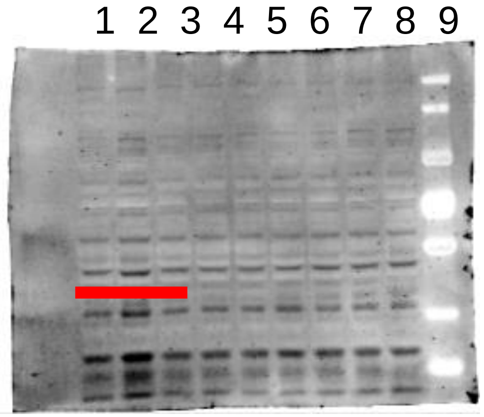

Cont=1+4+
7
CUMS=2+5
+8
CUMS+FLX
= 3+6+9

HP 5W P.RIP3-1
B.A

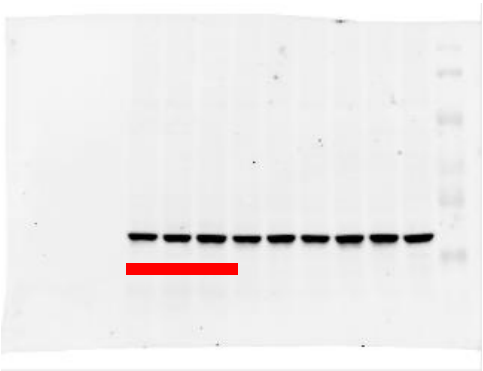

Cont=1+4+
7
CUMS=2+5
+8
CUMS+FLX
= 3+6+9

Fluoxetine treatment decreases the expression of necroptotic kinases in CUMS mice.

HP 5W MLKL-1

1 2 3 4 5 6

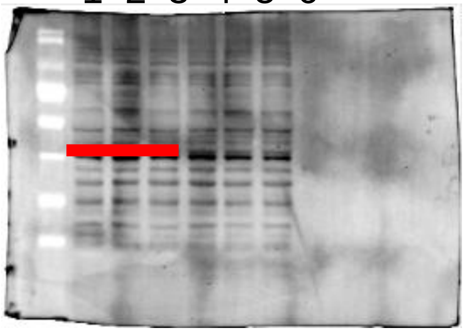

Cont=1+4
CUMS=2+5
CUMS+FLX
= 3+6

HP 5W MLKL-1
B.A

1 2 3 4 5 6

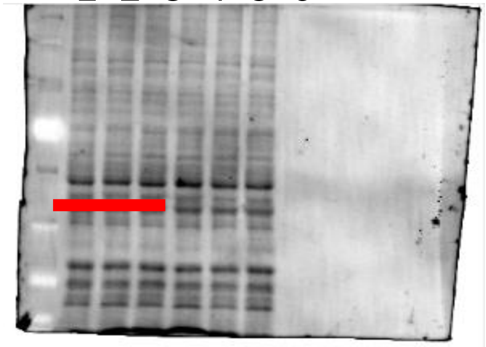

Cont=1+4
CUMS=2+5
CUMS+FLX
= 3+6

Fluoxetine treatment decreases the expression of necroptotic kinases in CUMS mice.

HP 5W
PMLKL-1

1 2 3 4 5 6

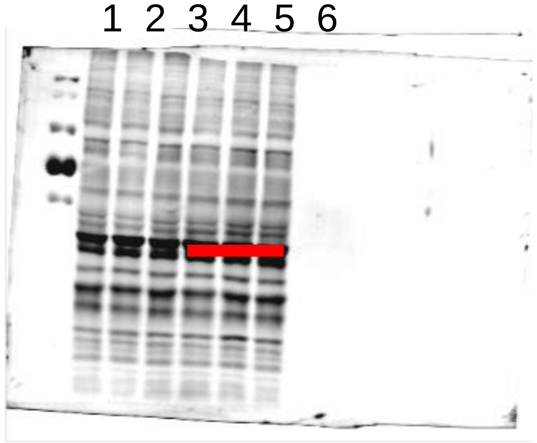

Cont=1+4
CUMS=2+5
CUMS+FLX
= 3+6

HP 5W PMLKL-1
B.A

1 2 3 4 5 6

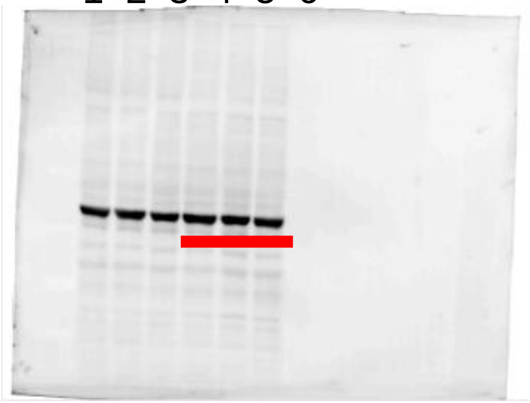

Fluoxetine treatment decreases the expression of RIP1K in the hippocampus of mice

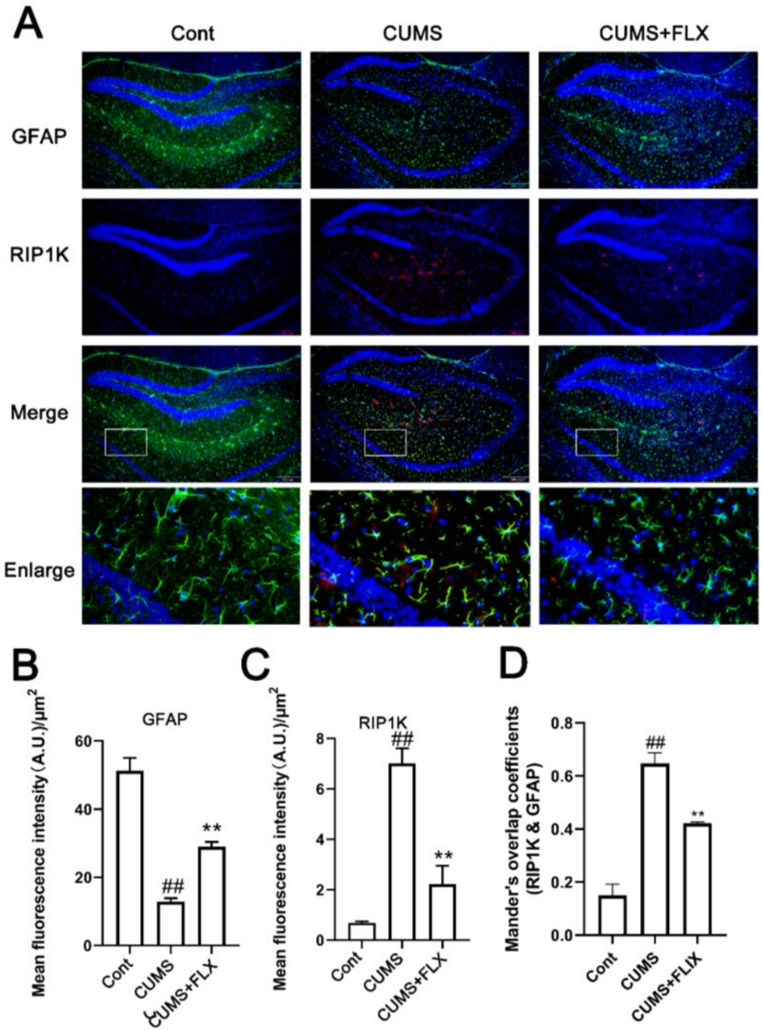

Fluoxetine treatment decreases the expression of RIP1K in the hippocampus of mice

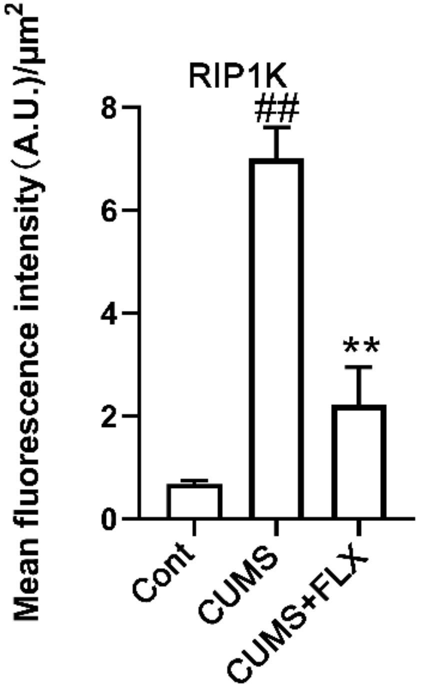

RIP1K	Cont	CUMS	CUMS+FLX
Mean fluoresence	0.745	6.356	3.059
intensity (A.U.)/μm2	0.605	7.197	1.775
	0.671	7.498	1.836

Tukey's multiple comparisons test	Mean Diff.	95.00% CI of diff.	Below threshold?	Summary	Adjusted P Value	q	DF
Column A vs. Column B	-6.343	-7.700 to -4.987	Yes	****	<0.0001	20.29	6
Column A vs. Column C	-1.55	-2.906 to -0.1929	Yes	*	0.0295	4.956	6
Column B vs. Column C	4.794	3.437 to 6.150	Yes	****	<0.0001	15.33	6

Fluoxetine treatment decreases the expression of RIP1K in the hippocampus of mice

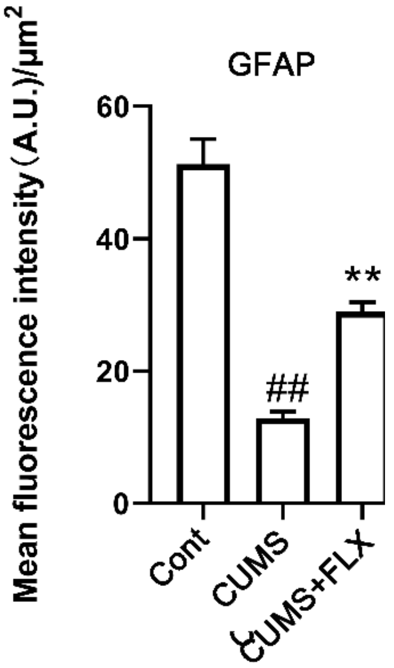

GFAP	Cont	CUMS	CUMS+FLX
Mean fluoresence	47.31	12.348	29.043
intensity (A.U.)/μm2	54.755	12.351	30.338
	51.791	14.029	27.458

Tukey's multiple comparisons test	Mean Diff.	95.00% CI of diff.	Below threshold?	Summary	Adjusted P Value	q	DF
Column A vs. Column B	-0.108	-0.1770 to -0.03900	Yes	**	0.0072	6.791	6
Column A vs. Column C	0.051	-0.01800 to 0.1200	No	ns	0.1373	3.207	6
Column B vs. Column C	0.159	0.09000 to 0.2280	Yes	***	0.001	9.998	6

Fluoxetine treatment decreases the expression of RIP1K in the hippocampus of mice

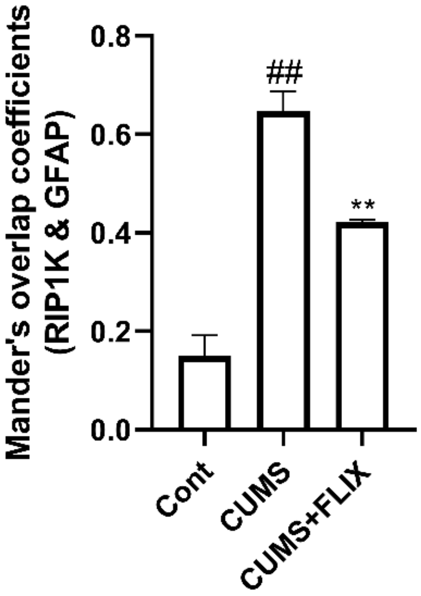

Mander's overlap coefficients	Cont	CUMS	CUMS+FLX
(RIP1 & GFAP)	0.125	0.665	0.42
	0.198	0.601	0.427
	0.126	0.675	0.418

Tukey's multiple comparisons test	Mean Diff.	95.00% CI of diff.	Below threshold?	Summary	Adjusted P Value	n2	q
Cont vs. CUMS	-0.1517	-0.1744 to -0.1289	Yes	****	<0.0001	3	28.93
Cont vs. CUMS+FLX	-0.08767	-0.1104 to -0.06492	Yes	****	<0.0001	3	16.72
CUMS vs. CUMS+FLX	0.064	0.04125 to 0.08675	Yes	***	0.0003	3	12.21

Fluoxetine treatment decreases the expression of RIP3K in the hippocampus of mice

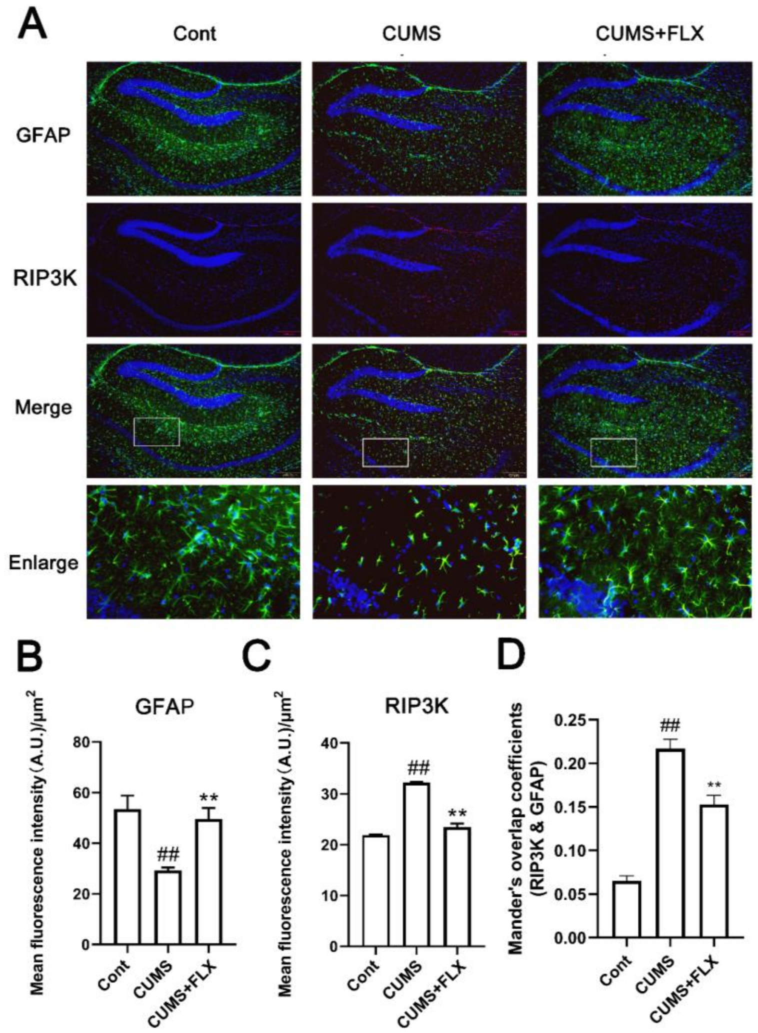

Fluoxetine treatment decreases the expression of RIP3K in the hippocampus of mice

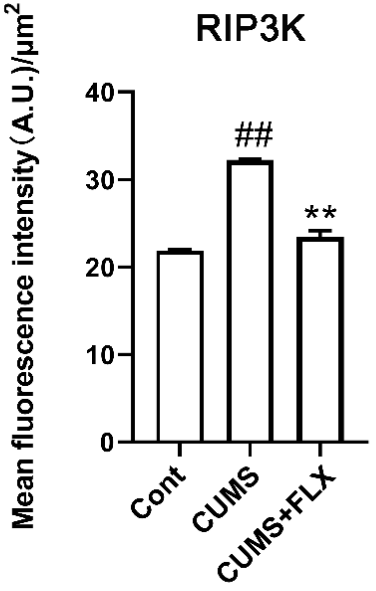

RIP3K	Cont	CUMS	CUMS+FLX
Mean fluoresence	21.9	32.096	23.59
intensity (A.U.)/μm2	21.577	32.25	22.688
	21.976	32.397	24.077

Tukey's multiple comparisons test	Mean Diff.	95.00% CI of diff.	Below threshold?	Summary	Adjusted P Value	n2	q
Column A vs. Column B	-10.43	-11.52 to -9.344	Yes	****	<0.0001	3	41.66
Column A vs. Column C	-1.634	-2.720 to -0.5475	Yes	**	0.0087	3	6.526
Column B vs. Column C	8.796	7.710 to 9.882	Yes	****	<0.0001	3	35.13

Fluoxetine treatment decreases the expression of RIP3K in the hippocampus of mice

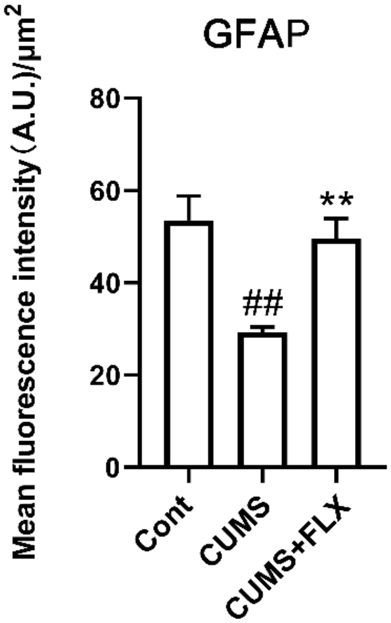

GFAP	Cont	CUMS	CUMS+FLX
Mean fluoresence	58.047	28.054	51.055
intensity (A.U.)/μm2	47.804	30.446	44.746
	54.78	29.186	52.983

Tukey's multiple comparisons test	Mean Diff.	95.00% CI of diff.	Below threshold?	Summary	Adjusted P Value	n2	q
Cont vs. CUMS	-0.318	-0.3509 to -0.2851	Yes	****	<0.0001	3	41.96
Cont vs. CUMS+FLX	-0.189	-0.2219 to -0.1561	Yes	****	<0.0001	3	24.94
CUMS vs. CUMS+FLX	0.129	0.09611 to 0.1619	Yes	****	<0.0001	3	17.02

Fluoxetine treatment decreases the expression of RIP3K in the hippocampus of mice

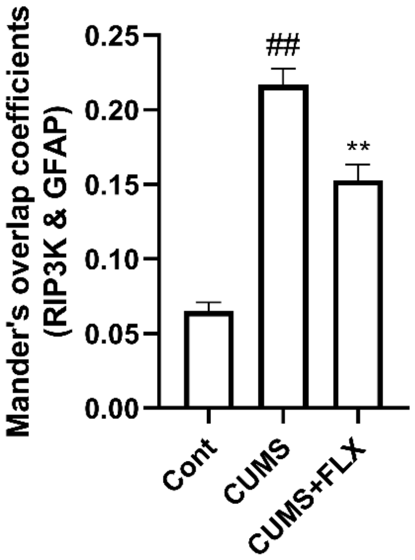

Mander's overlap coefficients (RIP3 & GFAP)	Cont	CUMS	CUMS+FLX
	0.07	0.205	0.164
	0.059	0.221	0.151
	0.067	0.225	0.144

Tukey's multiple comparisons test	Mean Diff.	95.00% CI of diff.	Below threshold?	Summary	Adjusted P Value	q	DF
Cont vs. CUMS	-0.1517	-0.1744 to -0.1289	Yes	****	<0.0001	28.93	6
Cont vs. CUMS+FLX	-0.08767	-0.1104 to -0.06492	Yes	****	<0.0001	16.72	6
CUMS vs. CUMS+FLX	0.064	0.04125 to 0.08675	Yes	***	0.0003	12.21	6

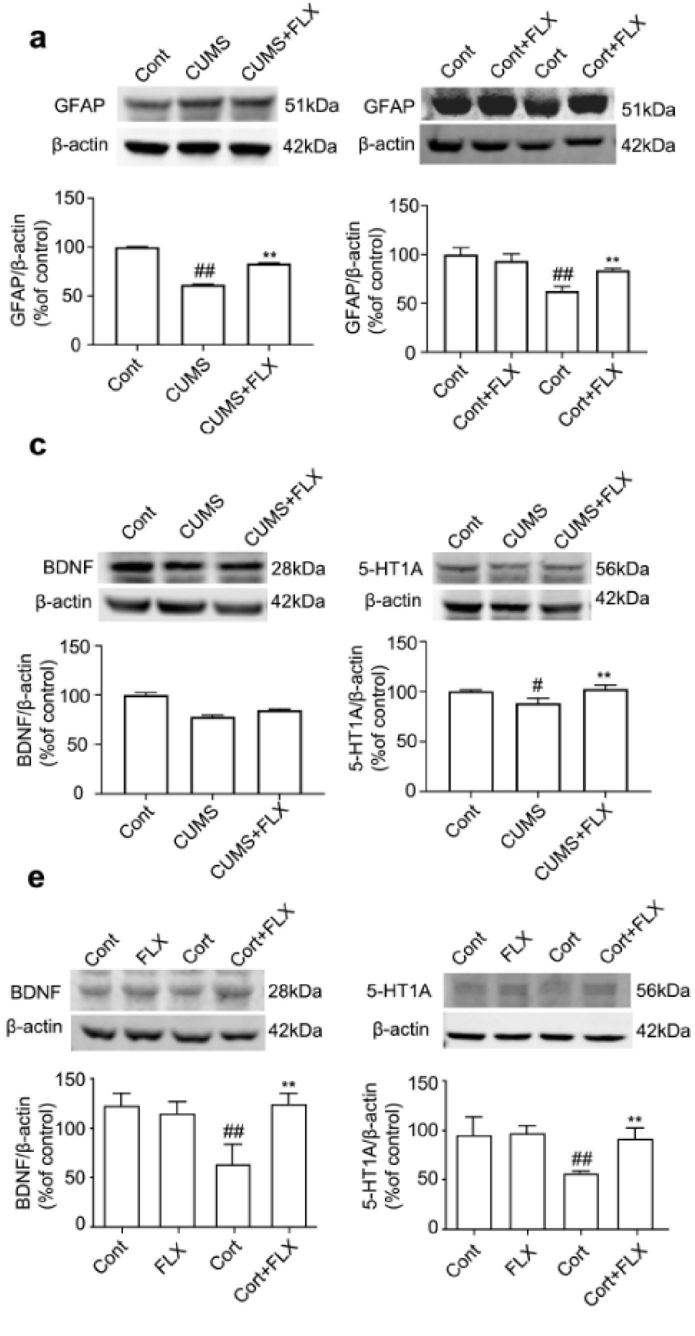

a
c
e
GFAP
β-actin
51kDa
42kDa
BDNF
28kDa
5-HT1A
56kDa
GFAP/β-actin (%of control)
BDNF/β-actin (%of control)
5-HT1A/β-actin (%of control)
Cont
CUMS
CUMS+FLX
Cont+FLX
Cort
Cort+FLX
FLX
##
#
**

Fluoxetine inhibits CUMS-induced reduction in the level of GFAP in mice hippocampus.

HP GFAP-2

1 2 3 4 5 6 7 8 9

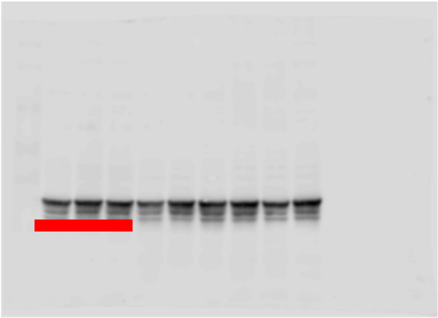

Cont=1+4+7
CUMS=2+5+8
CUMS+FLX = 3+6+9

HP GFAP-2 B.A

1 2 3 4 5 6 7 8 9

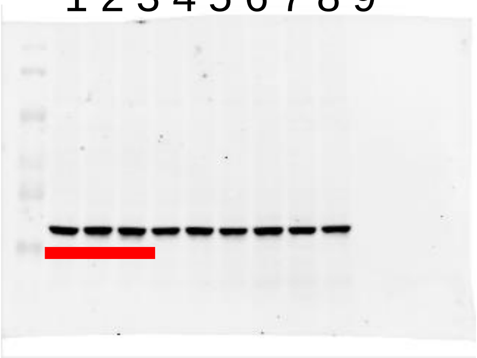

Fluoxetine inhibits the Cort-induced reduction in the GFAP protein level in human astrocytes.

FLX GFAP-1

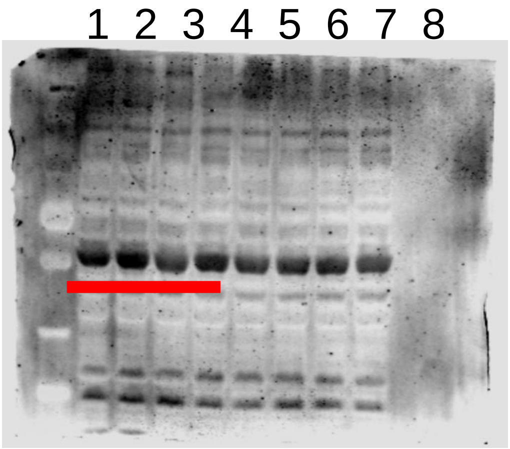

1+5= Cont
2+6= C+FLX
3+7= Cort 200
4+8= Cort+FLX

FLX GFAP-1 B.A

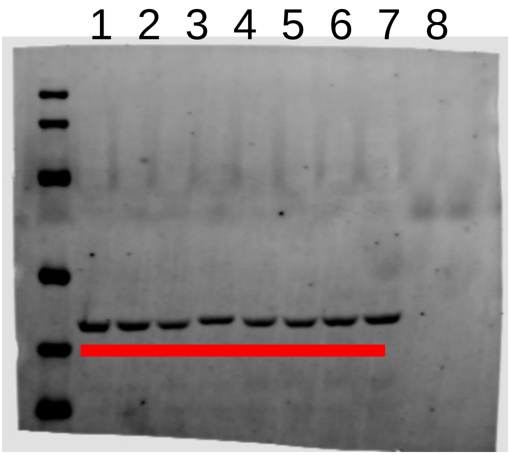

Fluoxetine prevents stress-induced reduction in the BDNF and 5-HT1A protein level in vivo and in vitro

HP BDNF
1+2

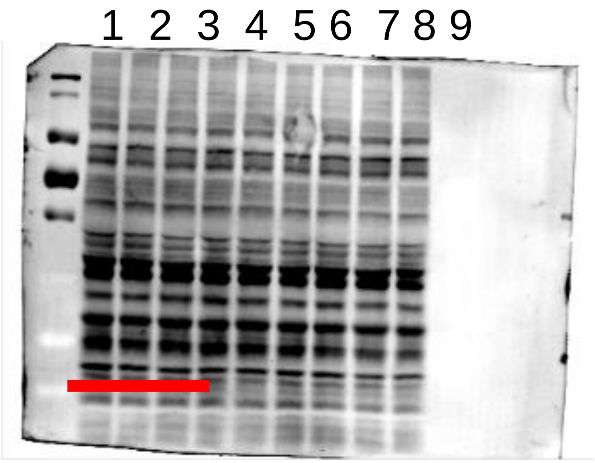

Cont=1+4+7
CUMS=2+5+8
CUMS+FLX = 3+6+9

HP BDNF-1+2
B.A

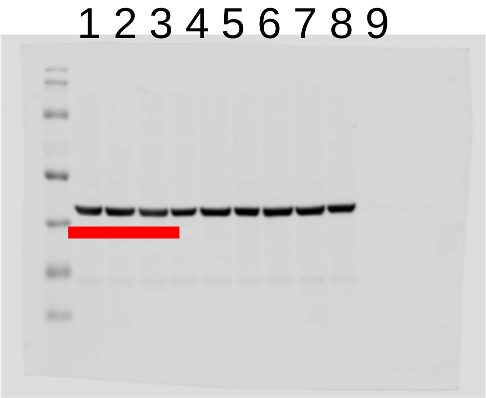

Fluoxetine prevents stress-induced reduction 5-HT1A protein level in vivo and in vitro

HP 5HT1A-1+2

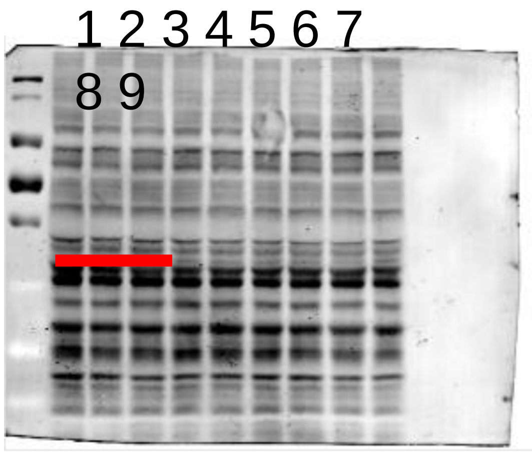

Cont=1+4+7
CUMS=2+5+8
CUMS+FLX = 3+6+9

HP 5HT1A-1+2 B.A

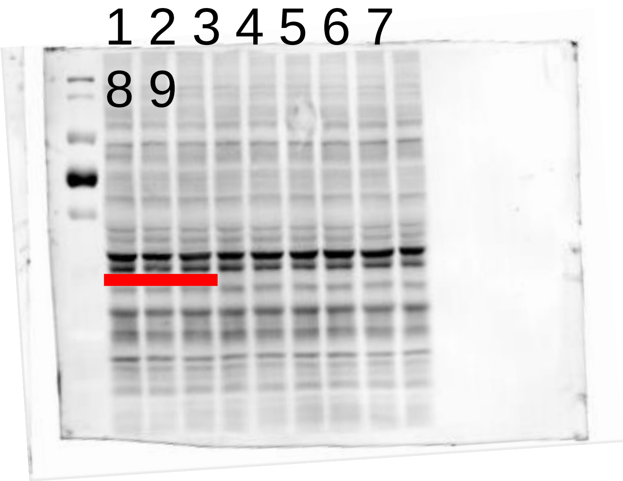

Fluoxetine prevents Cort-induced reduction BDNF protein level in vitro

FLX BDNF-2

FLX BDNF-2 B.A

1+5= Cont
2+6= C+FLX
3+7= Cort 200
4+8= Cort+FLX

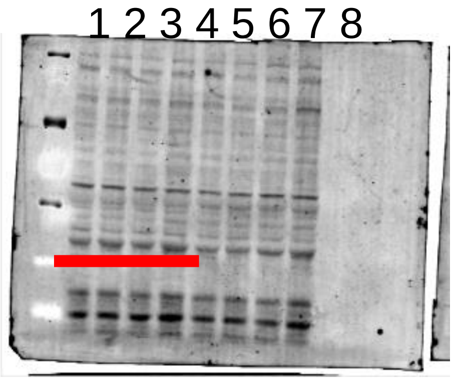

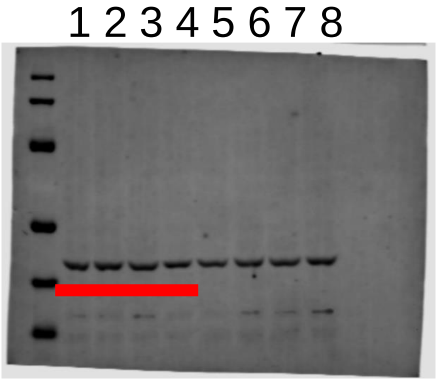

Fluoxetine prevents Cort-induced reduction 5-HT1A protein level in vitro

1= Cont
2= C+FLX
3= Cort
200
4=
Cort+FLX

FLX 5HT1A-2

1 2 3 4

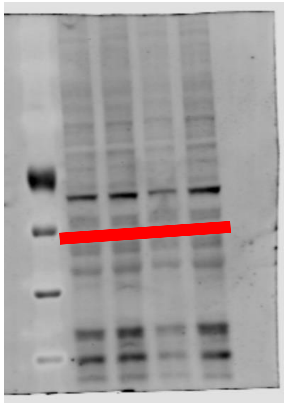

FLX 5HT1A
B.A-2

1 2 3 4

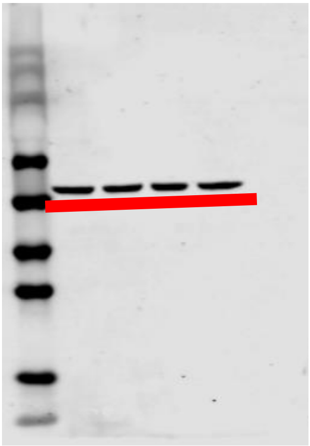

Fluoxetine protects the human astrocytes against Cort-induced injury

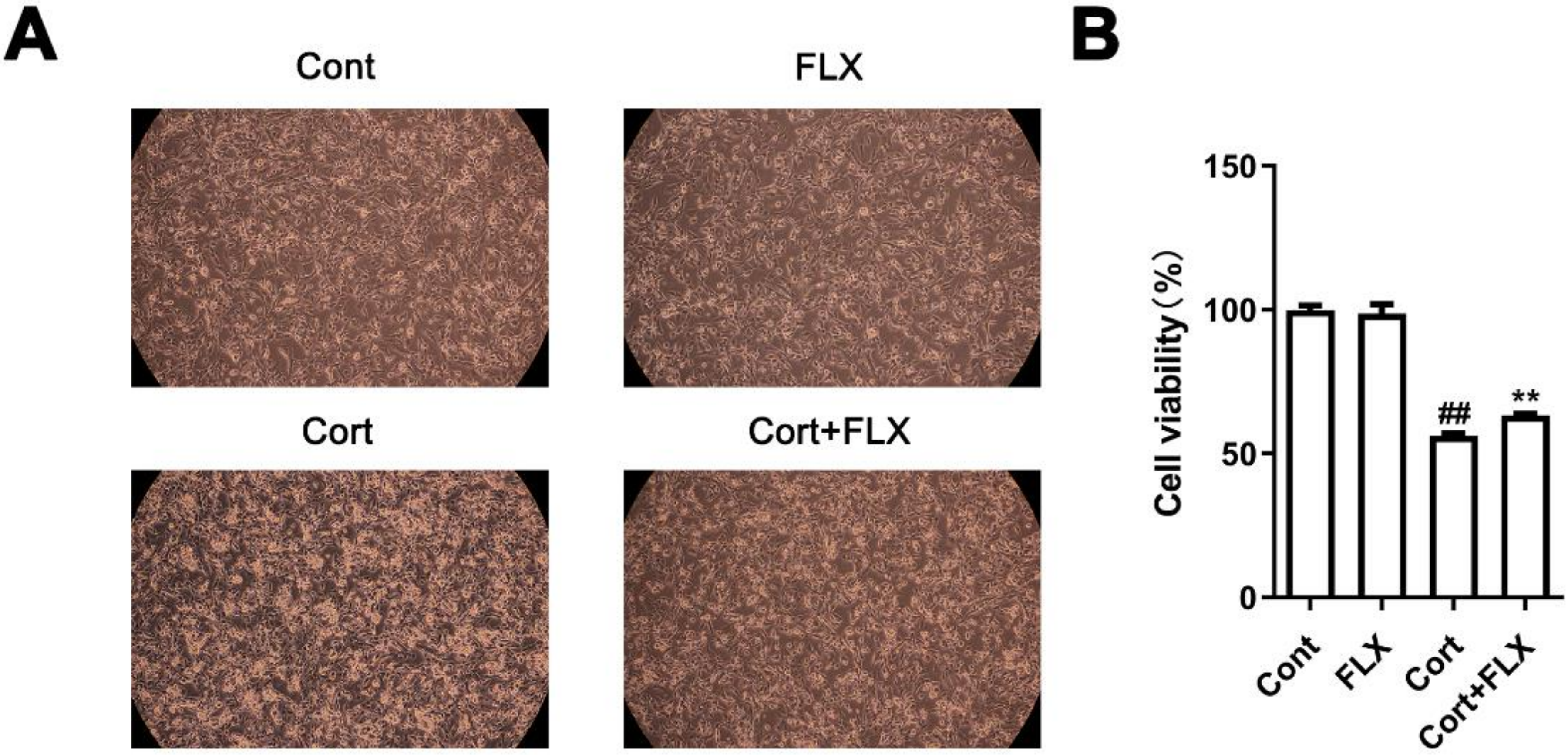

Fluoxetine protects the human astrocytes against Cort-induced injury

CCK8 Test					
	Blank	Cont	DMSO	Cort 200	Cort+FLX
	0.165800005	0.681399989	0.666999884	0.445669998	0.490399999
	0.157600001	0.667599976	0.686790008	0.45367001	0.485649989
	0.159799993	0.678900003	0.653900011	0.451699993	0.483229908
	0.160999998	0.727400005	0.683000006	0.443799997	0.501299994
	0.154400006	0.690199971	0.684300015	0.431199995	0.499549989
**Average**	0.159720001	0.689099989	0.674997985	0.445207999	0.492025976
**SD**	0.004225163	0.022878382	0.014130694	0.008834191	0.008113045
**Each gp av OD-BL av OD**		0.529379988	0.515277984	0.285487998	0.332305975
**Cell Viability**		100	97.33612818	55.40465668	64.49062164

Fluoxetine protects the human astrocytes against Cort-induced injury

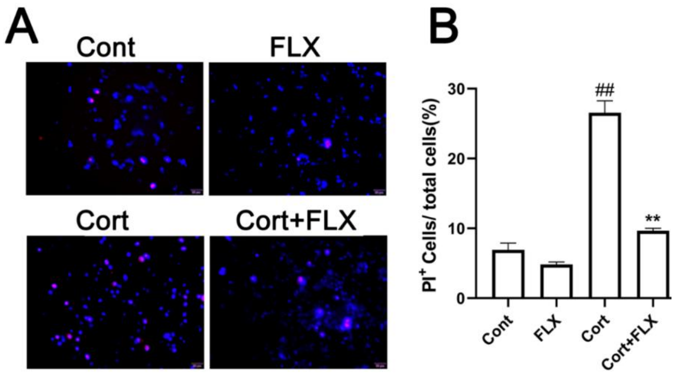

FLX effect on Cort HA Cells	Cont	Cont+FLX	Cort	Cort+FLX
PI+ Cells/ total cells(%)	6.862745	4.424779	28.08989	9.259259
	7.920792	5.042017	26.88172	9.803922
	5.940594	5.050505	24.73118	9.90991

Tukey's multiple comparisons test	Mean Diff.	95.00% CI of diff.	Below threshold?	Summary	Adjusted P Value	q	DF
Cont vs. Cont+FLX	2.069	-0.5869 to 4.725	No	ns	0.1354	3.528	8
Cont vs. Cort	-19.66	-22.32 to -17.00	Yes	****	<0.0001	33.52	8
Cont vs. Cort+FLX	-2.75	-5.405 to -0.09385	Yes	*	0.0427	4.689	8
Cont+FLX vs. Cort	-21.73	-24.38 to -19.07	Yes	****	<0.0001	37.05	8
Cont+FLX vs. Cort+FLX	-4.819	-7.474 to -2.163	Yes	**	0.0018	8.217	8
Cort vs. Cort+FLX	16.91	14.25 to 19.57	Yes	****	<0.0001	28.84	8

The combined administration of low dose fluoxetine and necroptotic kinase inhibitors further increases cell viability in Cort-treated HA cells.

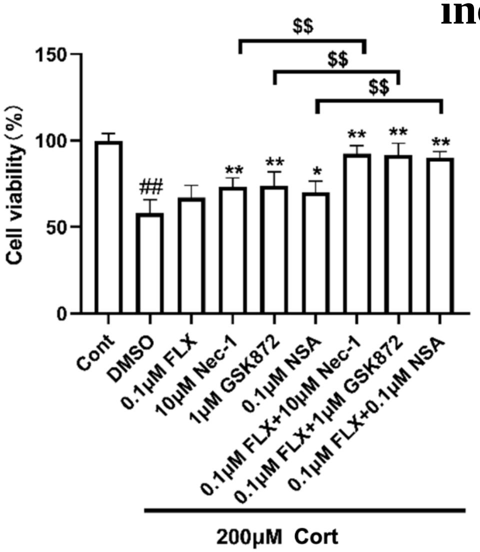

Cell viability									
	Cont	DMSO	0.1μM FLX	10μM Nec-1	1μM GSK872	0.1μM NSA	0.1μM FLX+10μM Nec-1	0.1μM FLX+1μM GSK872	0.1μM FLX+0.1μM NSA
	96.51548	52.01563	74.40073	71.08441	80.25833	70.83209	96.10093	82.9258	83.59267
	97.14629	65.37098	57.85522	66.21808	74.16641	61.94653	96.40733	97.12826	92.02764
	99.97597	49.5284	65.37098	76.924	66.65064	75.1577	88.26074	96.6236	94.74917
	96.35325	53.11504	65.20876	78.47401	82.52927	74.94142	96.01081	99.14688	88.47701
	104.9865	68.79543	63.80294	77.28447	77.62691	61.65815	92.06368	87.17933	90.58576
	105.0225	58.97267	76.09491	70.86812	62.01862	75.33793	85.70141	87.12526	89.95493

The combined administration of low dose fluoxetine and necroptotic kinase inhibitors further increases cell viability in Cort-treated HA cells.

Tukey's multiple comparisons test	Mean Diff.	95.00% CI of diff.	Below threshold?	Summary	Adjusted P Value	q	DF
Cont vs. DMSO	42.03	30.54 to 53.53	Yes	****	<0.0001	16.85	45
Cont vs. 0.1μM FLX	32.88	21.39 to 44.37	Yes	****	<0.0001	13.18	45
Cont vs. 10μM Nec-1	26.52	15.03 to 38.02	Yes	****	<0.0001	10.63	45
Cont vs. 1μM GSK872	26.12	14.63 to 37.62	Yes	****	<0.0001	10.47	45
Cont vs. 0.1μM NSA	30.02	18.53 to 41.51	Yes	****	<0.0001	12.03	45
Cont vs. 0.1μM FLX+10μM Nec-1	7.576	-3.916 to 19.07	No	ns	0.456	3.037	45
Cont vs. 0.1μM FLX+1μM GSK872	8.312	-3.180 to 19.80	No	ns	0.3325	3.332	45
Cont vs. 0.1μM FLX+0.1μM NSA	10.1	-1.390 to 21.59	No	ns	0.1257	4.049	45
DMSO vs. 0.1μM FLX	-9.156	-20.65 to 2.336	No	ns	0.2174	3.67	45
DMSO vs. 10μM Nec-1	-15.51	-27.00 to -4.017	Yes	**	0.002	6.216	45
DMSO vs. 1μM GSK872	-15.91	-27.40 to -4.417	Yes	**	0.0014	6.377	45
DMSO vs. 0.1μM NSA	-12.01	-23.50 to -0.5206	Yes	*	0.0343	4.815	45
DMSO vs. 0.1μM FLX+10μM Nec-1	-34.46	-45.95 to -22.97	Yes	****	<0.0001	13.81	45
DMSO vs. 0.1μM FLX+1μM GSK872	-33.72	-45.21 to -22.23	Yes	****	<0.0001	13.52	45
DMSO vs. 0.1μM FLX+0.1μM NSA	-31.93	-43.42 to -20.44	Yes	****	<0.0001	12.8	45
0.1μM FLX vs. 10μM Nec-1	-6.353	-17.85 to 5.139	No	ns	0.6814	2.547	45
0.1μM FLX vs. 1μM GSK872	-6.753	-18.24 to 4.739	No	ns	0.6081	2.707	45

The combined administration of low dose fluoxetine and necroptotic kinase inhibitors further increases cell viability in Cort-treated HA cells.

0.1μM FLX vs. 0.1μM NSA	-2.857	-14.35 to 8.635	No	ns	0.996	1.145	45
0.1μM FLX vs. 0.1μM FLX+10μM Nec-1	-25.3	-36.79 to -13.81	Yes	****	<0.0001	10.14	45
0.1μM FLX vs. 0.1μM FLX+1μM GSK872	-24.57	-36.06 to -13.07	Yes	****	<0.0001	9.847	45
0.1μM FLX vs. 0.1μM FLX+0.1μM NSA	-22.78	-34.27 to -11.28	Yes	****	<0.0001	9.129	45
10μM Nec-1 vs. 1μM GSK872	-0.3995	-11.89 to 11.09	No	ns	>0.9999	0.1601	45
10μM Nec-1 vs. 0.1μM NSA	3.497	-7.996 to 14.99	No	ns	0.9849	1.401	45
10μM Nec-1 vs. 0.1μM FLX+10μM Nec-1	-18.95	-30.44 to -7.457	Yes	****	<0.0001	7.595	45
10μM Nec-1 vs. 0.1μM FLX+1μM GSK872	-18.21	-29.70 to -6.721	Yes	***	0.0002	7.3	45
10μM Nec-1 vs. 0.1μM FLX+0.1μM NSA	-16.42	-27.91 to -4.930	Yes	***	0.0009	6.582	45
1μM GSK872 vs. 0.1μM NSA	3.896	-7.596 to 15.39	No	ns	0.9706	1.562	45
1μM GSK872 vs. 0.1μM FLX+10μM Nec-1	-18.55	-30.04 to -7.057	Yes	***	0.0001	7.435	45
1μM GSK872 vs. 0.1μM FLX+1μM GSK872	-17.81	-29.31 to -6.321	Yes	***	0.0003	7.14	45
1μM GSK872 vs. 0.1μM FLX+0.1μM NSA	-16.02	-27.51 to -4.531	Yes	**	0.0013	6.422	45
0.1μM NSA vs. 0.1μM FLX+10μM Nec-1	-22.45	-33.94 to -10.95	Yes	****	<0.0001	8.997	45
0.1μM NSA vs. 0.1μM FLX+1μM GSK872	-21.71	-33.20 to -10.22	Yes	****	<0.0001	8.702	45
0.1μM NSA vs. 0.1μM FLX+0.1μM NSA	-19.92	-31.41 to -8.427	Yes	****	<0.0001	7.984	45
0.1μM FLX+10μM Nec-1 vs. 0.1μM FLX+1μM GSK872	0.736	-10.76 to 12.23	No	ns	>0.9999	0.295	45
0.1μM FLX+10μM Nec-1 vs. 0.1μM FLX+0.1μM NSA	2.526	-8.966 to 14.02	No	ns	0.9983	1.013	45
0.1μM FLX+1μM GSK872 vs. 0.1μM FLX+0.1μM NSA	1.79	-9.702 to 13.28	No	ns	0.9999	0.7176	45

CUMS increases the levels of inflammatory cytokines in mice hippocampus in a time-dependent manner

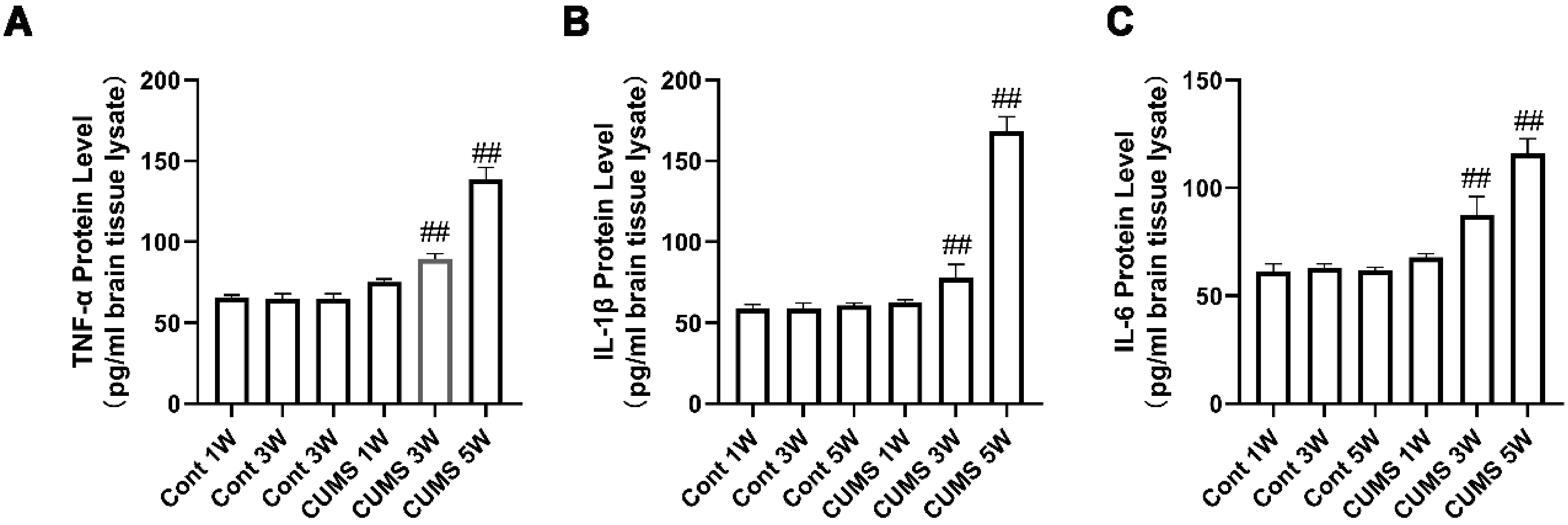

CUMS increases the levels of inflammatory cytokines in mice hippocampus in a time-dependent manner

A

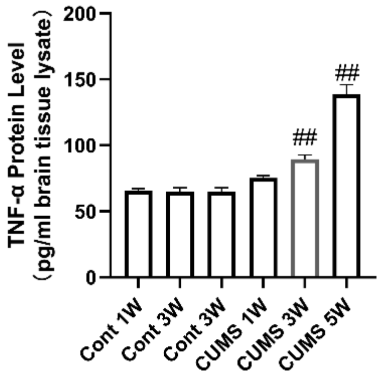

OD VALUE						
Groups	**Cont 1 W**	**CUMS 1 W**	**Cont 3 W**	**CUMS 3 W**	**Cont 5 W**	**CUMS 5 W**
**TNF-α**	0.2077	0.2518	0.2198	0.2845	0.2131	0.4513
**1 + 3 + 5 Week**	0.2171	0.2447	0.2148	0.2881	0.2258	0.4287
	0.217	0.2396	0.202	0.3039	0.2211	0.4758
**Protein Level**	63.71165644	77.2392638	67.42331288	87.26993865	65.36809816	138.4355828
	66.59509202	75.06134969	65.88957055	88.37423313	69.26380368	131.5030675
	66.56441718	73.49693252	61.96319018	93.2208589	67.82208589	145.9509202

Tukey's multiple comparisons test	Mean Diff.	95.00% CI of diff.	Below threshold?	Summary	Adjusted P Value	q	DF
Cont 1W vs. Cont 3W	0.5317	-9.503 to 10.57	No	ns	>0.9999	0.2517	12
Cont 1W vs. Cont 5W	-1.861	-11.90 to 8.173	No	ns	0.987	0.881	12
Cont 1W vs. CUMS 1W	-9.642	-19.68 to 0.3921	No	ns	0.0622	4.565	12
Cont 1W vs. CUMS 3W	-24	-34.03 to -13.96	Yes	****	<0.0001	11.36	12
Cont 1W vs. CUMS 5W	-73.01	-83.04 to -62.97	Yes	****	<0.0001	34.56	12
Cont 3W vs. Cont 5W	-2.393	-12.43 to 7.642	No	ns	0.9619	1.133	12
Cont 3W vs. CUMS 1W	-10.17	-20.21 to -0.1396	Yes	*	0.0462	4.816	12
Cont 3W vs. CUMS 3W	-24.53	-34.56 to -14.50	Yes	****	<0.0001	11.61	12
Cont 3W vs. CUMS 5W	-73.54	-83.57 to -63.50	Yes	****	<0.0001	34.81	12
Cont 5W vs. CUMS 1W	-7.781	-17.82 to 2.253	No	ns	0.1694	3.684	12
Cont 5W vs. CUMS 3W	-22.14	-32.17 to -12.10	Yes	****	<0.0001	10.48	12
Cont 5W vs. CUMS 5W	-71.15	-81.18 to -61.11	Yes	****	<0.0001	33.68	12
CUMS 1W vs. CUMS 3W	-14.36	-24.39 to -4.322	Yes	**	0.0044	6.796	12
CUMS 1W vs. CUMS 5W	-63.36	-73.40 to -53.33	Yes	****	<0.0001	30	12
CUMS 3W vs. CUMS 5W	-49.01	-59.04 to -38.97	Yes	****	<0.0001	23.2	12

CUMS increases the levels of inflammatory cytokines in mice hippocampus in a time-dependent manner

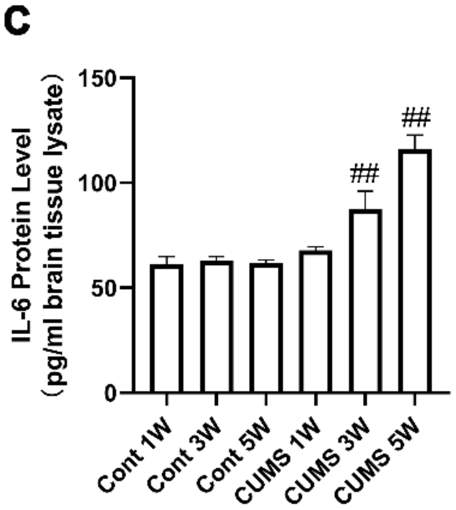

OD VALUE						
**Groups**	**Cont 1 W**	**CUMS 1 W**	**Cont 3 W**	**CUMS 3 W**	**Cont 5 W**	**CUMS 5 W**
**IL-6**	0.2263	0.2452	0.2275	0.3148	0.2249	0.4511
**1 + 3 + 5 Week**	0.2386	0.2569	0.2398	0.3567	0.2338	0.4021
	0.2125	0.2474	0.2263	0.2936	0.2245	0.4298
**Protein Level**	61.46116241	66.59424226	61.78707224	85.49701249	61.08093427	122.5149375
	64.80173819	69.77186312	65.12764802	96.87669745	63.49809886	109.2069527
	57.71319935	67.19174362	61.46116241	79.73927213	60.97229766	116.730038

Tukey's multiple comparisons test	Mean Diff.	95.00% CI of diff.	Below threshold?	Summary	Adjusted P Value	q	DF
Cont 1W vs. Cont 3W	-1.467	-14.82 to 11.88	No	ns	0.9989	0.5218	12
Cont 1W vs. Cont 5W	-0.5251	-13.88 to 12.82	No	ns	>0.9999	0.1868	12
Cont 1W vs. CUMS 1W	-6.527	-19.88 to 6.823	No	ns	0.5891	2.323	12
Cont 1W vs. CUMS 3W	-26.05	-39.40 to -12.70	Yes	***	0.0003	9.268	12
Cont 1W vs. CUMS 5W	-54.83	-68.18 to -41.48	Yes	****	<0.0001	19.51	12
Cont 3W vs. Cont 5W	0.9415	-12.41 to 14.29	No	ns	0.9999	0.335	12
Cont 3W vs. CUMS 1W	-5.061	-18.41 to 8.289	No	ns	0.7934	1.801	12
Cont 3W vs. CUMS 3W	-24.58	-37.93 to -11.23	Yes	***	0.0005	8.746	12
Cont 3W vs. CUMS 5W	-53.36	-66.71 to -40.01	Yes	****	<0.0001	18.99	12
Cont 5W vs. CUMS 1W	-6.002	-19.35 to 7.348	No	ns	0.665	2.136	12
Cont 5W vs. CUMS 3W	-25.52	-38.87 to -12.17	Yes	***	0.0004	9.081	12
Cont 5W vs. CUMS 5W	-54.3	-67.65 to -40.95	Yes	****	<0.0001	19.32	12
CUMS 1W vs. CUMS 3W	-19.52	-32.87 to -6.168	Yes	**	0.0037	6.945	12
CUMS 1W vs. CUMS 5W	-48.3	-61.65 to -34.95	Yes	****	<0.0001	17.19	12
CUMS 3W vs. CUMS 5W	-28.78	-42.13 to -15.43	Yes	***	0.0001	10.24	12

CUMS increases the levels of inflammatory cytokines in mice hippocampus in a time-dependent manner

B

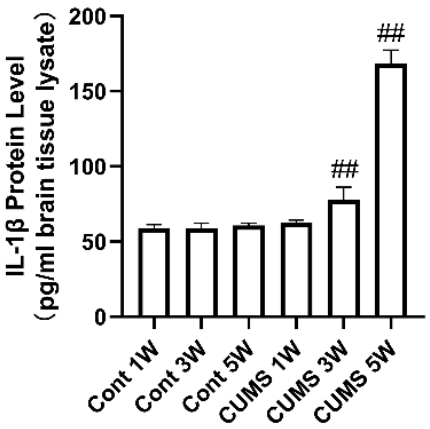

OD VALUE						
Groups	Cont 1 W	CUMS 1 W	Cont 3 W	CUMS 3 W	Cont 5 W	CUMS 5 W
IL-1β	0.1962	0.2157	0.1877	0.2883	0.1968	0.5324
1 + 3 + 5 Week	0.2054	0.2048	0.1927	0.2602	0.2062	0.5918
	0.1908	0.2059	0.2088	0.2324	0.2061	0.5657
Protein Level	58.6722488	64.50358852	56.13038278	86.21411483	58.85167464	159.2105263
	61.42344498	61.24401914	57.62559809	77.81100478	61.66267943	176.9736842
	57.05741627	61.57296651	62.44019139	69.49760766	61.63277512	169.1686603

Tukey's multiple comparisons test	Mean Diff.	95.00% CI of diff.	Below threshold?	Summary	Adjusted P Value	q	DF
Cont 1W vs. Cont 3W	0.319	-14.31 to 14.95	No	ns	>0.9999	0.1036	12
Cont 1W vs. Cont 5W	-1.665	-16.29 to 12.96	No	ns	0.9986	0.5406	12
Cont 1W vs. CUMS 1W	-3.389	-18.02 to 11.24	No	ns	0.9662	1.101	12
Cont 1W vs. CUMS 3W	-18.79	-33.42 to -4.161	Yes	**	0.01	6.102	12
Cont 1W vs. CUMS 5W	-109.4	-124.0 to -94.77	Yes	****	<0.0001	35.53	12
Cont 3W vs. Cont 5W	-1.984	-16.61 to 12.64	No	ns	0.9969	0.6441	12
Cont 3W vs. CUMS 1W	-3.708	-18.34 to 10.92	No	ns	0.9512	1.204	12
Cont 3W vs. CUMS 3W	-19.11	-33.74 to -4.480	Yes	**	0.0088	6.205	12
Cont 3W vs. CUMS 5W	-109.7	-124.3 to -95.09	Yes	****	<0.0001	35.63	12
Cont 5W vs. CUMS 1W	-1.724	-16.35 to 12.90	No	ns	0.9984	0.56	12
Cont 5W vs. CUMS 3W	-17.13	-31.75 to -2.497	Yes	*	0.019	5.561	12
Cont 5W vs. CUMS 5W	-107.7	-122.4 to -93.11	Yes	****	<0.0001	34.98	12
CUMS 1W vs. CUMS 3W	-15.4	-30.03 to -0.7723	Yes	*	0.0371	5.001	12
CUMS 1W vs. CUMS 5W	-106	-120.6 to -91.38	Yes	****	<0.0001	34.42	12
CUMS 3W vs. CUMS 5W	-90.61	-105.2 to -75.98	Yes	****	<0.0001	29.42	12

The necroptotic kinase inhibitors reduce Cort-induced increase in inflammatory cytokines in human astrocytes.

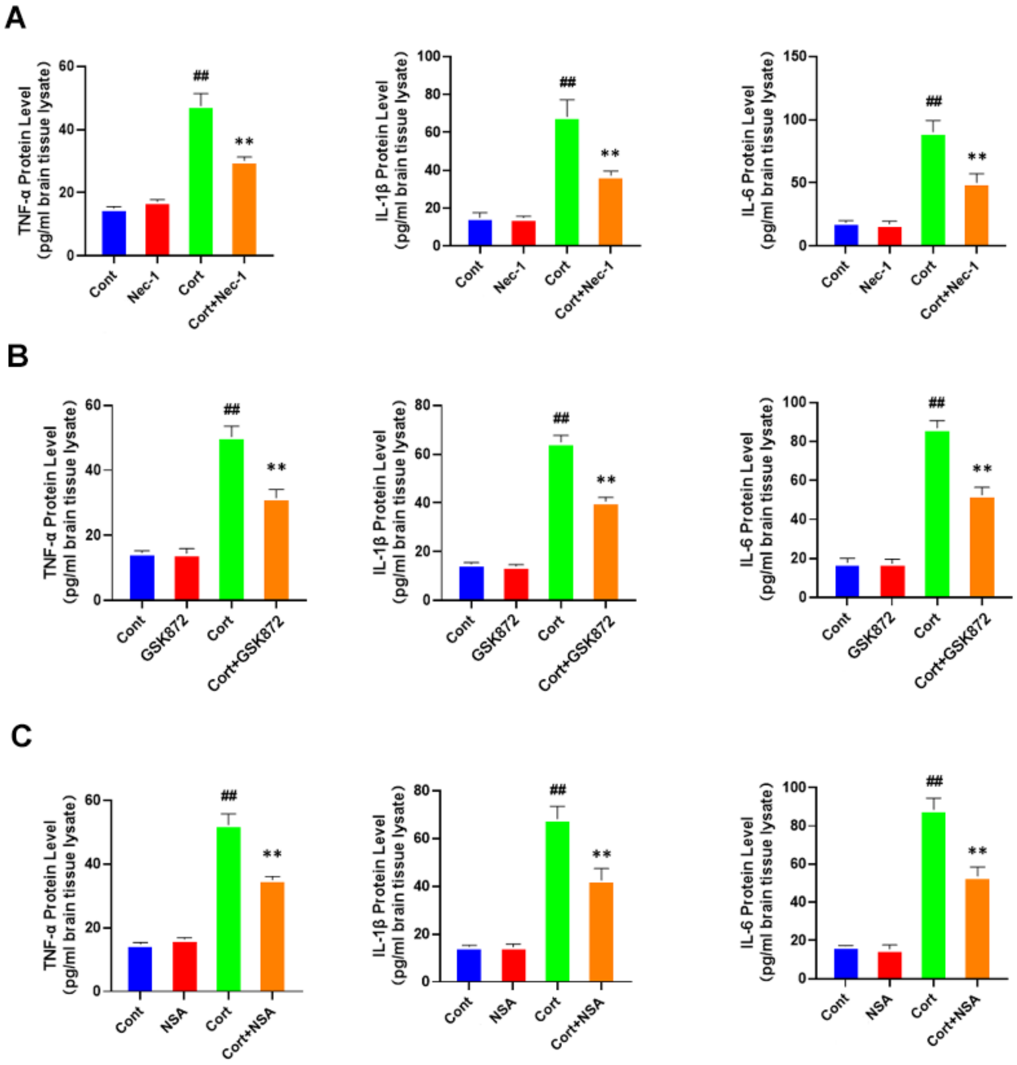

Nec-1 reduce Cort-induced increase in inflammatory cytokines in human astrocytes.

A

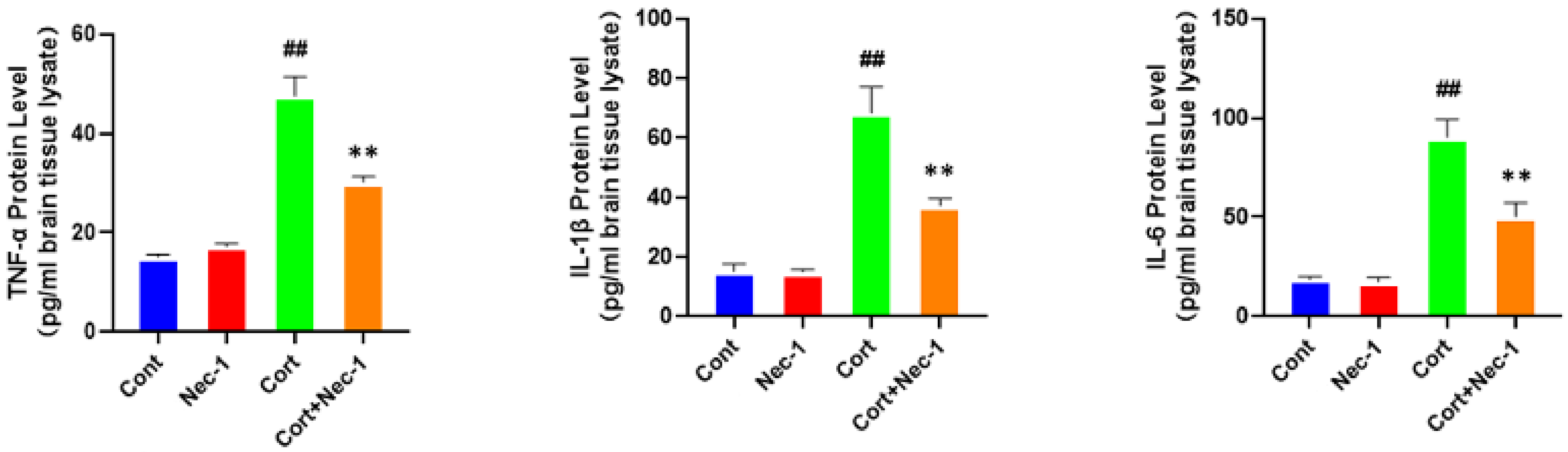

Nec-1 reduce Cort-induced increase in inflammatory cytokines in human astrocytes

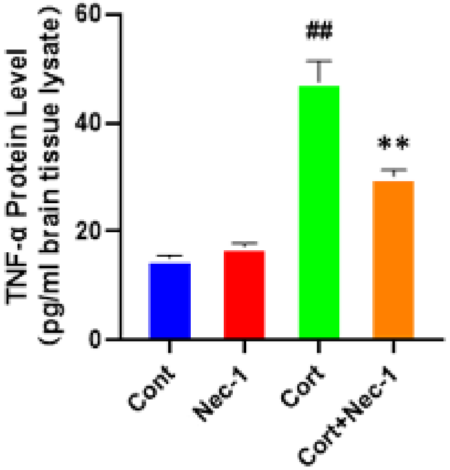

Nec-1				
TNF-α	Cont	C+Nec-1	Cort	Cort+Nec-1
OD VALUE	0.2076	0.2378	0.6842	0.4469
	0.2185	0.2546	0.7345	0.4358
	0.223	0.2491	0.6229	0.4145
Protein level	14.51748252	16.62937063	47.84615385	31.25174825
	15.27972028	17.8041958	51.36363636	30.47552448
	15.59440559	17.41958042	43.55944056	28.98601399

Tukey's multiple comparisons test	Mean Diff.	95.00% CI of diff.	Below threshold?	Summary	Adjusted P Value	q	DF
Cont vs. Cont+Nec-1	-2.154	-7.586 to 3.279	No	ns	0.6046	1.796	8
Cont vs. Cort	-32.46	-37.89 to -27.03	Yes	****	<0.0001	27.06	8
Cont vs. Cort+Nec-1	-15.11	-20.54 to -9.675	Yes	****	<0.0001	12.59	8
Cont+Nec-1 vs. Cort	-30.31	-35.74 to -24.87	Yes	****	<0.0001	25.26	8
Cont+Nec-1 vs. Cort+Nec-1	-12.95	-18.39 to -7.521	Yes	***	0.0003	10.8	8
Cort vs. Cort+Nec-1	17.35	11.92 to 22.78	Yes	****	<0.0001	14.47	8

Nec-1 reduce Cort-induced increase in inflammatory cytokines in human astrocytes

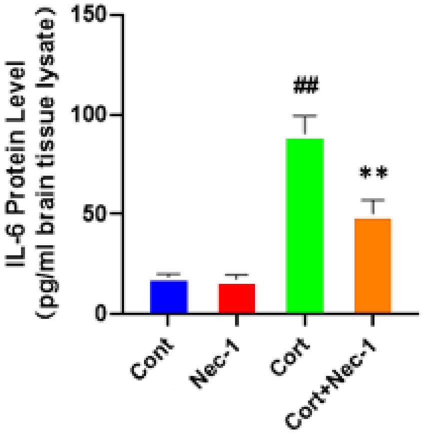

NEC-1				
**IL-6**	**Cont**	**Cont+Nec-1**	**Cort**	**Cort+Nec-1**
**OD VALUE**	0.2912	0.2819	1.26025	0.6121
	0.2697	0.262	1.4575	0.7954
	0.2528	0.2227	1.219	0.7822
**Protein level**	20.08275862	19.44137931	86.9137931	42.2137931
	18.6	18.06896552	100.5172414	54.85517241
	17.43448276	15.35862069	84.06896552	53.94482759

Tukey's multiple comparisons test	Mean Diff.	95.00% CI of diff.	Below threshold?	Summary	Adjusted P Value	q	DF
Cont vs. Cont+Nec-1	1.083	-14.00 to 16.16	No	ns	0.9954	0.3252	8
Cont vs. Cort	-71.79	-86.88 to -56.71	Yes	****	<0.0001	21.56	8
Cont vs. Cort+Nec-1	-31.63	-46.71 to -16.55	Yes	***	0.0007	9.499	8
Cont+Nec-1 vs. Cort	-72.88	-87.96 to -57.80	Yes	****	<0.0001	21.89	8
Cont+Nec-1 vs. Cort+Nec-1	-32.71	-47.80 to -17.63	Yes	***	0.0005	9.824	8
Cort vs. Cort+Nec-1	40.16	25.08 to 55.24	Yes	***	0.0001	12.06	8

Nec-1 reduce Cort-induced increase in inflammatory cytokines in human

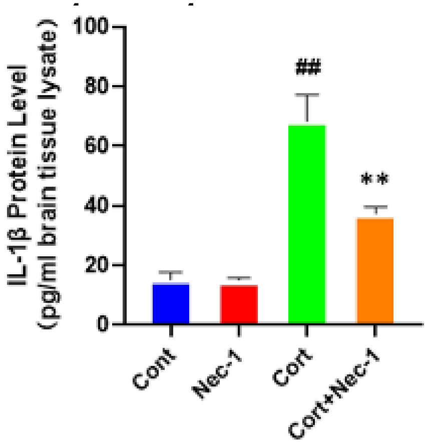

NEC-1				
**IL-1β**	**Cont**	**Cont+Nec-1**	**Cort**	**Cort+Nec-1**
**OD VALUE**	0.2501	0.212600002	0.851800004	0.482999998
	0.1962	0.204400002	0.774899997	0.517899993
	0.2014	0.22450001	0.986199993	0.463400006
**Protein level**	17.92682927	14.87804897	66.84552875	36.86178848
	13.54471545	14.21138226	60.59349565	39.69918643
	13.96747967	15.84552927	77.77235715	35.26829319

Tukey's multiple comparisons test	Mean Diff.	95.00% CI of diff.	Below threshold?	Summary	Adjusted P Value	q	DF
Cont vs. Cont+Nec-1	0.168	-12.04 to 12.37	No	ns	>0.9999	0.06234	8
Cont vs. Cort	-53.26	-65.46 to -41.05	Yes	****	<0.0001	19.76	8
Cont vs. Cort+NEC1	-22.13	-34.34 to -9.925	Yes	**	0.0018	8.212	8
Cont+Nec-1 vs. Cort	-53.43	-65.63 to -41.22	Yes	****	<0.0001	19.82	8
Cont+Nec-1 vs. Cort+NEC1	-22.3	-34.50 to -10.09	Yes	**	0.0017	8.274	8
Cort vs. Cort+NEC1	31.13	18.92 to 43.33	Yes	***	0.0002	11.55	8

GSK872 reduce Cort-induced increase in inflammatory cytokines in human astrocytes.

B

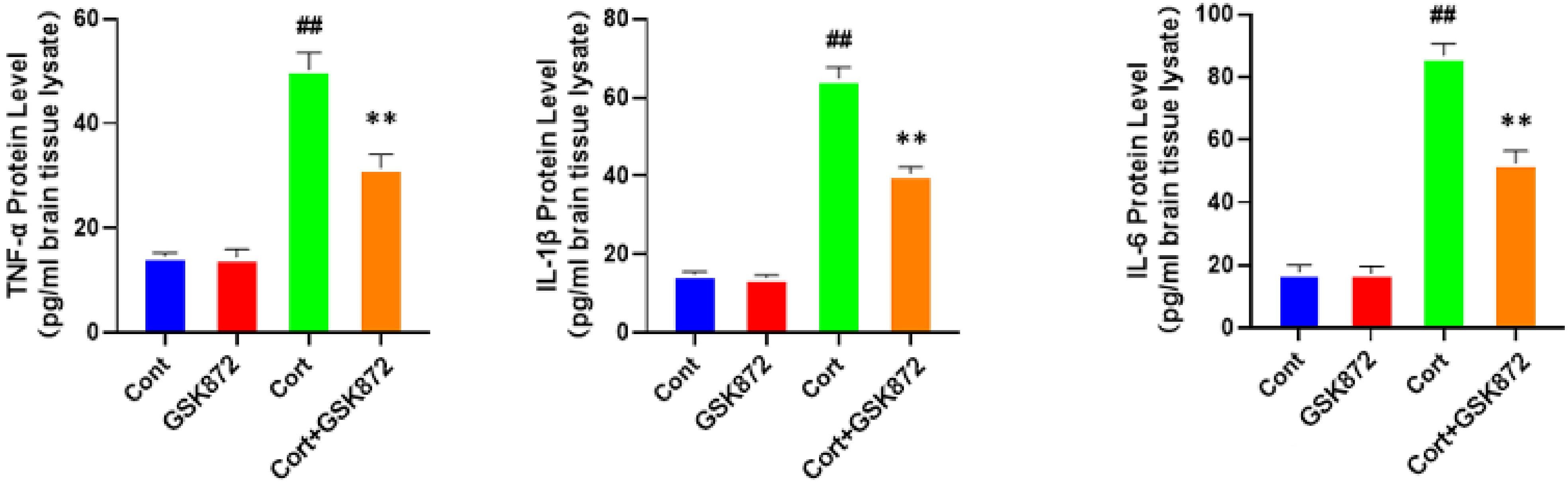

GSK872 reduce Cort-induced increase in inflammatory cytokines in human astrocytes.

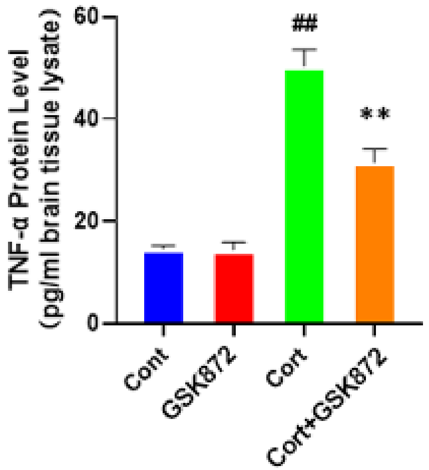

GSK				
TNF-α	Cont	Cont+GSK	Cort	Cort+GSK
OD VALUE	0.2167	0.2018	0.7732	0.446
	0.201	0.1914	0.7055	0.4907
	0.2135	0.2305	0.6818	0.4178
Protein level	15.15384615	14.11188811	54.06993007	31.18881119
	14.05594406	13.38461538	49.33566434	34.31468531
	14.93006993	16.11888112	47.67832168	29.21678322

Tukey's multiple comparisons test	Mean Diff.	95.00% CI of diff.	Below threshold?	Summary	Adjusted P Value	q	DF
Cont vs. Cont+GSK	0.1748	-5.665 to 6.014	No	ns	0.9997	0.1356	8
Cont vs. Cort	-35.65	-41.49 to -29.81	Yes	****	<0.0001	27.65	8
Cont vs. Cort+GSK	-16.86	-22.70 to -11.02	Yes	****	<0.0001	13.08	8
Cont+GSK vs. Cort	-35.82	-41.66 to -29.98	Yes	****	<0.0001	27.78	8
Cont+GSK vs. Cort+GSK	-17.03	-22.87 to -11.20	Yes	****	<0.0001	13.21	8
Cort vs. Cort+GSK	18.79	12.95 to 24.63	Yes	****	<0.0001	14.57	8

GSK872 reduce Cort-induced increase in inflammatory cytokines in human astrocytes.

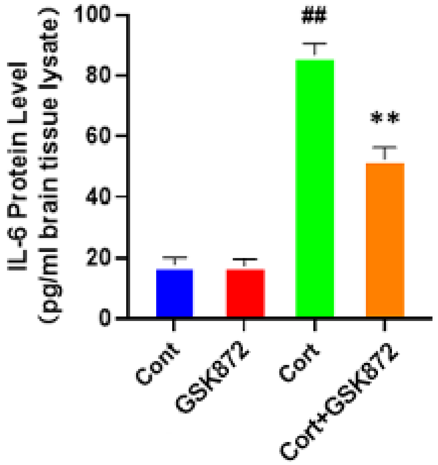

GSK				
IL-6	Cont	Cont+GSK	Cort	Cort+GSK
OD VALUE	0.2259	0.2878	1.3142	0.7915
	0.2845	0.2234	1.2045	0.6973
	0.2721	0.2533	1.264	0.8006
Protein level	15.57931034	19.84827586	90.63448276	54.5862069
	19.62068966	15.40689655	83.06896552	48.08965517
	18.76551724	17.46896552	87.17241379	55.2137931

Tukey's multiple comparisons test	Mean Diff.	95.00% CI of diff.	Below threshold?	Summary	Adjusted P Value	q	DF
Cont vs. Cont+GSK	0.4138	-7.790 to 8.618	No	ns	0.9984	0.2284	8
Cont vs. Cort	-68.97	-77.17 to -60.77	Yes	****	<0.0001	38.07	8
Cont vs. Cort+GSK	-34.64	-42.85 to -26.44	Yes	****	<0.0001	19.12	8
Cont+GSK vs. Cort	-69.38	-77.59 to -61.18	Yes	****	<0.0001	38.3	8
Cont+GSK vs. Cort+GSK	-35.06	-43.26 to -26.85	Yes	****	<0.0001	19.35	8
Cort vs. Cort+GSK	34.33	26.12 to 42.53	Yes	****	<0.0001	18.95	8

GSK872 reduce Cort-induced increase in inflammatory cytokines in human astrocytes.

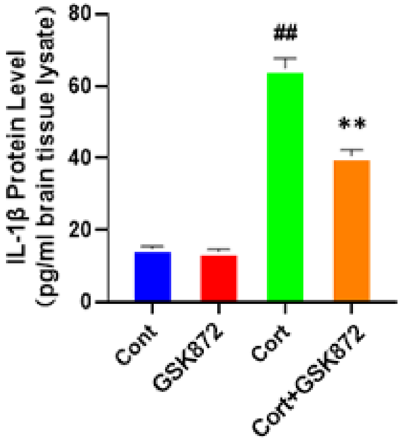

GSK				
IL-1β	Cont	Cont+GSK	Cort	Cort+GSK
OD VALUE	0.2104	0.209600002	0.7962	0.529000002
	0.2078	0.1963	0.8245	0.550399995
	0.2219	0.205400005	0.8647	0.511199993
Protein level	14.69918699	14.63414647	62.32520325	40.60162619
	14.48780488	13.55284552	64.62601626	42.341463
	15.63414634	14.29268333	67.89430894	39.15447102

Tukey's multiple comparisons test	Mean Diff.	95.00% CI of diff.	Below threshold?	Summary	Adjusted P Value	q	DF
Cont vs. Cont+GSK	0.7805	-3.567 to 5.127	No	ns	0.9368	0.8131	8
Cont vs. Cort	-50.01	-54.36 to -45.66	Yes	****	<0.0001	52.1	8
Cont vs. Cort+GSK	-25.76	-30.11 to -21.41	Yes	****	<0.0001	26.84	8
Cont+GSK vs. Cort	-50.79	-55.14 to -46.44	Yes	****	<0.0001	52.91	8
Cont+GSK vs. Cort+GSK	-26.54	-30.89 to -22.19	Yes	****	<0.0001	27.65	8
Cort vs. Cort+GSK	24.25	19.90 to 28.60	Yes	****	<0.0001	25.26	8

The necroptotic kinase inhibitors reduce Cort-induced increase in inflammatory cytokines in human astrocytes.

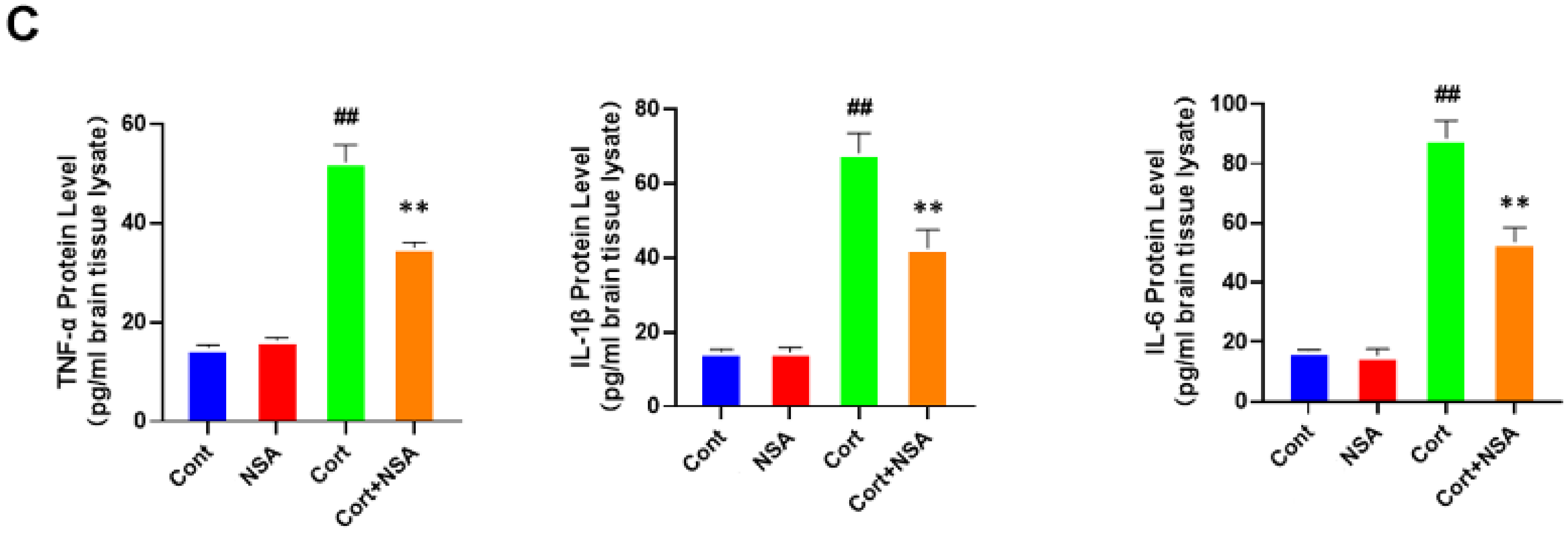

NSA reduce Cort-induced increase in inflammatory cytokines in human [illegible]

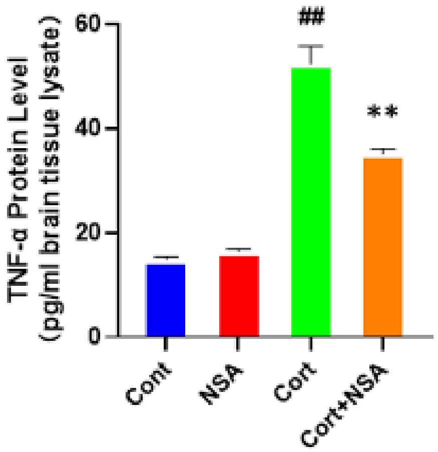

NSA				
TNF-α	Cont	Cont+NSA	Cort	Cort+NSA
OD VALUE	0.2061	0.2336	0.7682	0.5198
	0.2179	0.2292	0.783	0.4913
	0.2168	0.2419	0.6934	0.4939
Protein level	14.41258741	16.33566434	53.72027972	36.34965035
	15.23776224	16.02797203	54.75524476	34.35664336
	15.16083916	16.91608392	48.48951049	34.53846154

Tukey's multiple comparisons test	Mean Diff.	95.00% CI of diff.	Below threshold?	Summary	Adjusted P Value	q	DF
Cont vs. Cont+NSA	-1.489	-6.186 to 3.208	No	ns	0.7456	1.436	8
Cont vs. Cort	-37.38	-42.08 to -32.69	Yes	****	<0.0001	36.05	8
Cont vs. Cort+NSA	-20.14	-24.84 to -15.45	Yes	****	<0.0001	19.42	8
Cont+NSA vs. Cort	-35.9	-40.59 to -31.20	Yes	****	<0.0001	34.61	8
Cont+NSA vs. Cort+NSA	-18.66	-23.35 to -13.96	Yes	****	<0.0001	17.99	8
Cort vs. Cort+NSA	17.24	12.54 to 21.94	Yes	****	<0.0001	16.62	8

NSA reduce Cort-induced increase in inflammatory cytokines in human astrocytes.

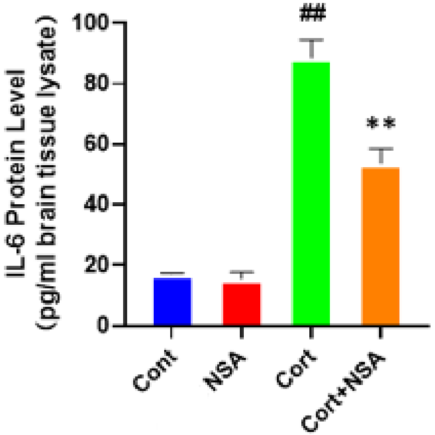

NSA				
IL-6	Cont	Cont+NSA	Cort	Cort+NSA
OD VALUE	0.2501	0.2577	1.2245	0.8469
	0.2463	0.1967	1.3826	0.7794
	0.2489	0.2217	1.2487	0.7107
Protein level	17.24827586	17.77241379	84.44827586	58.40689655
	16.9862069	13.56551724	95.35172414	53.75172414
	17.16551724	15.28965517	86.11724138	49.0137931

Tukey's multiple comparisons test	Mean Diff.	95.00% CI of diff.	Below threshold?	Summary	Adjusted P Value	q	DF
Cont vs. Cont+NSA	1.591	-8.623 to 11.80	No	ns	0.9571	0.7053	8
Cont vs. Cort	-71.51	-81.72 to -61.29	Yes	****	<0.0001	31.7	8
Cont vs. Cort+NSA	-36.59	-46.80 to -26.38	Yes	****	<0.0001	16.22	8
Cont+NSA vs. Cort	-73.1	-83.31 to -62.88	Yes	****	<0.0001	32.41	8
Cont+NSA vs. Cort+NSA	-38.18	-48.40 to -27.97	Yes	****	<0.0001	16.93	8
Cort vs. Cort+NSA	34.91	24.70 to 45.13	Yes	****	<0.0001	15.48	8

NSA reduce Cort-induced increase in inflammatory cytokines in human astrocytes

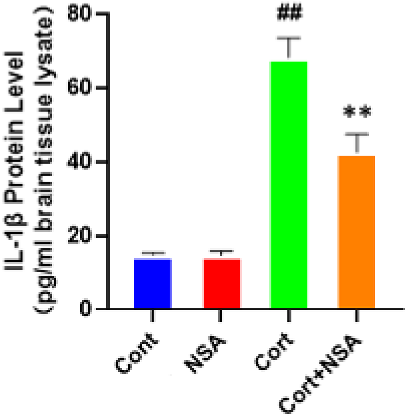

NSA				
IL-1β	Cont	Cont+NSA	Cort	Cort+NSA
OD VALUE	0.2048	0.222800002	0.8963	0.607399994
	0.2144	0.213100001	0.7946	0.562299997
	0.2163	0.194299996	0.9145	0.488700005
Protein level	14.24390244	15.7073172	70.46341463	46.97560928
	15.02439024	14.91869929	62.19512195	43.30894286
	15.17886179	13.39024357	71.94308943	37.32520369

Tukey's multiple comparisons test	Mean Diff.	95.00% CI of diff.	Below threshold?	Summary	Adjusted P Value	q	DF
Cont vs. Cont+NSA	0.1436	-9.371 to 9.658	No	ns	>0.9999	0.06834	8
Cont vs. Cort	-53.38	-62.90 to -43.87	Yes	****	<0.0001	25.41	8
Cont vs. Cort+NSA	-27.72	-37.24 to -18.21	Yes	****	<0.0001	13.19	8
Cont+NSA vs. Cort	-53.53	-63.04 to -44.01	Yes	****	<0.0001	25.48	8
Cont+NSA vs. Cort+NSA	-27.86	-37.38 to -18.35	Yes	****	<0.0001	13.26	8
Cort vs. Cort+NSA	25.66	16.15 to 35.18	Yes	***	0.0001	12.22	8

Fluoxetine reduces the CUMS induced increase of inflammatory cytokines in the mice hippocampus.

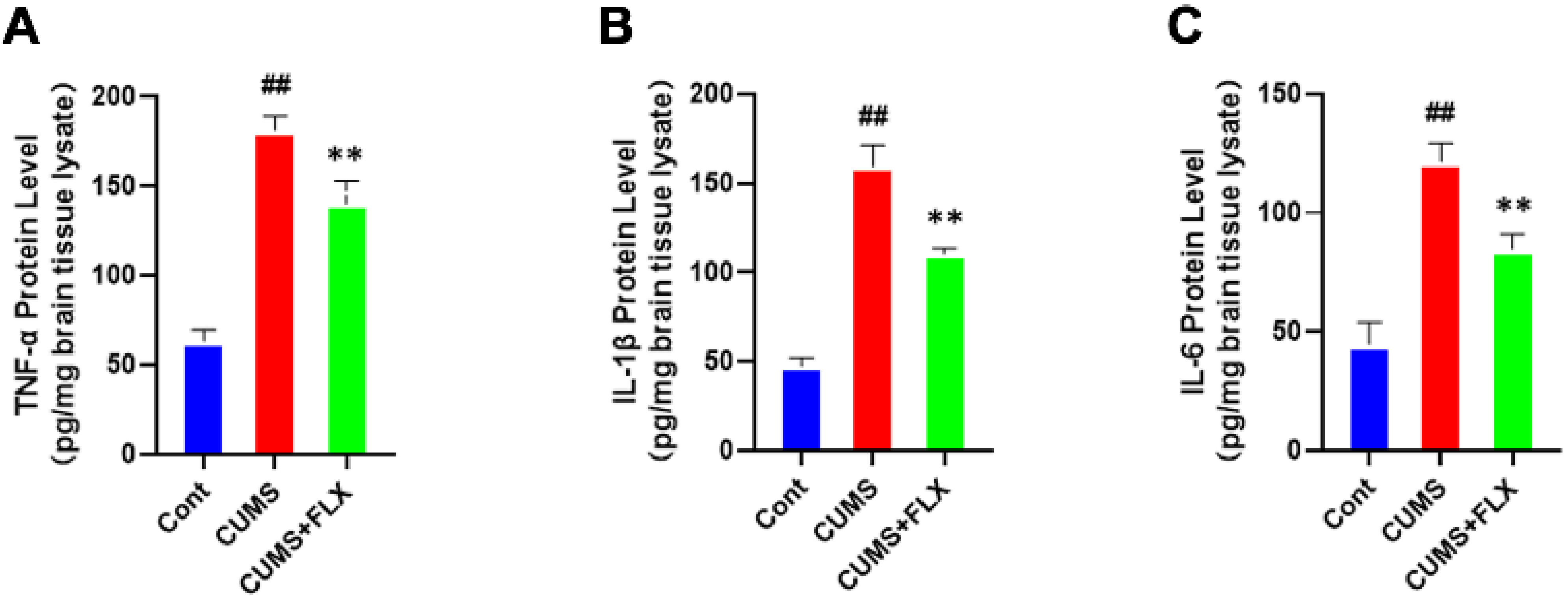

Fluoxetine reduces the CUMS induced increase of inflammatory cytokines in the mice hippocampus.

A

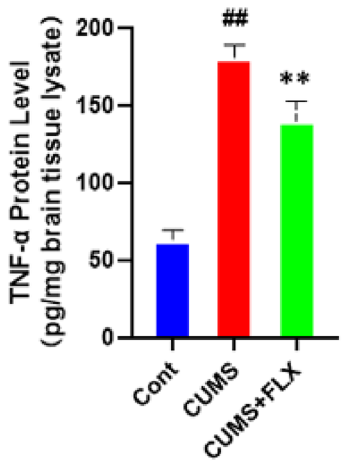

Groups	TNF-α
	pg/ml
**Cont**	73.3636366
**Cont**	190.3636364
**Cont**	154
**Cont**	66.66363636
**CUMS+Saline**	177.4545455
**CUMS+Saline**	137.4545455
**CUMS+Saline**	67.63636387
**CUMS+Saline**	218.3641375
**CUMS+FLX**	176.2819369
**CUMS+FLX**	56.45456636
**CUMS+FLX**	176.4949564
**CUMS+FLX**	129.0910192

Tukey's multiple comparisons test	Mean Diff.	95.00% CI of diff.	Below threshold?	Summary	Adjusted P Value	q	DP
Cont vs. CUMS	-117.9	-141.1 to -94.57	Yes	****	<0.0001	21.97	6
Cont vs. CUMS+FLX	-76.6	-99.88 to -53.32	Yes	***	0.0001	14.28	6
CUMS vs. CUMS+FLX	41.26	17.98 to 64.54	Yes	**	0.0039	7.69	6

Fluoxetine reduces the CUMS induced increase of inflammatory cytokines in the mice hippocampus.

B

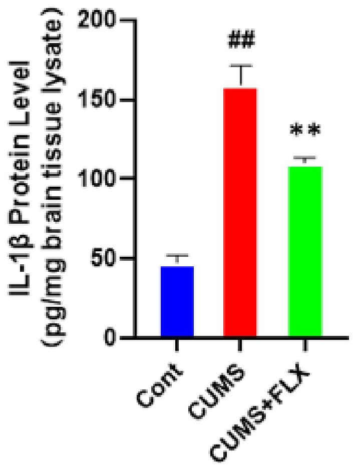

Groups	IL-1β
	pg/ml
**Cont**	44.8181812
**Cont**	146.1818184
**Cont**	110.0000033
**Cont**	53.09090627
**CUMS+Saline**	164.0000269
**CUMS+Saline**	116.3636371
**CUMS+Saline**	45.21309878
**CUMS+Saline**	142.7089509
**CUMS+FLX**	108.0083214
**CUMS+FLX**	54.98718561
**CUMS+FLX**	168.4658764
**CUMS+FLX**	113.9252354

Tukey's multiple comparisons test	Mean Diff.	95.00% CI of diff.	Below threshold?	Summary	Adjusted P Value	q	DF
Cont vs. CUMS	-111.8	-130.7 to -92.99	Yes	****	<0.0001	25.75	6
Cont vs. CUMS+FLX	-62.94	-81.79 to -44.09	Yes	***	0.0001	14.49	6
CUMS vs. CUMS+FLX	48.9	30.05 to 67.75	Yes	***	0.0005	11.26	6

Fluoxetine reduces the CUMS induced increase of inflammatory cytokines in the mice hippocampus.

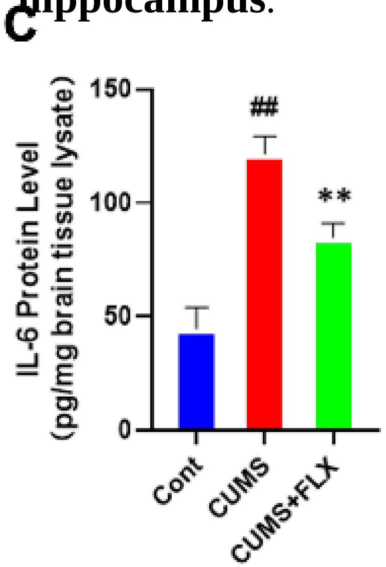

Groups	IL-6
	pg/ml
**Cont**	44.33162078
**Cont**	129.6995052
**Cont**	91.37499787
**Cont**	54.52254545
**CUMS+Saline**	114.8124994
**CUMS+Saline**	78.93749893
**CUMS+Saline**	36.08617635
**CUMS+Saline**	121.0388413
**CUMS+FLX**	94.56717703
**CUMS+FLX**	57.95133501
**CUMS+FLX**	110.5555989
**CUMS+FLX**	84.49824414

Tukey's multiple comparisons test	Mean Diff.	95.00% CI of diff.	Below threshold?	Summary	Adjusted P Value	q	DP
Cont vs. CUMS	-76.87	-96.28 to -57.46	Yes	****	<0.0001	17.19	6
Cont vs. CUMS+FLX	-39.96	-59.36 to -20.55	Yes	**	0.0018	8.934	6
CUMS vs. CUMS+FLX	36.91	17.51 to 56.32	Yes	**	0.0027	8.254	6

Fluoxetine reduces the Cort-induced increase of inflammatory cytokines in the human astrocytes

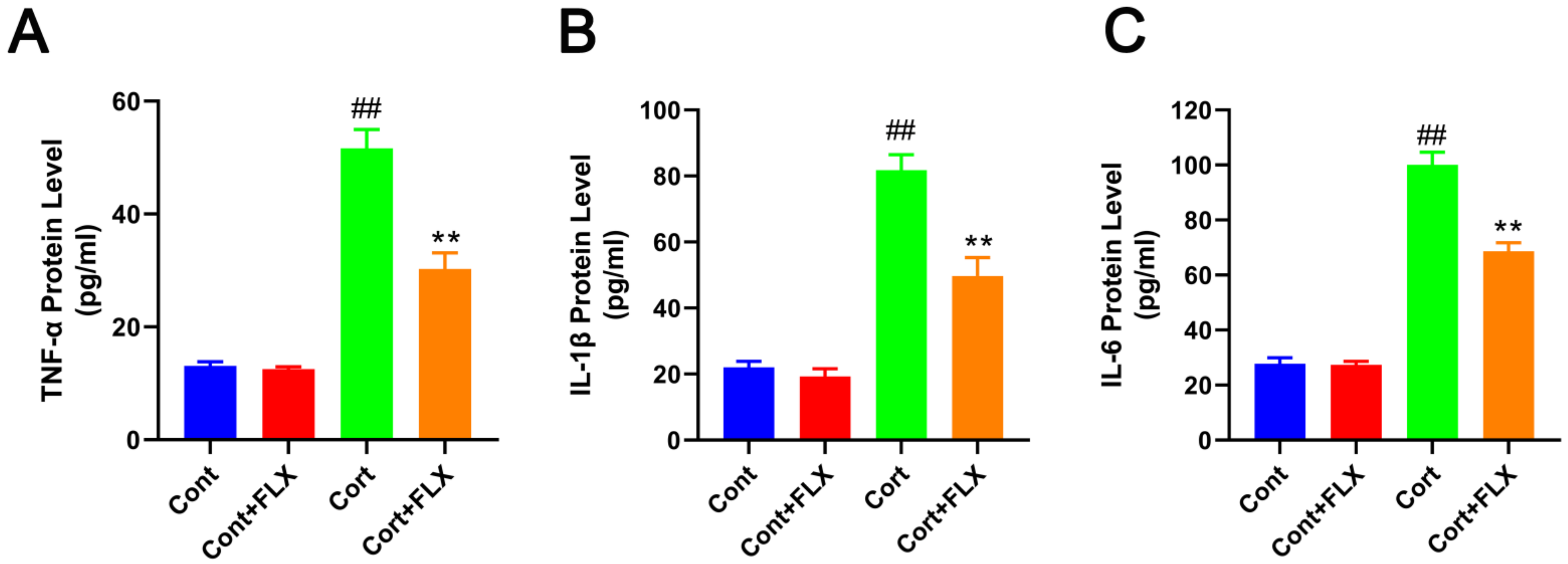

Fluoxetine reduces the Cort-induced increase of inflammatory cytokines in the human astrocytes

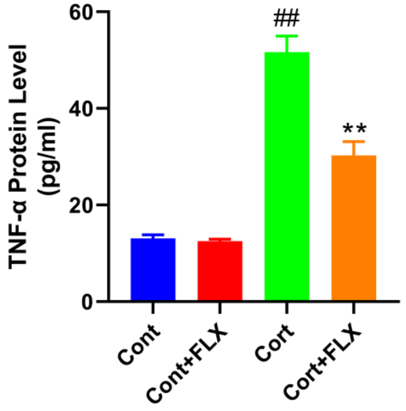

TNF-α									
	Cont	Cont+FLX	Cort	Cort+FLX		Cont	Cont+FLX	Cort	Cort+FLX
OD VALUE	0.2133	0.2001	0.7286	0.4371	Protein level	14.91608392	13.99300699	50.95104895	30.56643357
	0.2264	0.2215	0.7369	0.4136		15.83216783	15.48951049	51.53146853	28.92307692
	0.2175	0.2346	0.6394	0.4368		15.20979021	16.40559441	44.71328671	30.54545455

Tukey's multiple comparisons test	Mean Diff.	95.00% CI of diff.	Below threshold?	Summary	Adjusted P Value	q	DF
Cont vs. Cont+FLX	0.02331	-5.348 to 5.395	No	ns	>0.9999	0.01965	8
Cont vs. Cort	-33.75	-39.12 to -28.37	Yes	****	<0.0001	28.45	8
Cont vs. Cort+FLX	-14.69	-20.06 to -9.321	Yes	***	0.0001	12.39	8
Cont+FLX vs. Cort	-33.77	-39.14 to -28.40	Yes	****	<0.0001	28.47	8
Cont+FLX vs. Cort+FLX	-14.72	-20.09 to -9.344	Yes	***	0.0001	12.41	8
Cort vs. Cort+FLX	19.05	13.68 to 24.42	Yes	****	<0.0001	16.07	8

Fluoxetine reduces the Cort-induced increase of inflammatory cytokines in the human astrocytes

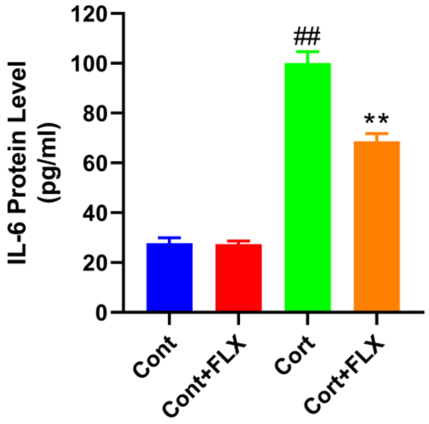

IL-6									
	**Cont**	**Cont+FLX**	**Cort**	**Cort+FLX**		**Cont**	**Cont+FLX**	**Cort**	**Cort+FLX**
**OD VALUE**	0.2459	0.2273	1.2248	0.7233	**Protein level**	16.95862069	15.67586207	84.46896552	49.88275862
	0.2378	0.2592	1.2359	0.82521		16.4	17.87586207	85.23448276	56.91103448
	0.2096	0.2028	1.147	0.8112		14.45517241	13.9862069	79.10344828	55.94482759

Tukey's multiple comparisons test	Mean Diff.	95.00% CI of diff.	Below threshold?	Summary	Adjusted P Value	q	DF
Cont vs. Cont+FLX	0.09195	-7.211 to 7.395	No	ns	>0.9999	0.05702	8
Cont vs. Cort	-67	-74.30 to -59.69	Yes	****	<0.0001	41.55	8
Cont vs. Cort+FLX	-38.31	-45.61 to -31.01	Yes	****	<0.0001	23.76	8
Cont+FLX vs. Cort	-67.09	-74.39 to -59.79	Yes	****	<0.0001	41.6	8
Cont+FLX vs. Cort+FLX	-38.4	-45.70 to -31.10	Yes	****	<0.0001	23.81	8
Cort vs. Cort+FLX	28.69	21.39 to 35.99	Yes	****	<0.0001	17.79	8

Fluoxetine reduces the Cort-induced increase of inflammatory cytokines in the human astrocytes

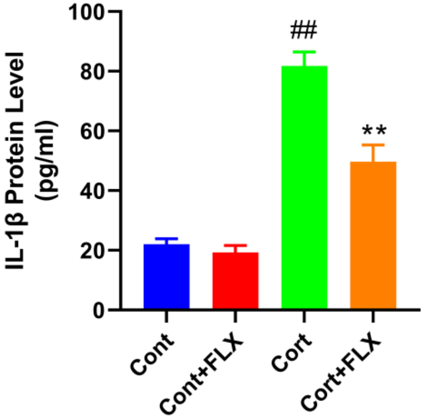

IL-1β									
	Cont	Cont+FLX	Cort	Cort+FLX		Cont	Cont+FLX	Cort	Cort+FLX
OD VALUE	0.205000036	0.230700001	0.8742	0.499999993	Protein level	14.26016557	16.34959359	68.66666667	38.24390191
	0.204899997	0.195500001	0.8512	0.506500003		14.25203224	13.48780498	66.79674797	38.77235799
	0.186199993	0.186100006	0.8109	0.453699994		12.73170674	12.72357773	63.5203252	34.47967432

Tukey's multiple comparisons test	Mean Diff.	95.00% CI of diff.	Below threshold?	Summary	Adjusted P Value	q	DF
Cont vs. Cont+FLX	-0.439	-5.781 to 4.903	No	ns	0.9931	0.3722	8
Cont vs. Cort	-52.58	-57.92 to -47.24	Yes	****	<0.0001	44.58	8
Cont vs. Cort+FLX	-23.42	-28.76 to -18.08	Yes	****	<0.0001	19.85	8
Cont+FLX vs. Cort	-52.14	-57.48 to -46.80	Yes	****	<0.0001	44.21	8
Cont+FLX vs. Cort+FLX	-22.98	-28.32 to -17.64	Yes	****	<0.0001	19.48	8
Cort vs. Cort+FLX	29.16	23.82 to 34.50	Yes	****	<0.0001	24.73	8

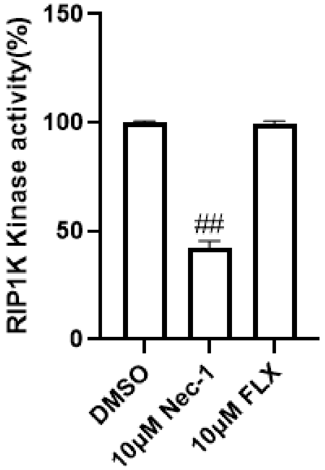

Number of families	1							
Number of comparisons per family	2							
Alpha	0.05							
Dunnett's multiple comparisons test	Mean Diff.	95.00% CI of diff.	Below threshold?	Summary	Adjusted P Value	A-?		
DMSO vs. 10μM Nec-1	57.78	52.84 to 62.72	Yes	****	<0.0001	B	10μM Nec-1	
DMSO vs. 10μM FLX	0.9162	-4.025 to 5.858	No	ns	0.8224	C	10μM FLX	
Test details	Mean 1	Mean 2	Mean Diff.	SE of diff.	n1	n2	q	DF
DMSO vs. 10μM Nec-1	100.0	42.22	57.78	1.726	3	3	33.48	6
DMSO vs. 10μM FLX	100.0	99.08	0.9162	1.726	3	3	0.5308	6
