# Supplementary material for: Necroptotic kinases are involved in the reduction of depression-induced astrocytes and fluoxetine’s inhibitory effects on necroptotic kinases
Source: Front Pharmacol. 2023 Jan 4;13:1060954. doi: 10.3389/fphar.2022.1060954 (PMC9847570; doi:10.3389/fphar.2022.1060954)
Supplement: Supplementary file 2 [file DataSheet2.DOCX]

**Supplementary material：**

**Table 1. Detailed schedule of induction stressors of the mouse CUMS model**

|  | **Monday** | **Tuesday** | **Wednesday** | **Thursday** | **Friday** | **Saturday** | **Sunday** |
| --- | --- | --- | --- | --- | --- | --- | --- |
| **The first week** | **FD** | **WD** | **CT** | **CL** | **WC** | **TS** | **BR** |
| **The second week** | **WD** | **CL** | **FD** | **TS** | **BR** | **WC** | **CT** |
| **The third week** | **TS** | **CL** | **BR** | **FD** | **WC** | **WD** | **CT** |
| **The fourth week** | **TS** | **WC** | **WD** | **BR** | **CT** | **CL** | **FD** |
| **The fifth week** | **WD** | **CT** | **CL** | **WC** | **TS** | **BR** | **FD** |

Food deprivation for 20 h（FD）

Water deprivation for 18 h（WD）

Cage tilting at 45° for 17 h (CT)

Continuous light for 36 h and forced swimming at 25 °C for 5 min (CL)

Wet cage for 21 h（WC）

Tail suspension for 6 min and forced swimming at 25 °C for 5 min (TS)

Behavior restriction for 2 h and horizontal shaking for 40 min (BR)

**Table 2.** **The antibodies for immunohistochemistry (IHC) analysis**

| Protein | Usage | Antibody |
| --- | --- | --- |
| RIP1K | **IHC** (1:200) | BD transduction, 610458 |
| RIP3K | **IHC** (1:200) | Abcam, ab62344 |
| GFAP | **IHC** (1:1000) | Sigma, Mab360 |
| BDNF | **IHC** (1:200) | Abcam, ab108319 |
| anti-Mouse IgG (H+L), Alexa Fluor ® 594 | **IHC** (1:1000) | ThermoFisher, **#**A-21203 |
| anti-Rabbit IgG (H+L), Alexa Fluor ® 488 | **IHC** (1:500) | ThermoFisher, **#**A-11008 |
| anti-Rabbit IgG (H+L), Alexa Fluor ® 594 | **IHC** (1:1000) | ThermoFisher, **#**A-11012 |
| anti-Mouse IgG (H+L), Alexa Fluor ® 488 | **IHC** (1:500) | ThermoFisher, **#**A-11001 |
| anti-Chicken IgY (H+L), Alexa Fluor ® 488 | **IHC** (1:500) | ThermoFisher, **#**A-21441 |

**Table 3.** **The antibodies for Western Blotting (WB) analysis**

| Protein | Usage | Antibody |
| --- | --- | --- |
| β-actin | WB (1:5000) | Sigma, A5441 |
| RIP1K | WB (1:200) | BD transduction, 610458 |
| p-RIP1K | WB (1:1000) | Cell Signaling Technology, #31122 |
| RIP3K | WB (1:1000) | Abcam, ab62344 |
| p-RIP3K | WB (1:1000) | Abcam, ab195117 |
| MLKL | WB (1:1000) | Biorbyt, orb32399 |
| p-MLKL | WB (1:1000) | Abcam, ab196436 |
| GFAP | WB (1:1000) | Sigma, Mab360 |
| BDNF | WB (1:1000) | Abcam, ab108319 |
| 5-HT1A receptor | WB (1:1000) | Gene Tex, GTX104703 |
| anti-mouse IgG (H+L) | WB (1:10000) | KPL, 042-06-18-06 |
| anti-rabbit IgG (H+L) | WB (1:10000) | KPL, 042-06-15-06 |


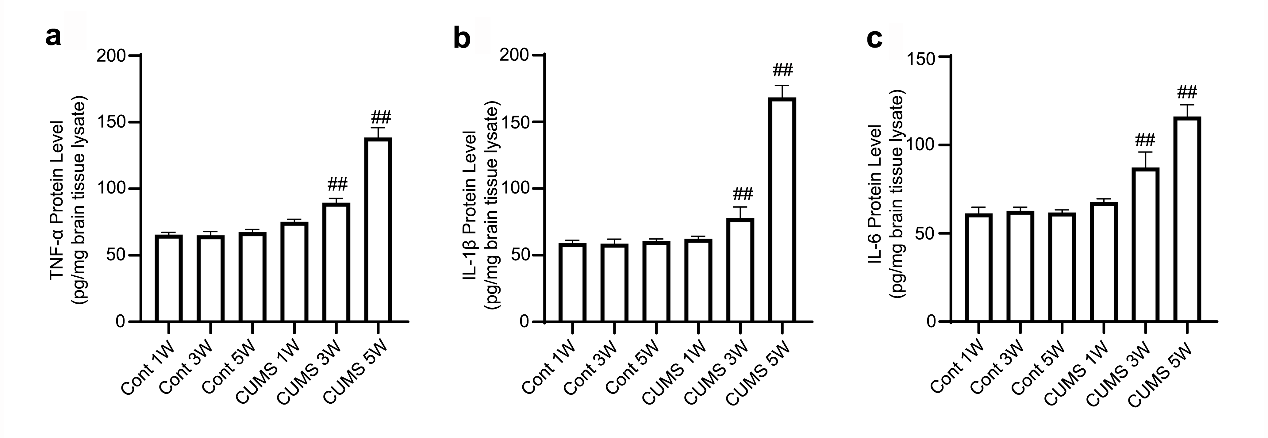


**Figure S1 CUMS treatment induces increases in the levels of inflammatory cytokines in the mouse hippocampus in a time-dependent manner.** The concentrations of TNF-α (a), IL-1β (b) and IL-6 (c) in mouse hippocampal tissue were measured by ELISA at different time points. Statistical analysis was carried out with one-way *ANOVA* followed by *Tukey’s test*. Mean *± SD,* n = 3 per group, *## P <* 0.01 vs. Cont. group.


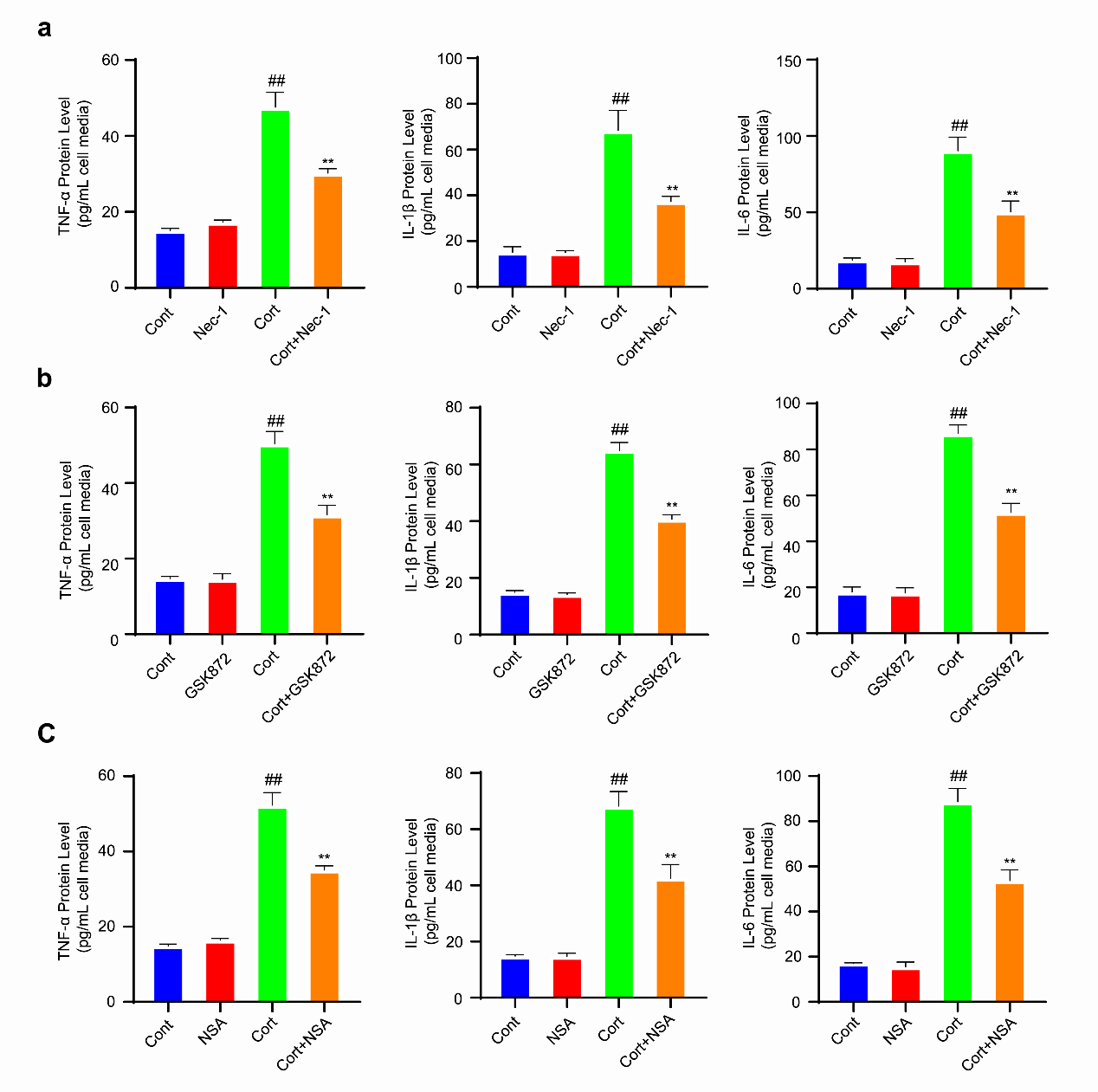


**Figure S2 Necroptotic kinase inhibitors reduce Cort-induced release of inflammatory cytokines from HA.** HA was exposed to 200 μM Cort for 1 h to induce cell injury. HA was treated with 100 μM Nec-1 (a), 10 μM GSK872 (b) or 1 μM NSA (c) during Cort treatment. The levels of TNF-α, IL-1β and IL-6 were measured by ELISA. Data are expressed as the mean *± SD,* n = 3 per group, *## P <* 0.01 vs Cont. group, *** P <* 0.01 vs Cort. group. Statistical analysis was performed by one-way *ANOVA* with *Tukey’s test.*

*
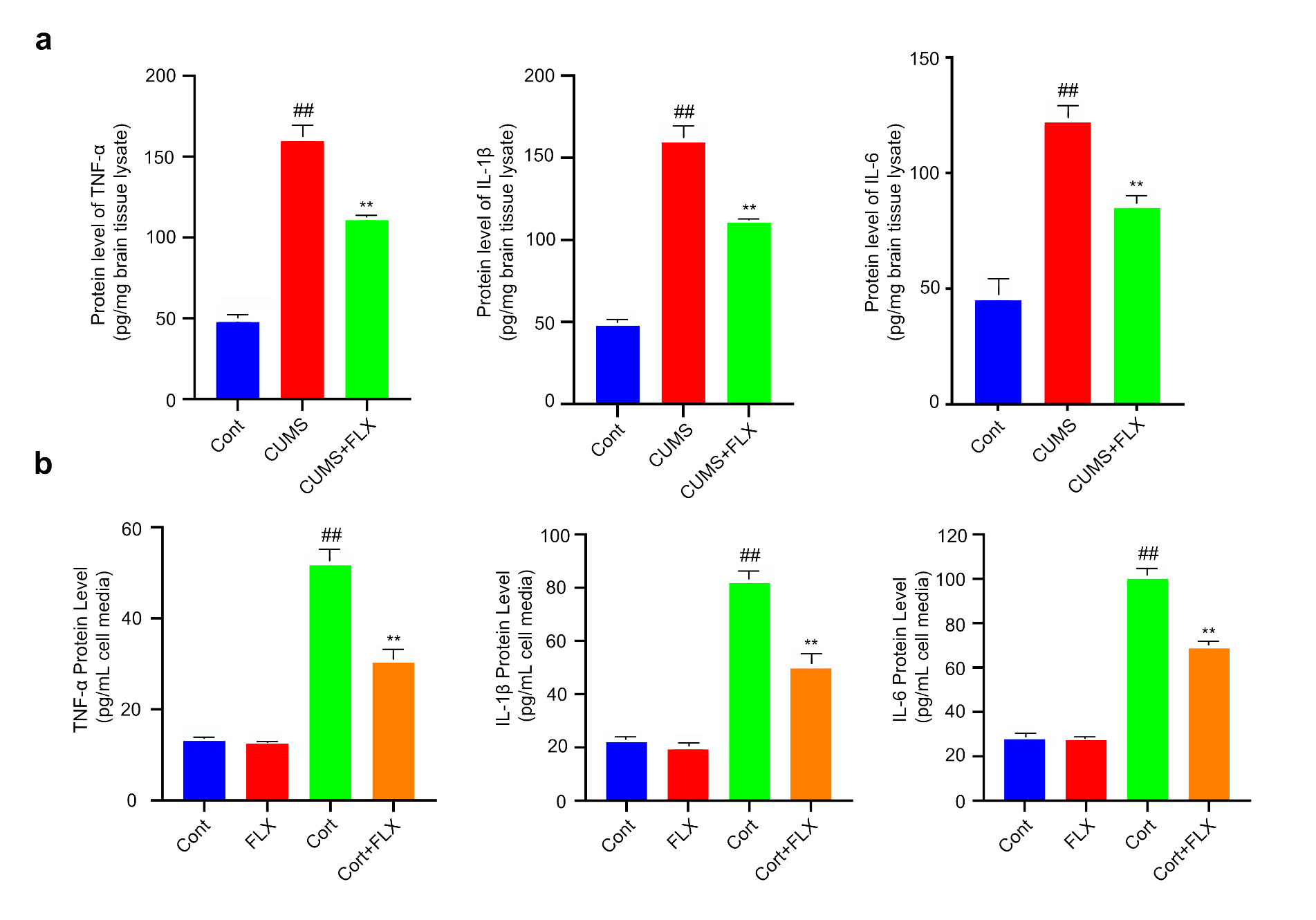
*

**Figure S3 FLX reduces the CUMS-induced or Cort-induced increases in inflammatory cytokines *in vivo and in vitro***. (a) The mice were treated with CUMS and FLX (10 mg/kg) for 5 weeks, and FLX was administered intraperitoneally every day. The concentrations of TNF-α, IL-1β and IL-6 in the mouse hippocampus were measured by ELISA. Statistical analysis was carried out with one-way *ANOVA* followed by *Tukey’s test*. Mean *± SD,* n = 3*, ## P <* 0.01 vs. Cont. group, *** P <* 0.01 vs. CUMS. group. (b) HA was treated with 200 μM Cort, 1 μM FLX or 200 μM Cort cotreated with 1 μM FLX for 1 h. The concentrations of TNF-α, IL-1β and IL-6 in Cont- or Cort-treated HA were measured by ELISA. Statistical analysis was carried out with one-way *ANOVA* followed by *Tukey’s test*. Mean *± SD, n = 3, ## P <* 0.01 vs Cont. group, *** P <* 0.01 vs Cort. group.


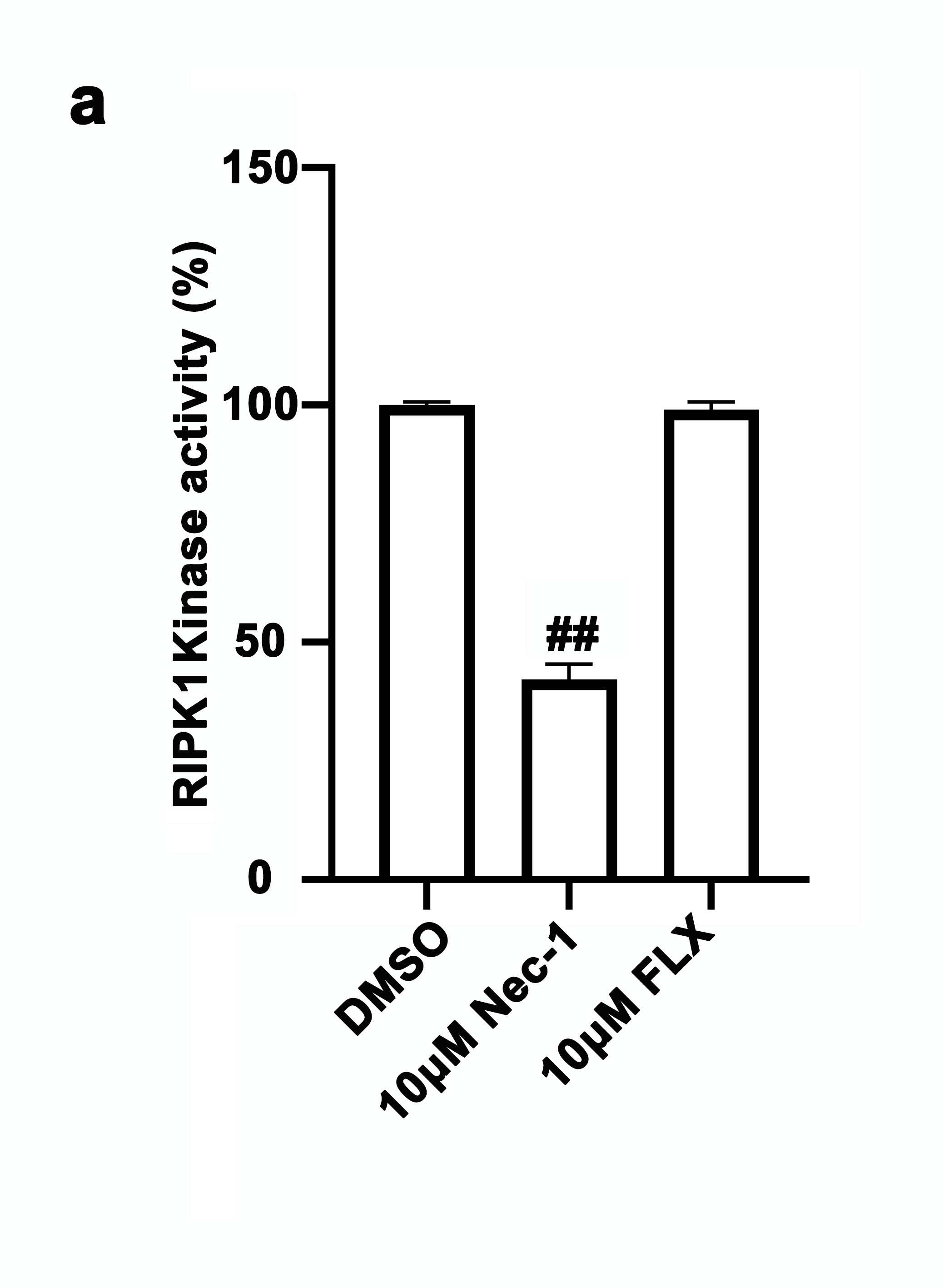


**Figure S4 FLX has no direct inhibitory effect on RIP1K phosphorylation.** RIPK1 kinase activity was detected using an in vitro ADP-Glo kinase assay. Statistical results were performed by one-way *ANOVA* with Tukey’s test and expressed as the mean ± *SD*. *n* = 3*,* ## *P*＜0.01 vs. DMSO. group.
